# Supplementary material for: New Guaiane-Type Sesquiterpenoids Biscogniauxiaols A–G with Anti-Fungal and Anti-Inflammatory Activities from the Endophytic Fungus Biscogniauxia Petrensis
Source: J Fungi (Basel). 2023 Mar 23;9(4):393. doi: 10.3390/jof9040393 (PMC10144765; doi:10.3390/jof9040393)
Supplement: Supplementary file 1 [file jof-09-00393-s001.zip › jof-2287784-supplementary.pdf]

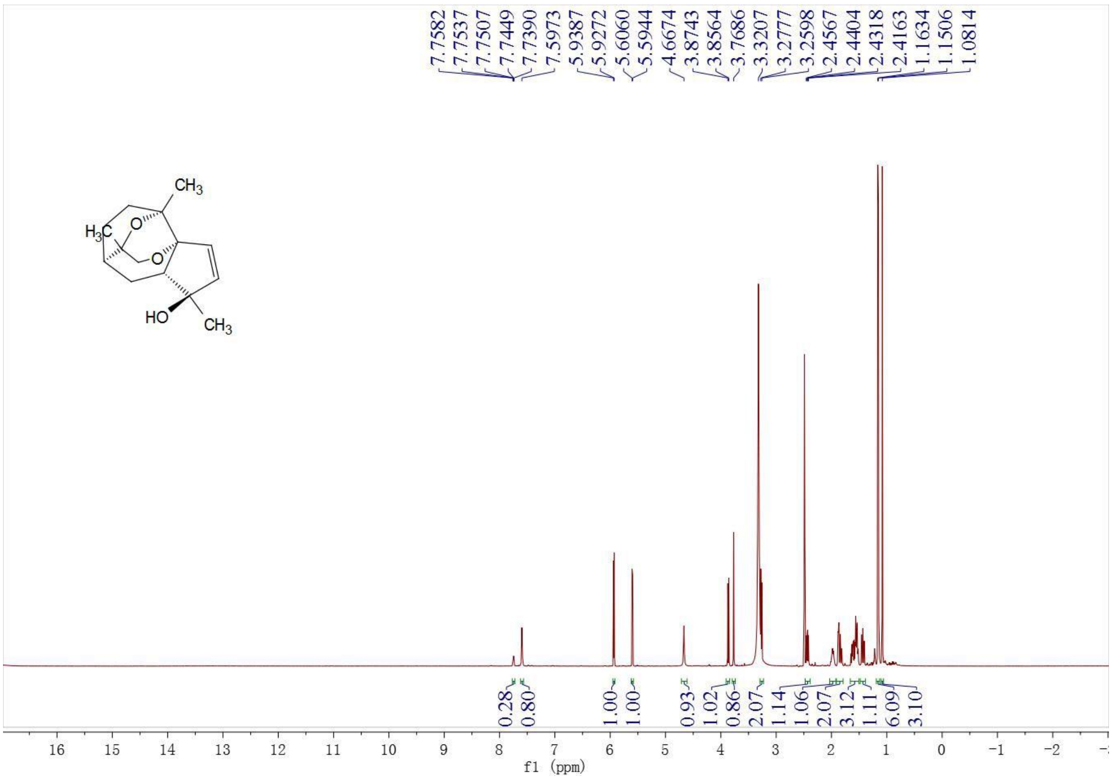

Figure S1. ^{1}H NMR spectrum of compound **1** in DMSO-*d6*.

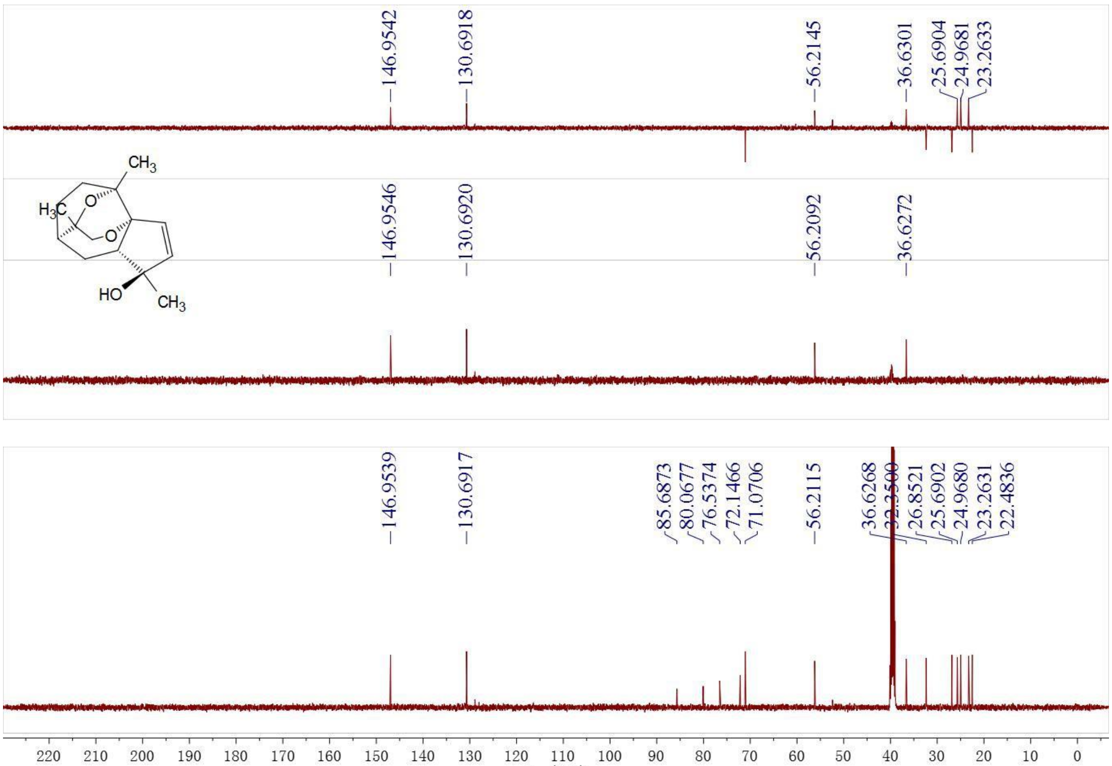

Figure S2. ^{13}C NMR and DEPT spectrum of compound **1** in DMSO-*d6*.

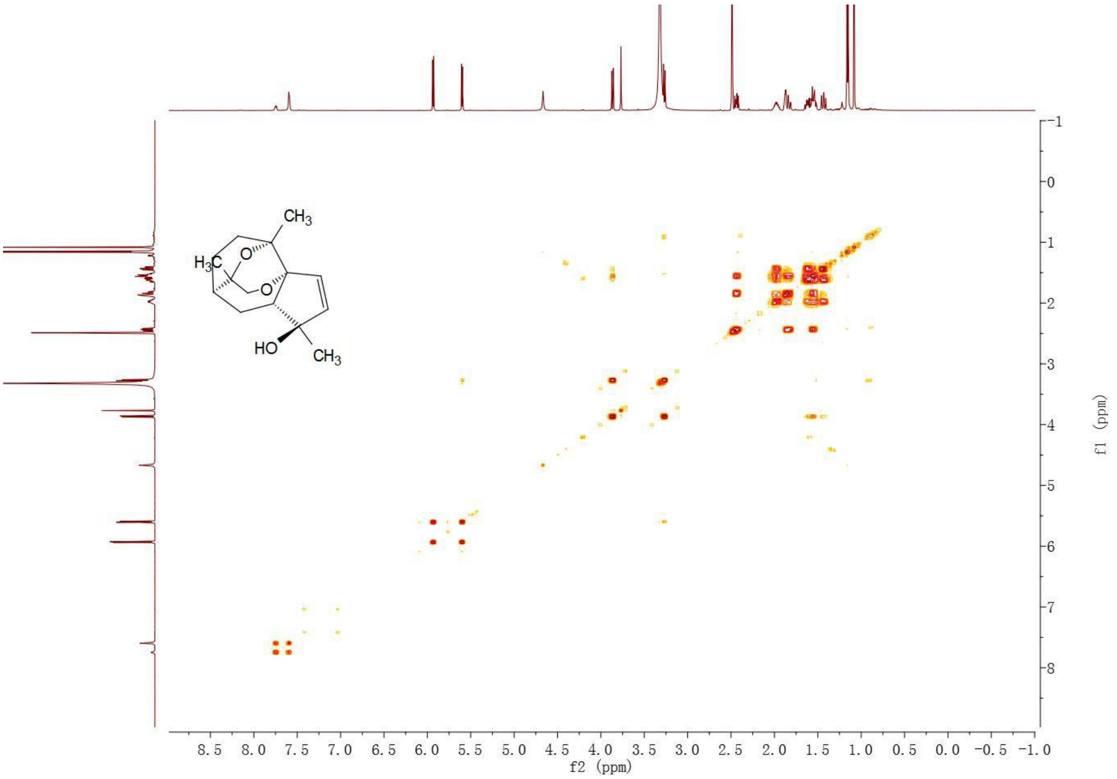

Figure S3. ^{1}H-^{1}H COSY spectrum of compound **1** in DMSO-*d6*.

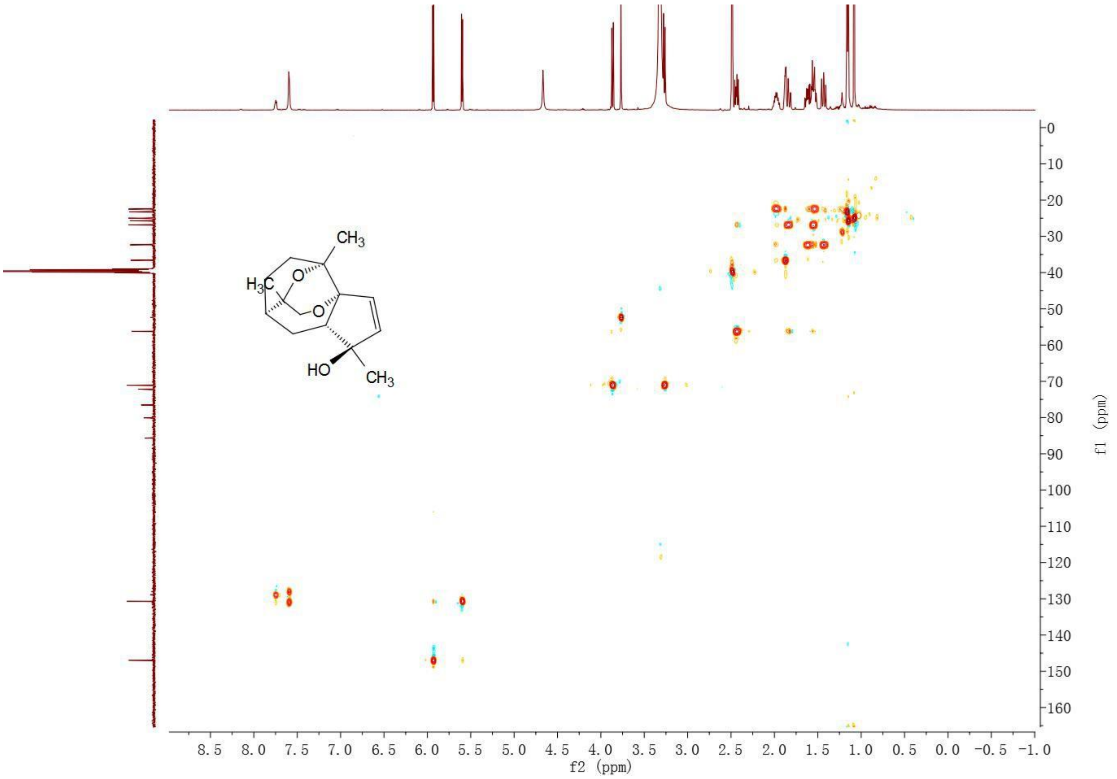

Figure S4. HSQC spectrum of compound **1** in DMSO-*d6*.

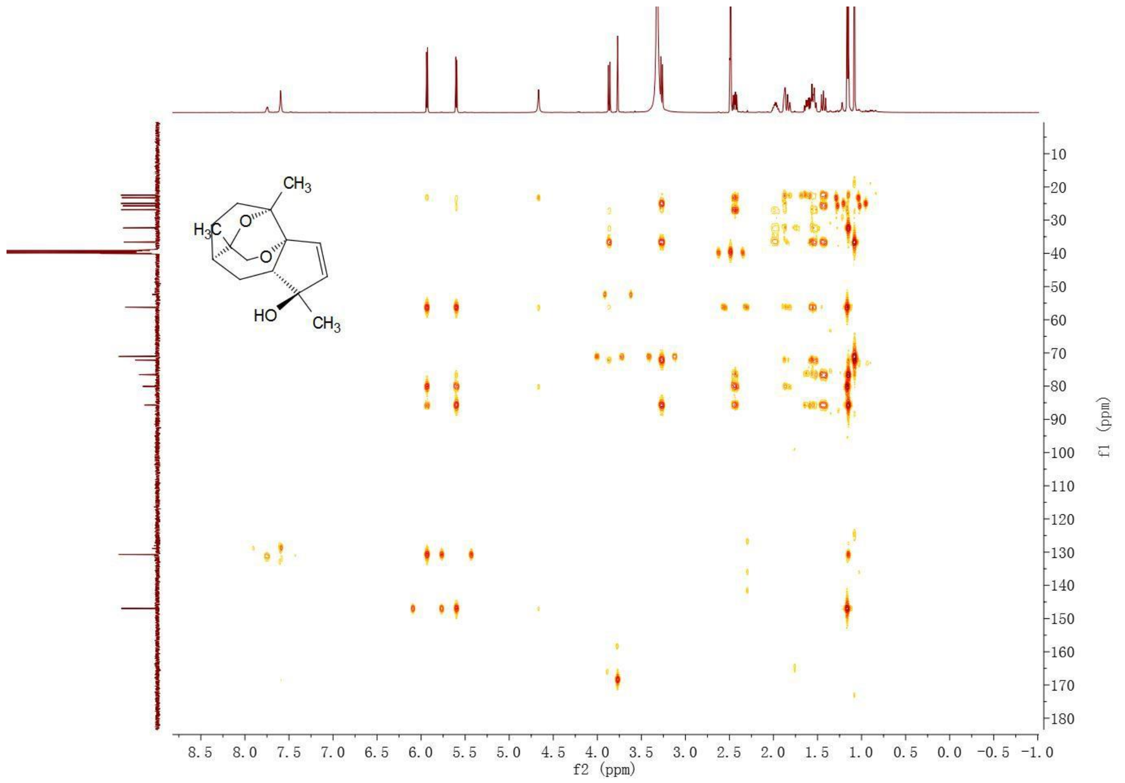

Figure S5. HMBC spectrum of compound **1** in DMSO-*d6*.

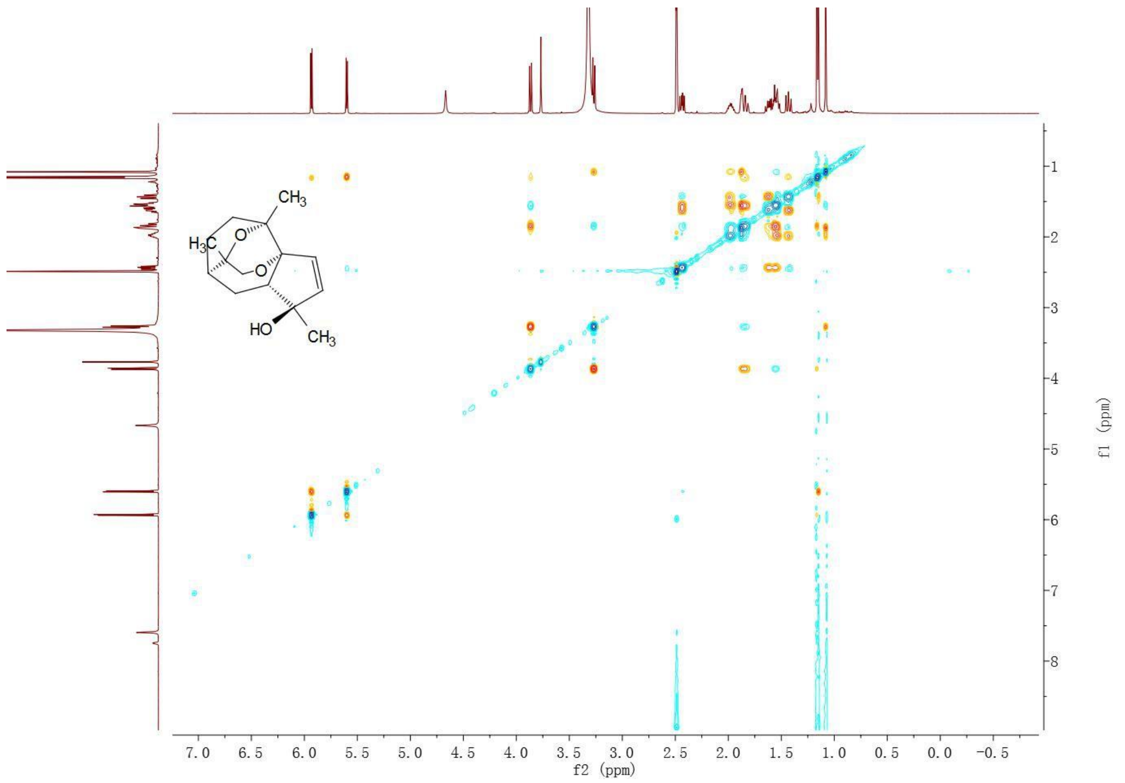

Figure S6. ROESY spectrum of compound **1** in DMSO-*d6*.

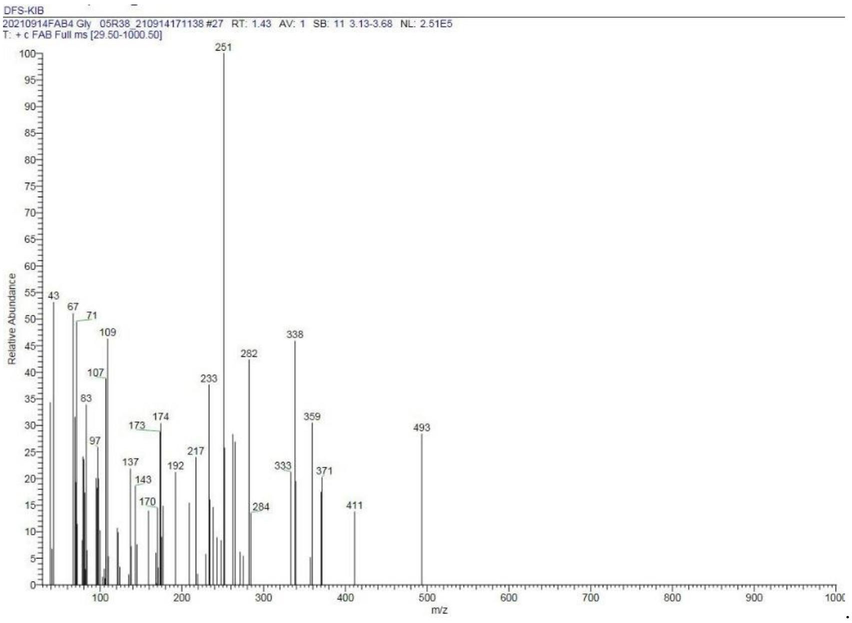

Figure S7. HRFABMS spectrum of compound **1.**

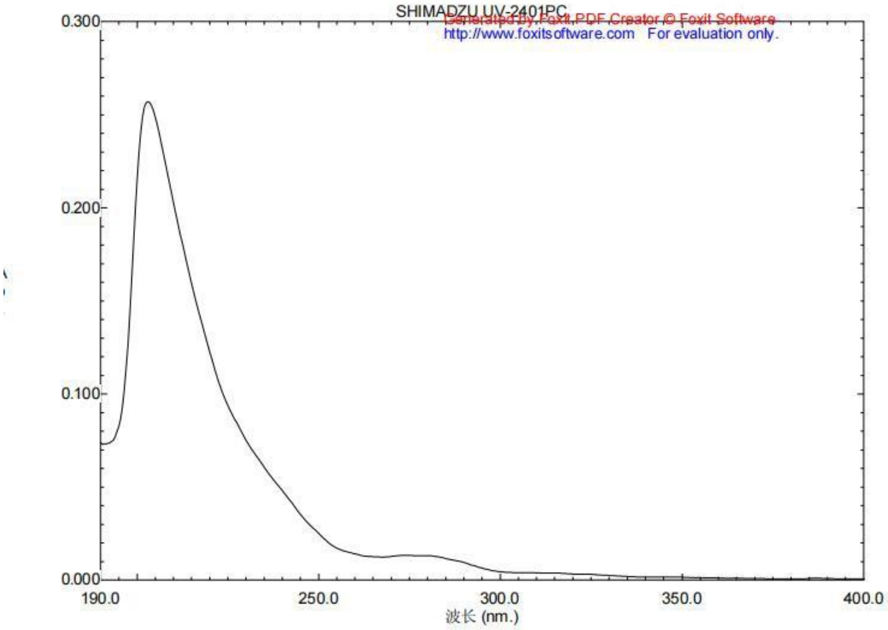

Figure S8. UV spectrum of **1** in methanol.

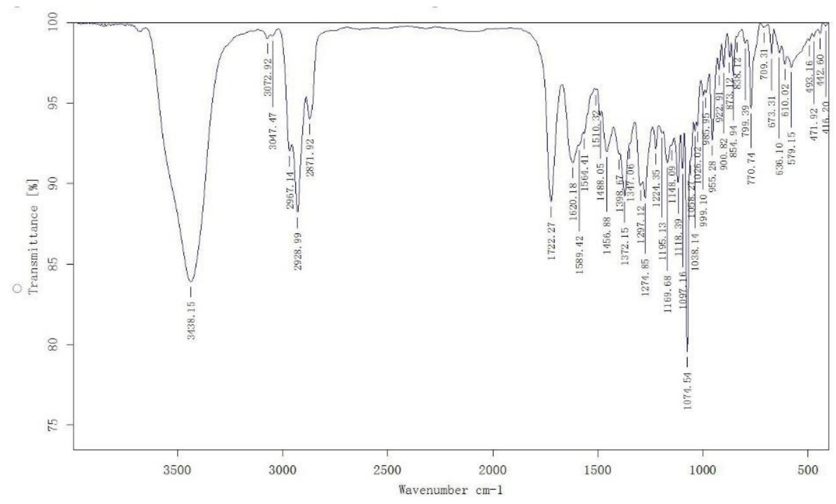

Figure S9. IR spectrum of **1** in methanol.

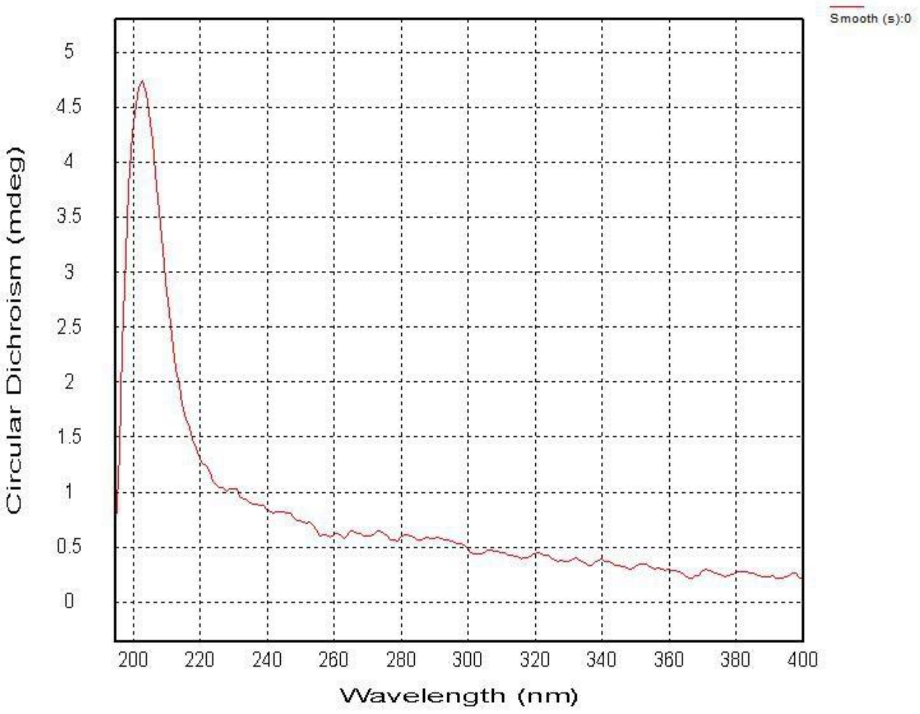

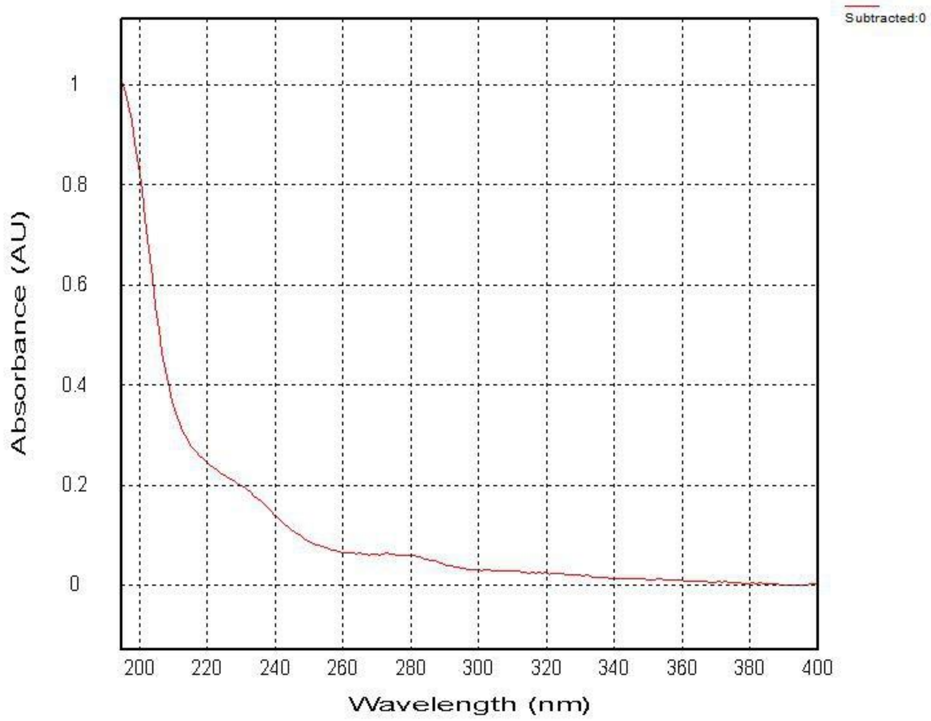

Figure S10. CD spectrum of **1** in methanol.

Rudolph Research Analytical

This sample was measured on an Autopol VI, Serial #91058
Manufactured by Rudolph Research Analytical, Hackettstown, NJ, USA

Measurement Date : Wednesday, 09-FEB-2022

Set Temperature : 20.0

Time Delay : Disabled

Delay between Measurement : Disabled

n	Average	Std.Dev.	% RSD	Maximum	Minimum
5	36.50	0.50	1.36	37.00	36.00

S.No	Sample ID	Time	Result	Scale	OR °Arc	WLG.nm	Lg.mm	Conc.g/100ml	Temp.
1	05R38	05:37:12 PM	36.00	SR	0.072	589	100.00	0.200	20.3
2	05R38	05:37:18 PM	36.00	SR	0.072	589	100.00	0.200	20.2
3	05R38	05:37:25 PM	36.50	SR	0.073	589	100.00	0.200	20.2
4	05R38	05:37:32 PM	37.00	SR	0.074	589	100.00	0.200	20.1
5	05R38	05:37:39 PM	37.00	SR	0.074	589	100.00	0.200	20.1

Figure S11. Specific Rotation of **1** in methanol.

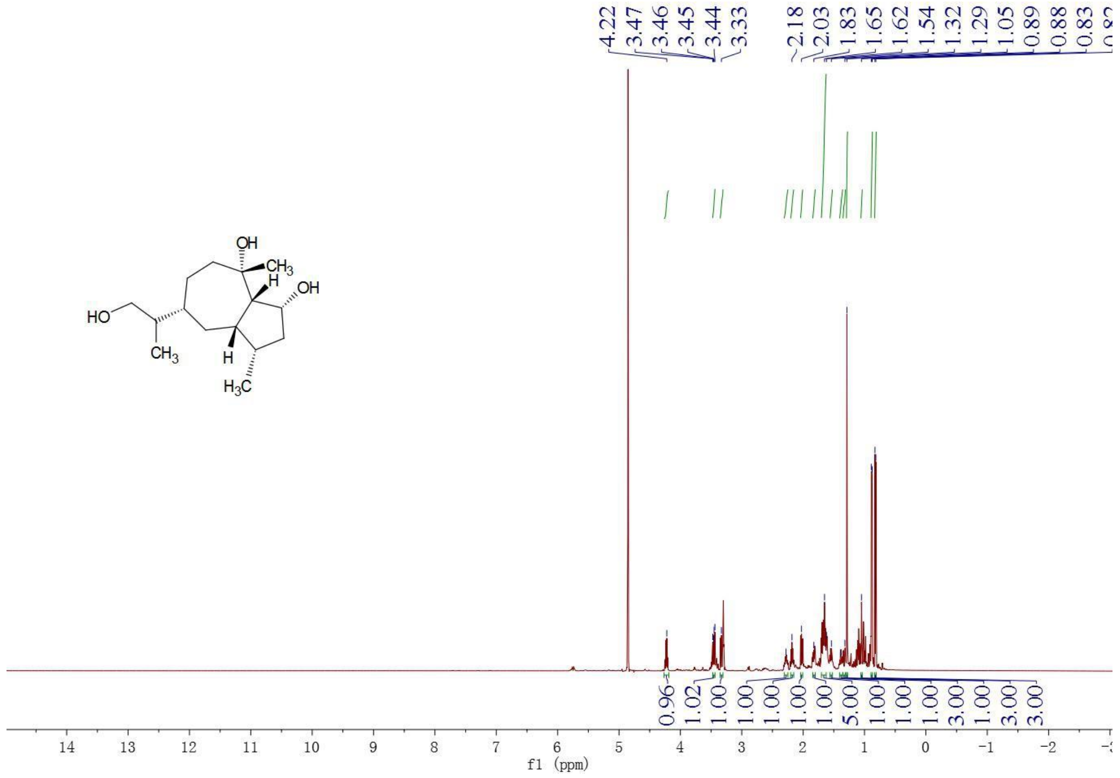

Figure S12. ^{1}H NMR spectrum of compound **2** in CD3OD.

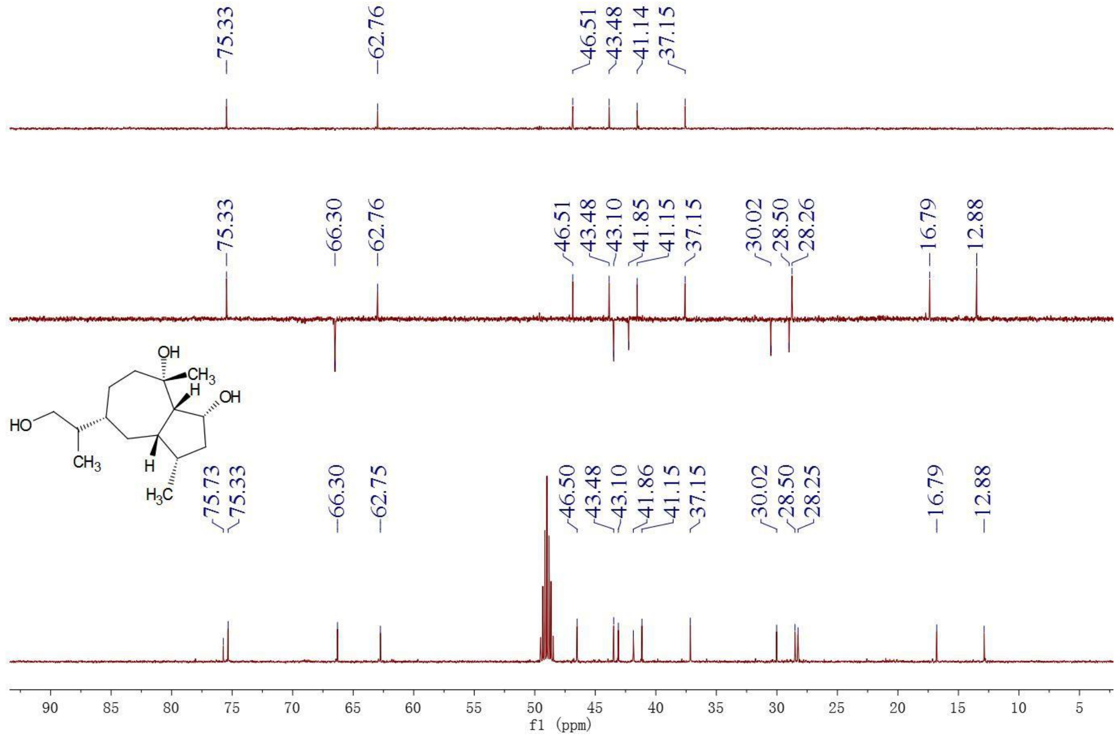

Figure S13. ^{13}C NMR and DEPT spectrum of compound **2** in CD3OD.

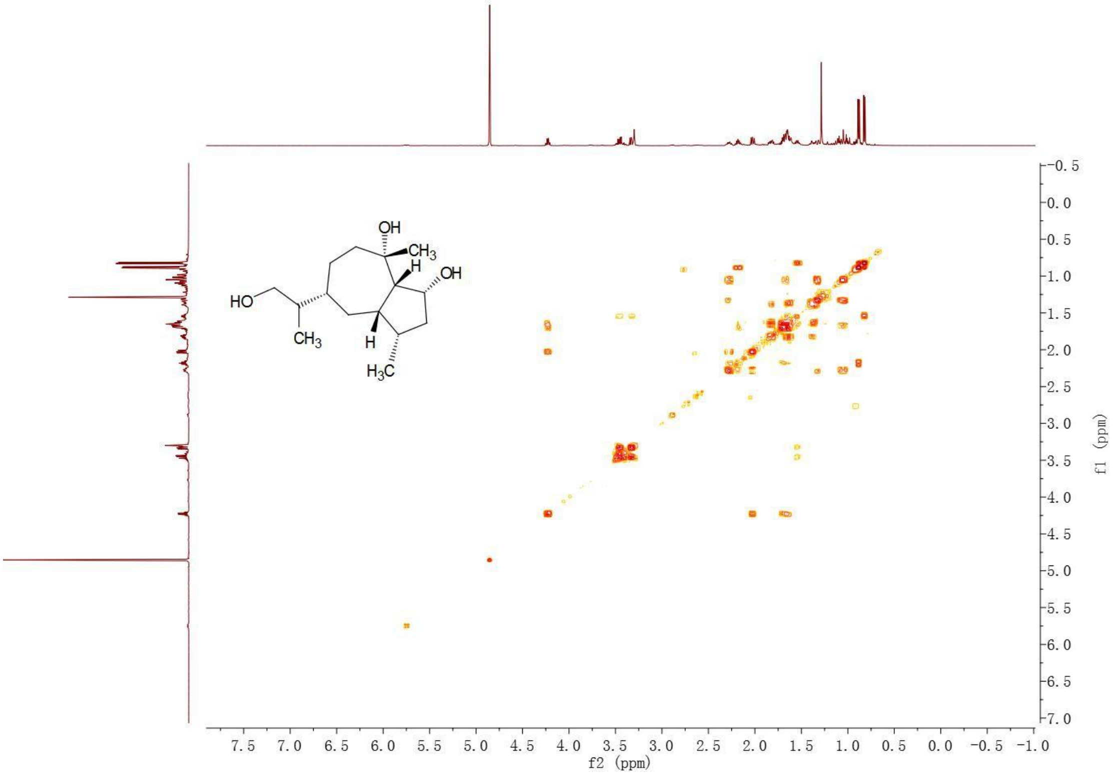

Figure S14. ^{1}H-^{1}H COSY spectrum of compound **2** in CD3OD.

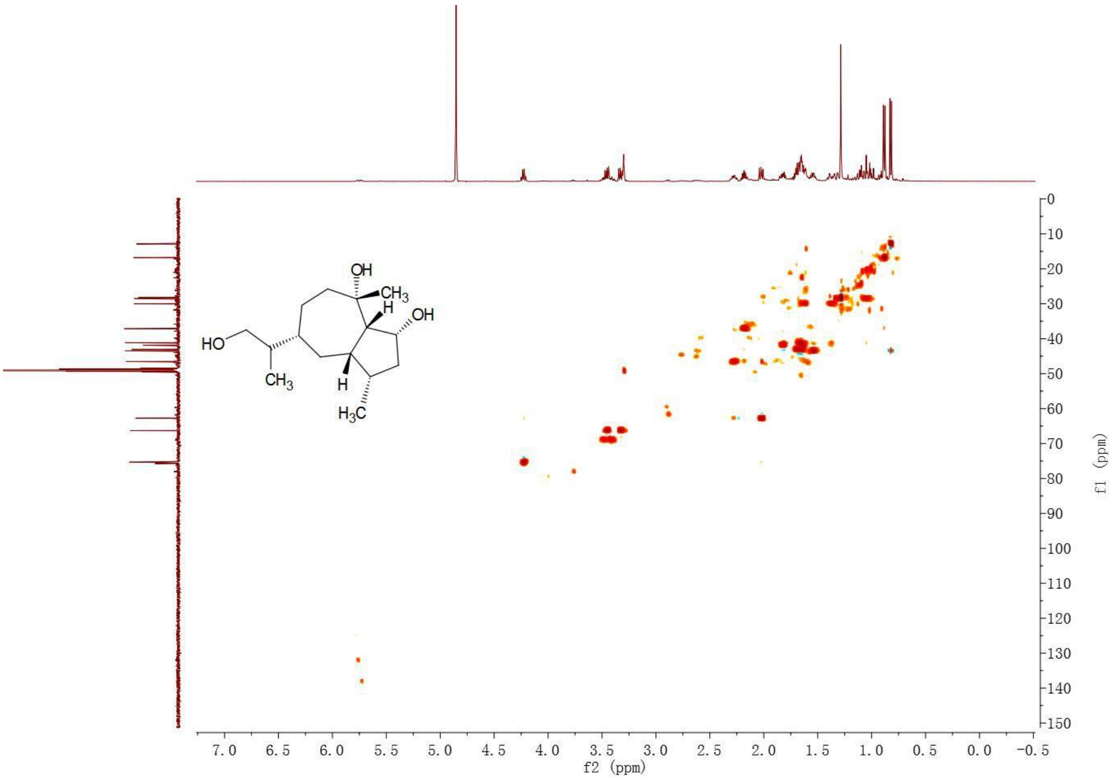

Figure S15. HSQC spectrum of compound **2** in CD3OD.

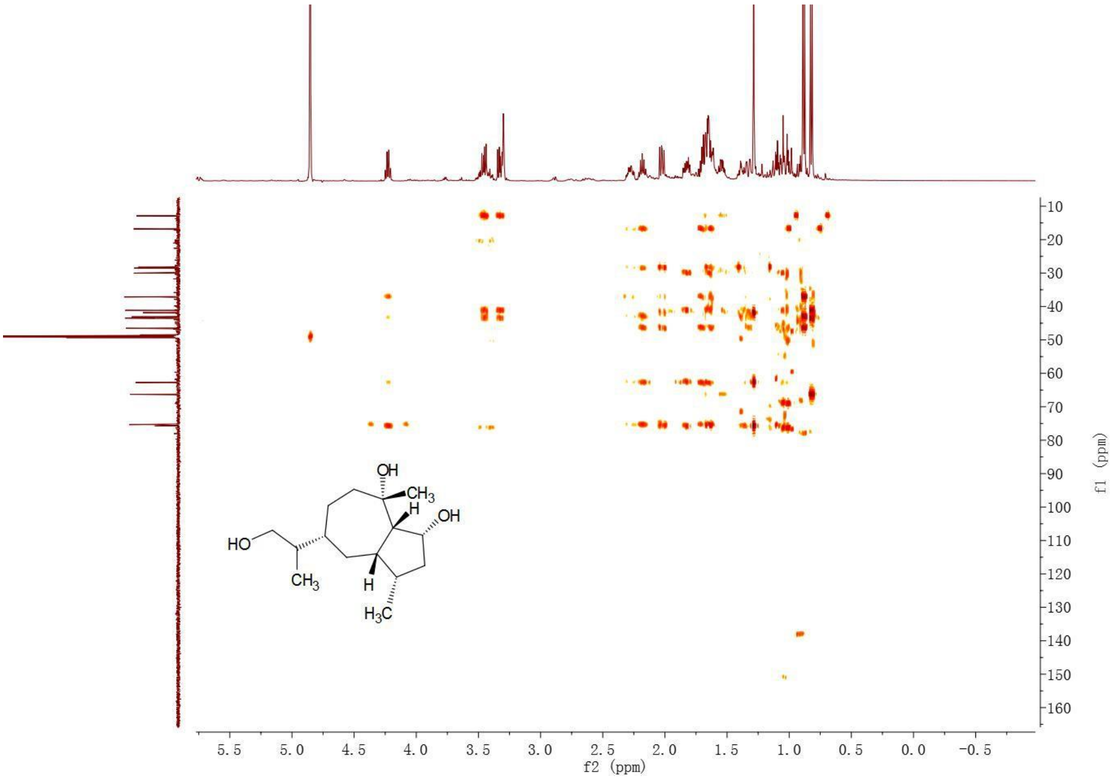

Figure S16. HMBC spectrum of compound **2** in CD3OD.

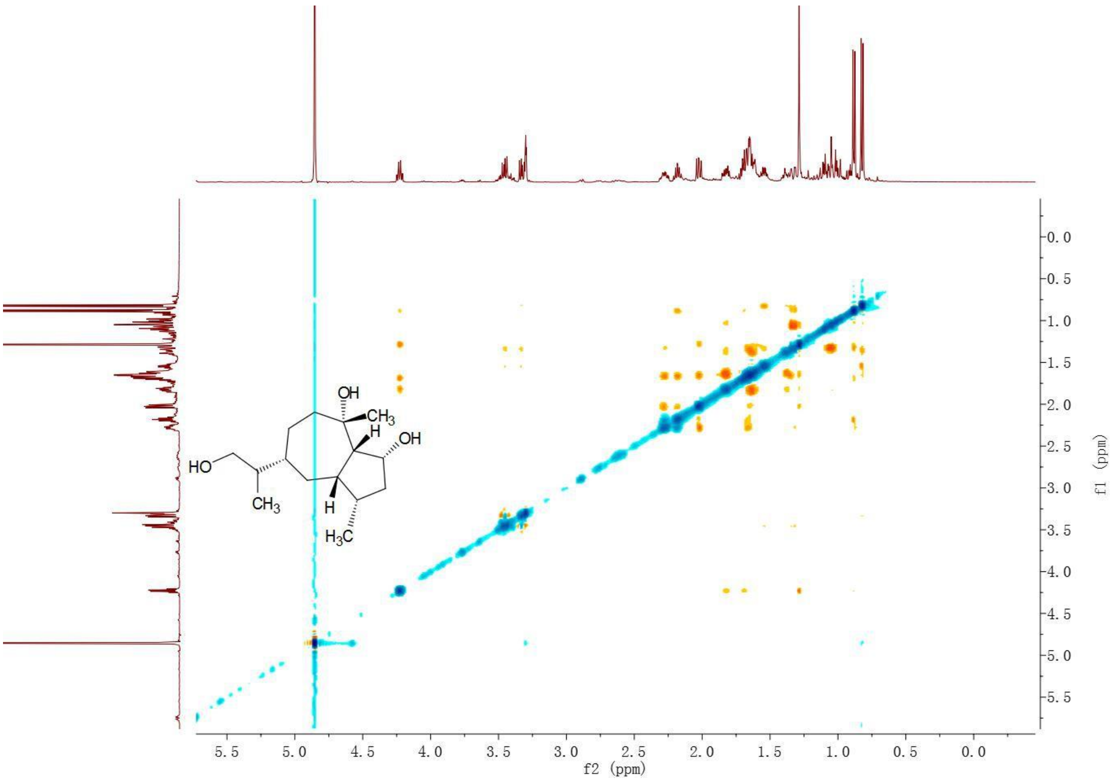

Figure S17. ROESY spectrum of compound **2** in CD3OD.

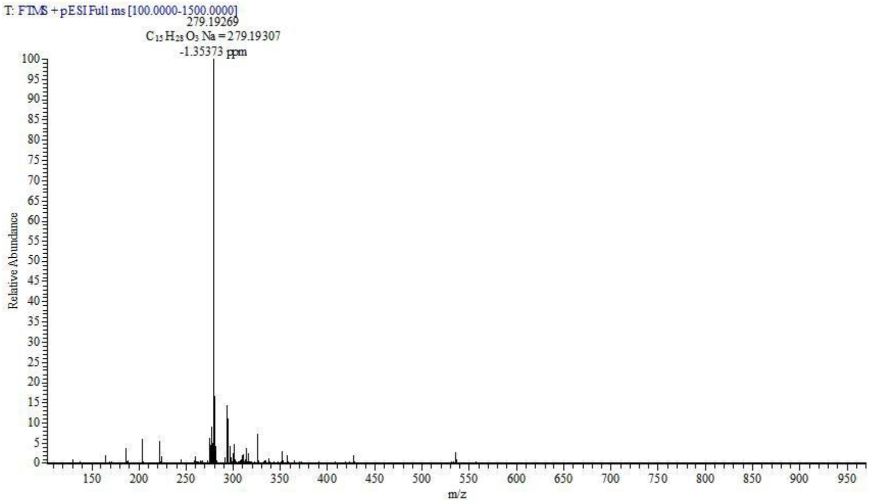

Figure S18. HRESIMS spectrum of compound **2.**

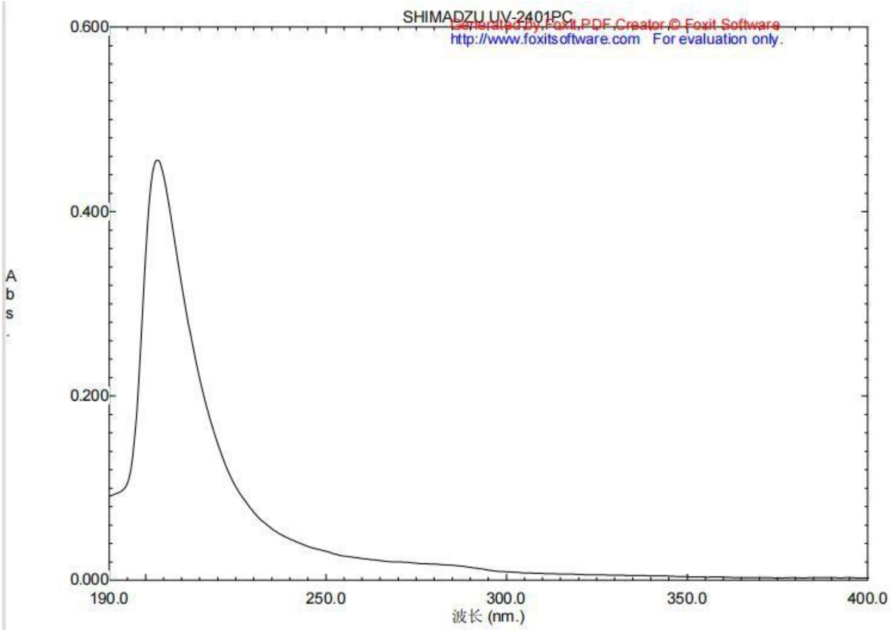

Figure S19. UV spectrum of **2** in methanol.

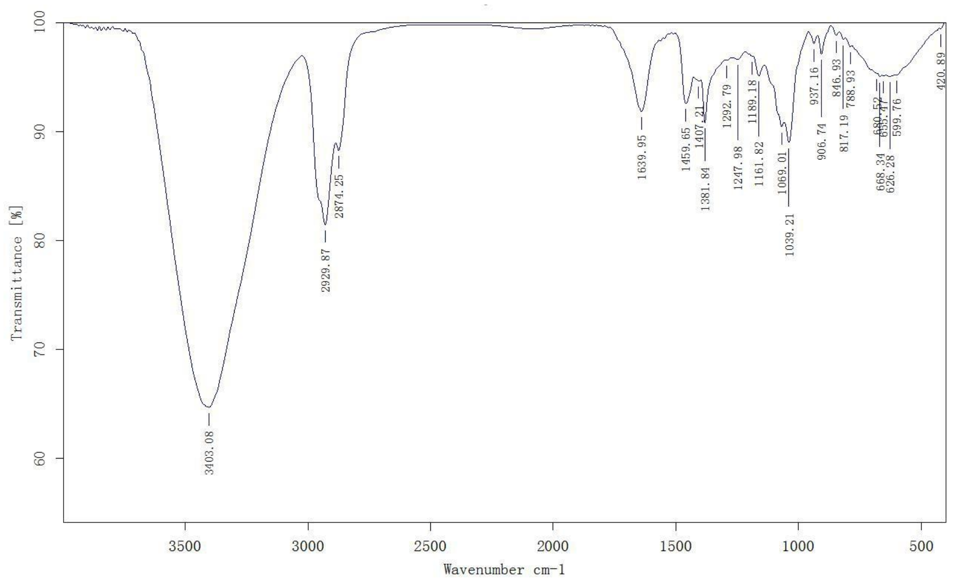

Figure S20. IR spectrum of **2** in methanol.

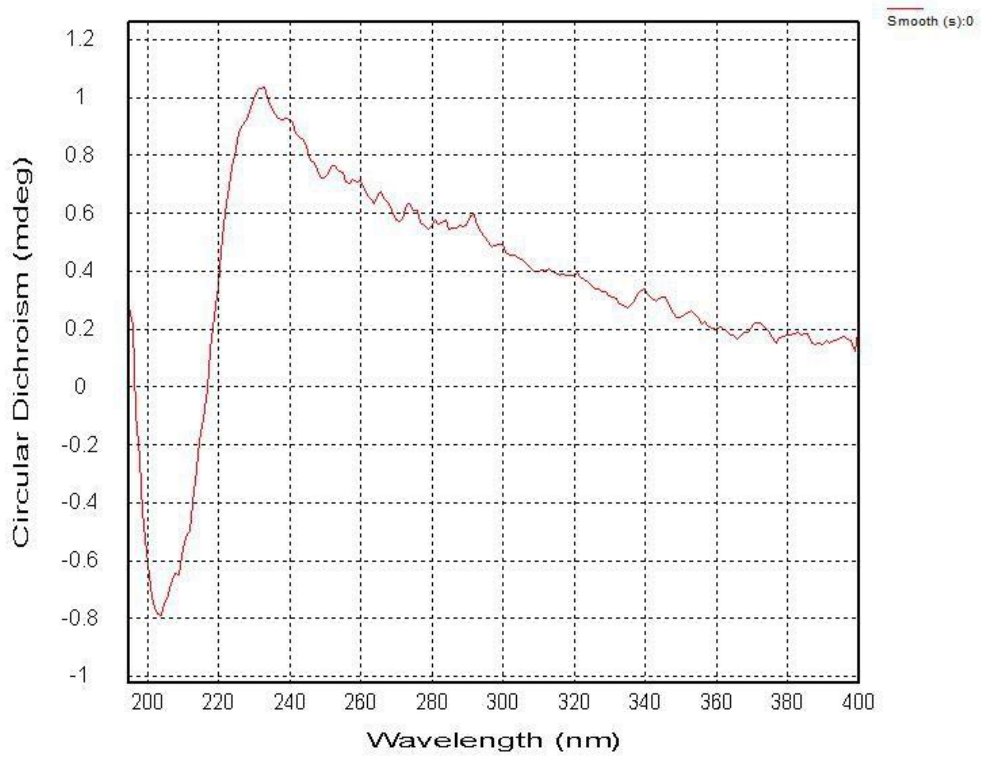

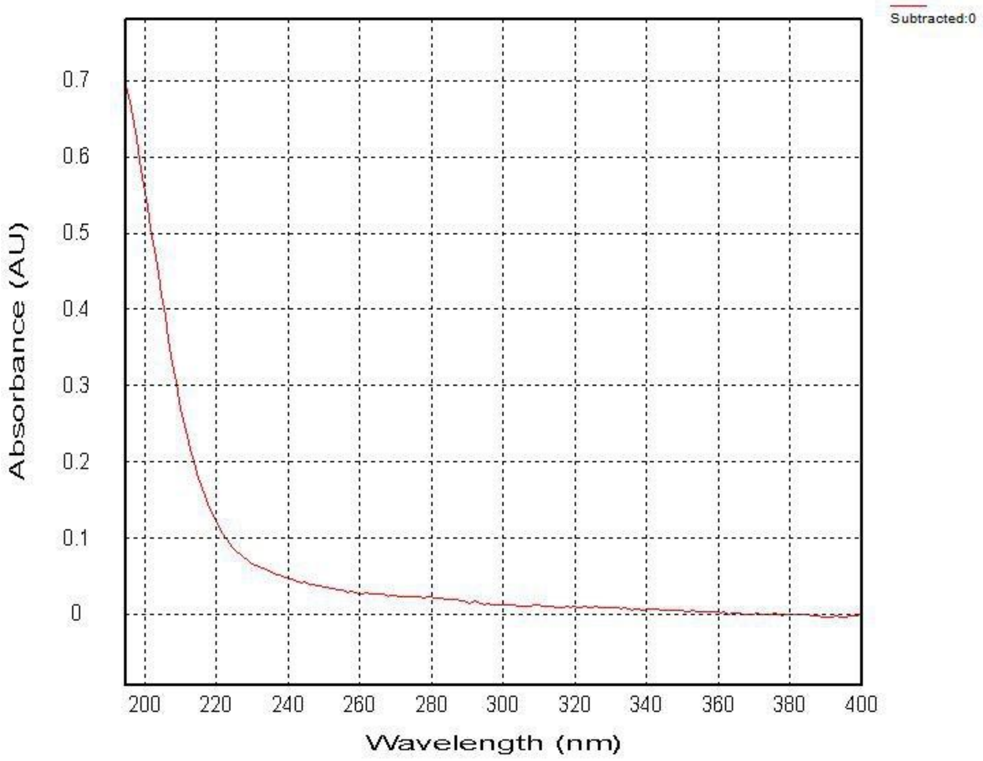

Figure S21. CD spectrum of **2** in methanol.

Rudolph Research Analytical

This sample was measured on an Autopol VI, Serial #91058
Manufactured by Rudolph Research Analytical, Hackettstown, NJ, USA.

Measurement Date : Wednesday, 09-FEB-2022

Set Temperature : 20.0

Time Delay : Disabled

Delay between Measurement : Disabled

n	Average	Std.Dev.	% RSD	Maximum	Minimum
5	-2.33	0.37	-15.87	-1.67	-2.50

S.No	Sample ID	Time	Result	Scale	OR °Arc	WLG.nm	Lg.mm	Conc.g/100ml	Temp.
1	05R47	05:53:55 PM	-1.67	SR	-0.002	589	100.00	0.120	20.1
2	05R47	05:54:02 PM	-2.50	SR	-0.003	589	100.00	0.120	20.1
3	05R47	05:54:08 PM	-2.50	SR	-0.003	589	100.00	0.120	20.1
4	05R47	05:54:15 PM	-2.50	SR	-0.003	589	100.00	0.120	20.0
5	05R47	05:54:22 PM	-2.50	SR	-0.003	589	100.00	0.120	20.0

Figure S22. Specific Rotation of **2** in methanol.

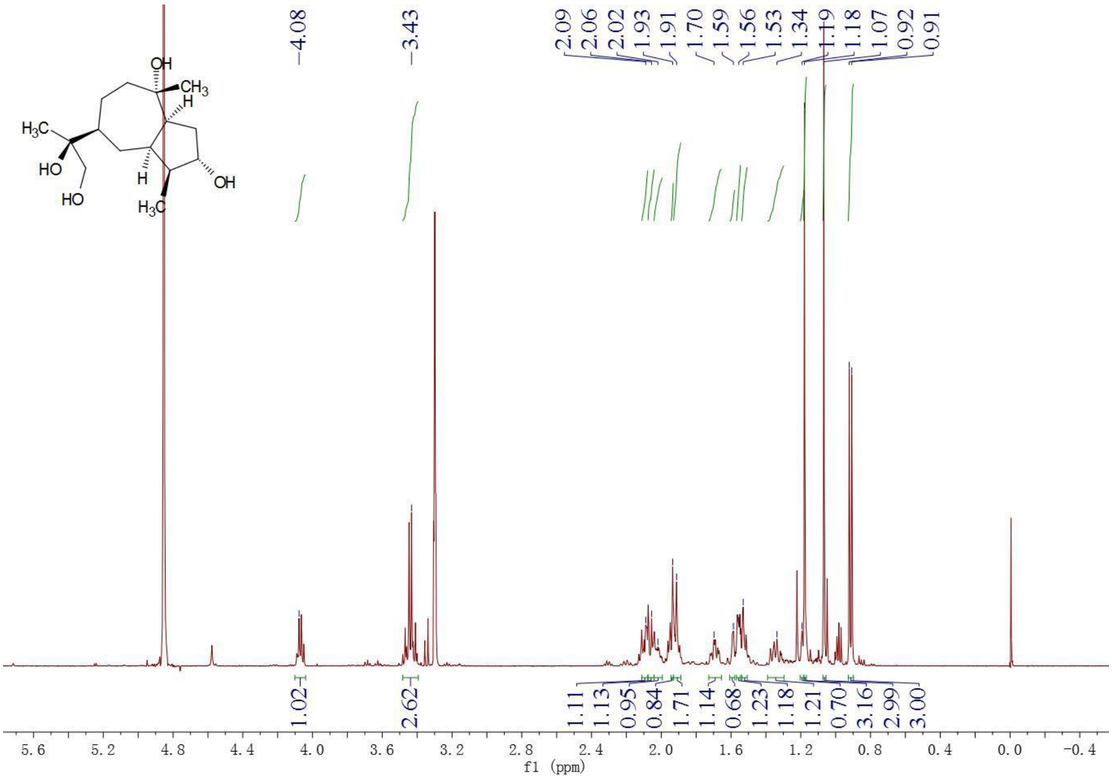

Figure S23. ^{1}H NMR spectrum of compound **3** in CD3OD.

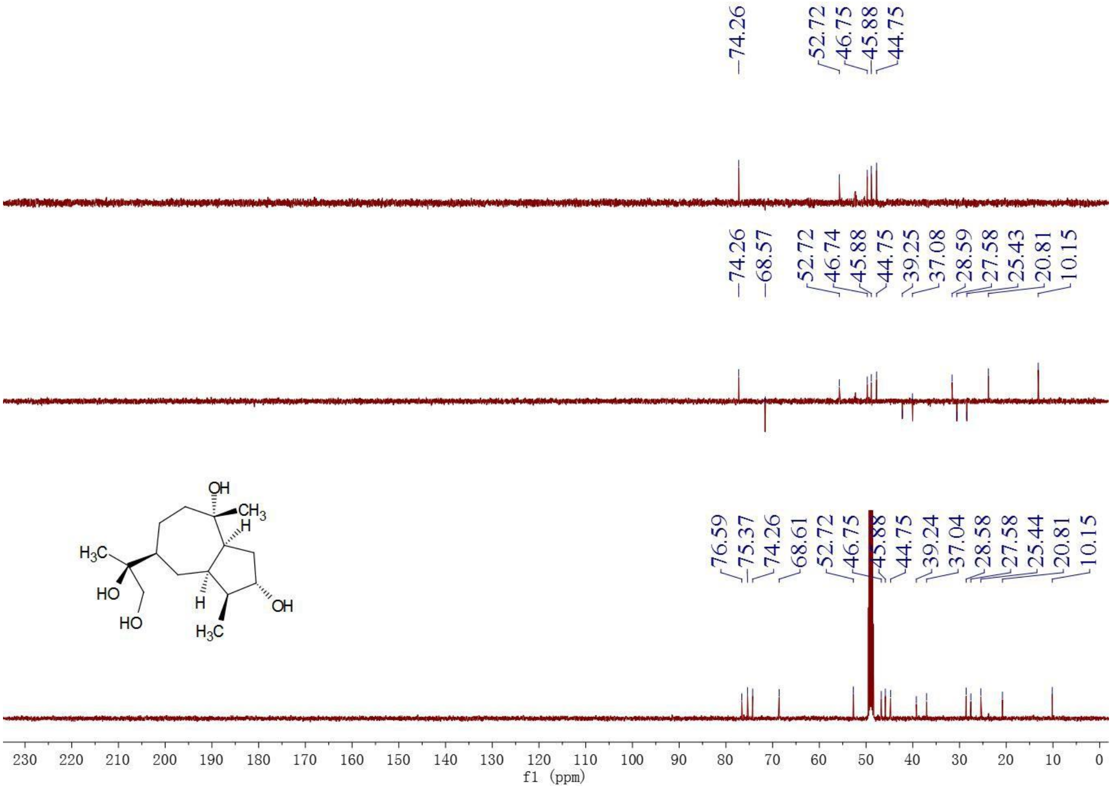

Figure S24. ^{13}C NMR and DEPT spectrum of compound **3** in CD3OD.

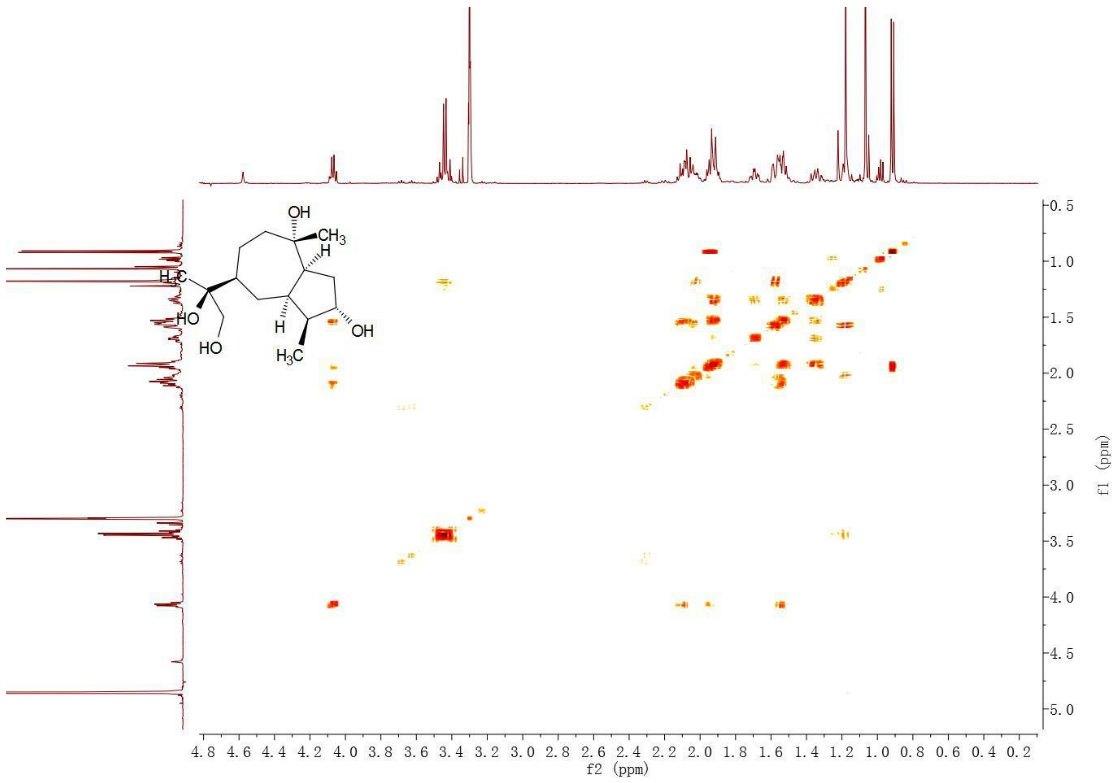

Figure S25. ^{1}H-^{1}H COSY spectrum of compound **3** in CD3OD.

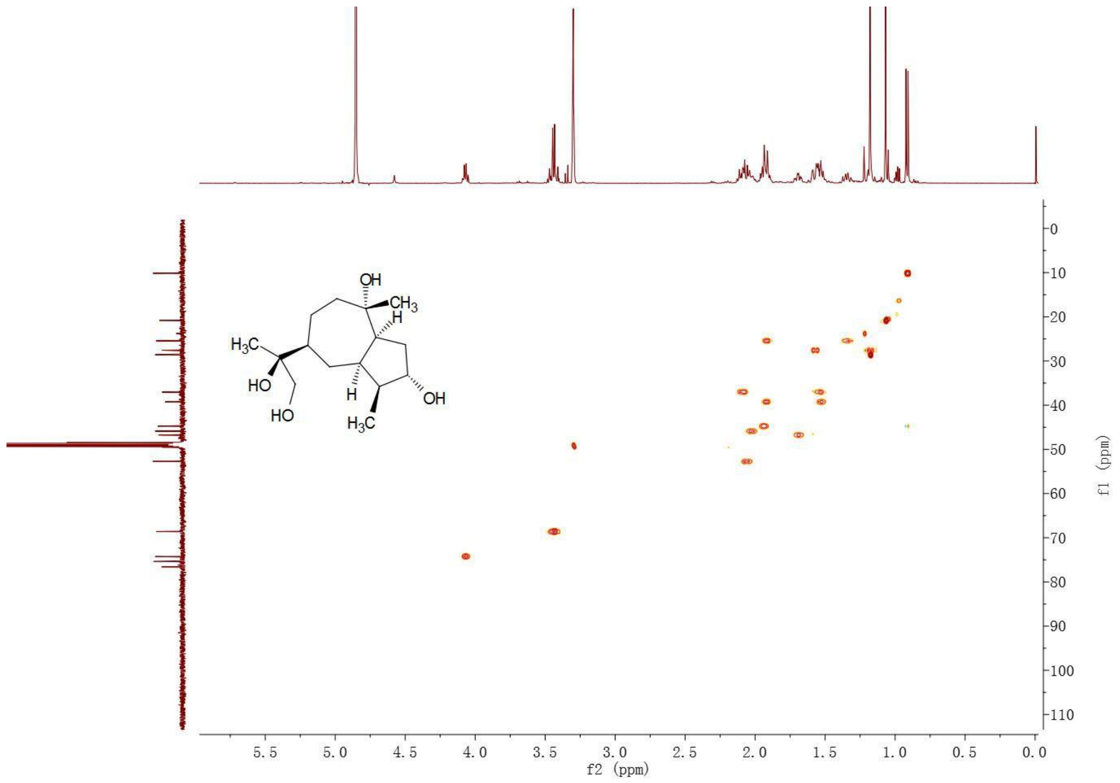

Figure S26. HSQC spectrum of compound **3** in CD3OD.

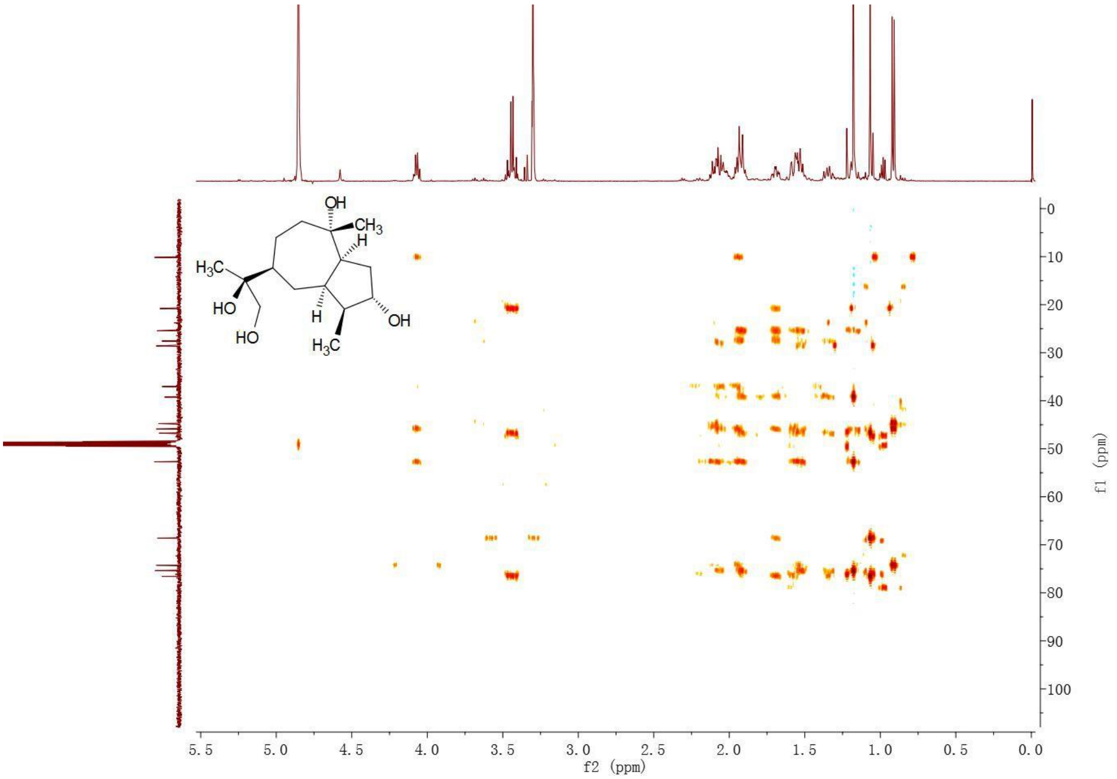

Figure S27. HMBC spectrum of compound **3** in CD3OD.

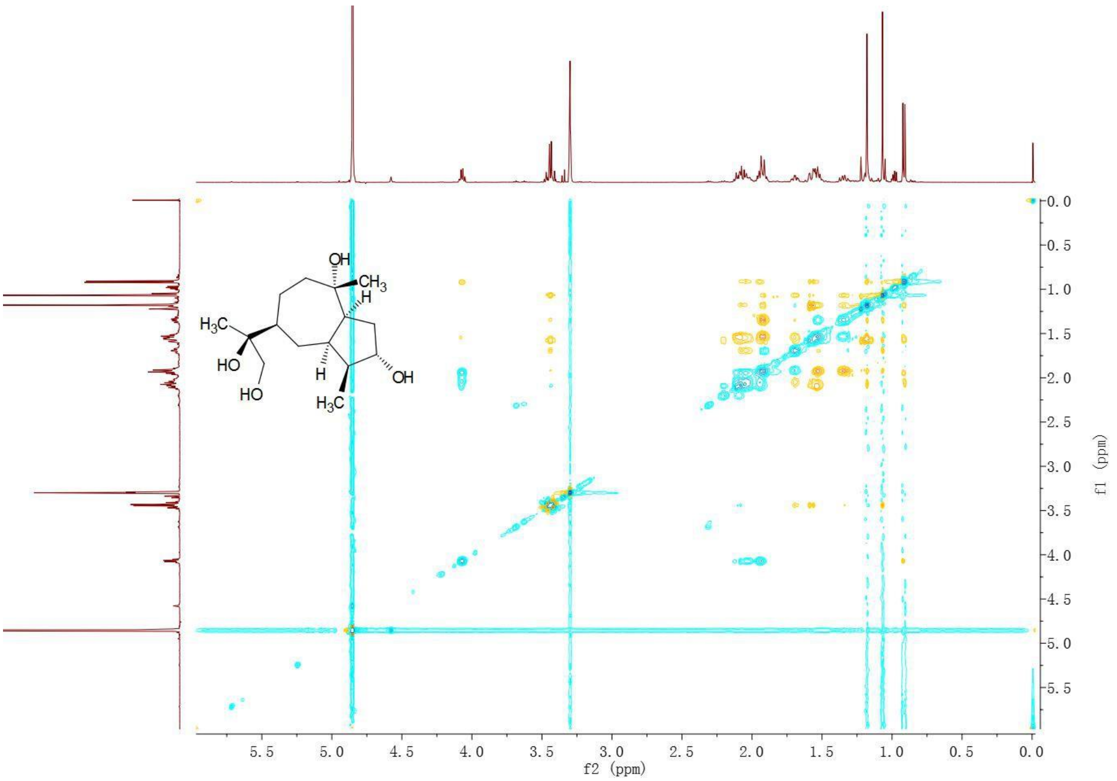

Figure S28. ROESY spectrum of compound **3** in CD3OD.

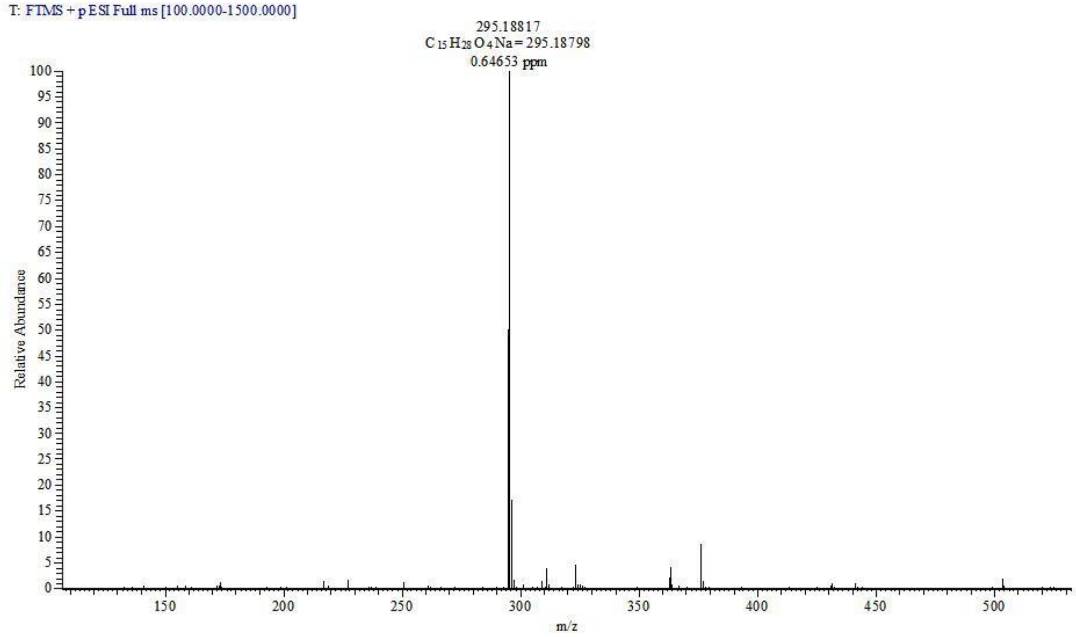

Figure S29. HRESIMS spectrum of compound **3.**

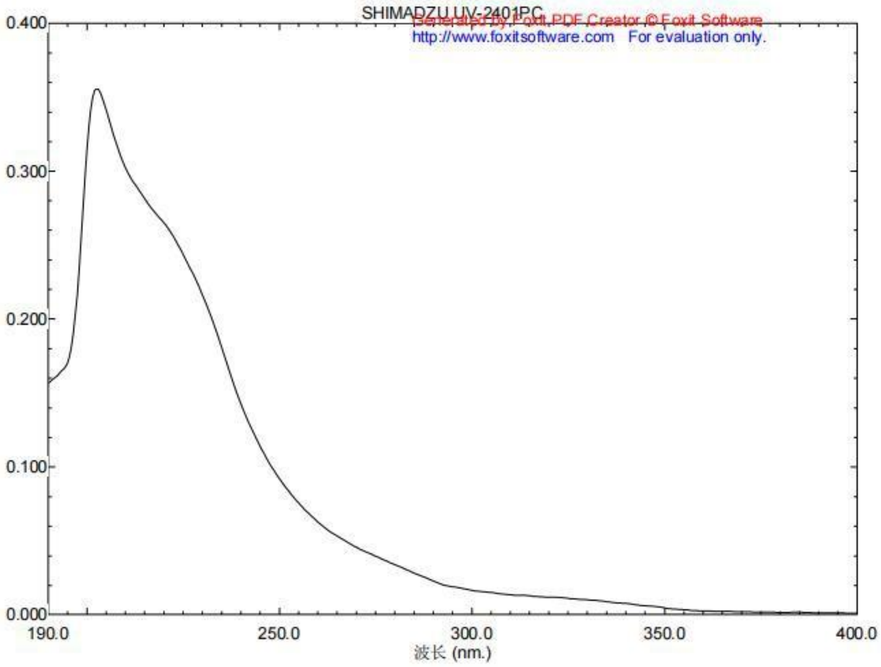

Figure S30. UV spectrum of **3** in methanol.

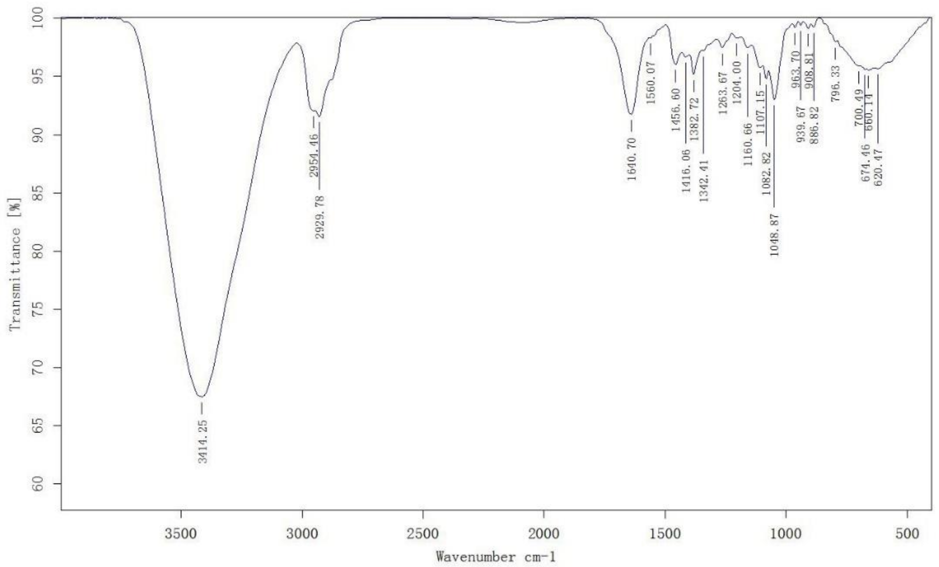

Figure S31. IR spectrum of **3** in methanol.

Rudolph Research Analytical

This sample was measured on an Autopol VI, Serial #91058
Manufactured by Rudolph Research Analytical, Hackettstown, NJ, USA.

Measurement Date : Saturday, 28-MAY-2022

Set Temperature : OFF

Time Delay : Disabled

Delay between Measurement : Disabled

n	Average	Std.Dev.	% RSD	Maximum	Minimum
5	-10.78	0.26	-2.41	-10.40	-11.10

S.No	Sample ID	Time	Result	Scale	OR °Arc	WLG.nm	Lg.mm	Conc.g/100ml	Temp.
1	05R14	11:10:52 AM	-10.90	SR	-0.0109	589	100.00	0.100	24.4
2	05R14	11:11:01 AM	-10.40	SR	-0.0104	589	100.00	0.100	24.4
3	05R14	11:11:09 AM	-10.80	SR	-0.0108	589	100.00	0.100	24.4
4	05R14	11:11:17 AM	-11.10	SR	-0.0111	589	100.00	0.100	24.4
5	05R14	11:11:25 AM	-10.70	SR	-0.0107	589	100.00	0.100	24.4

Figure S32. Specific Rotation of **3** in methanol.

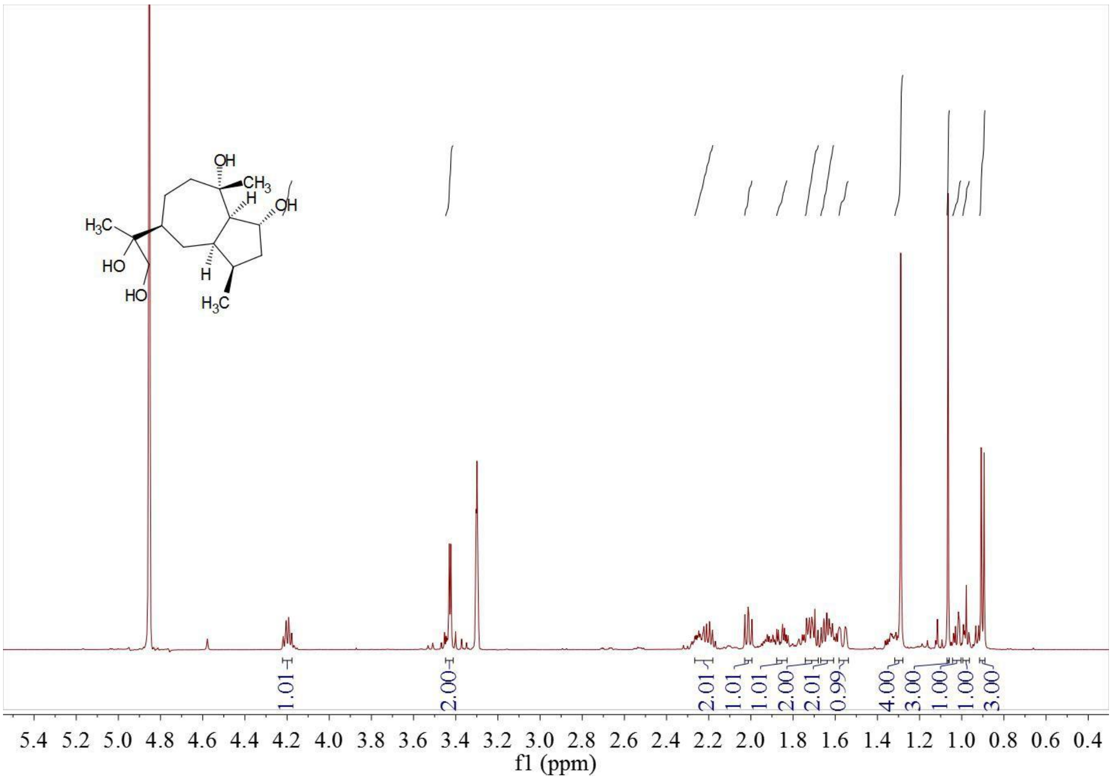

Figure S33. ^{1}H NMR spectrum of compound **4** in CD3OD.

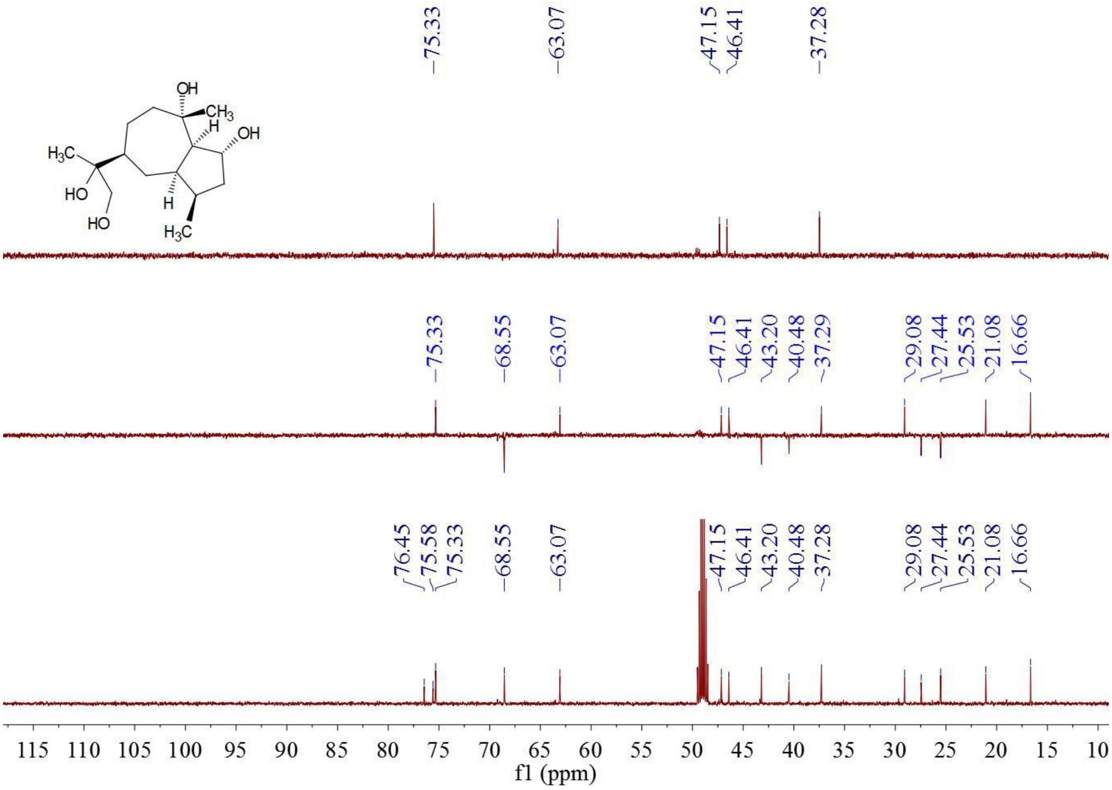

Figure S34. ^{13}C NMR and DEPT spectrum of compound **4** in CD3OD.

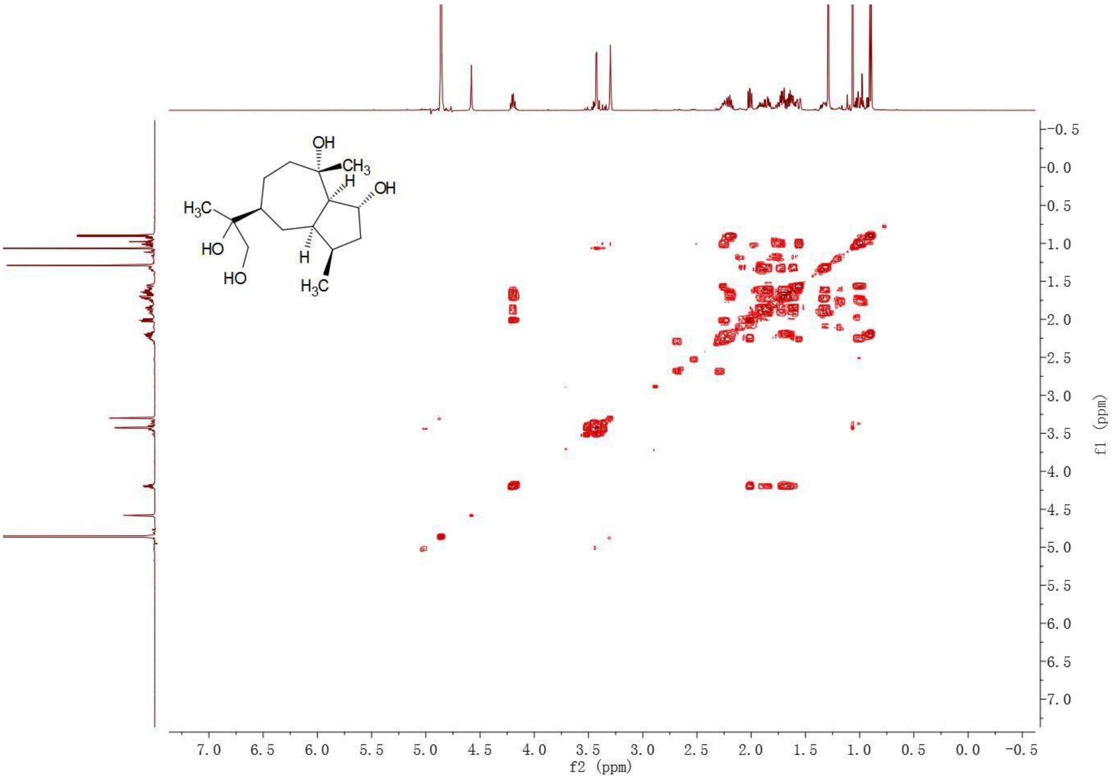

Figure S35. ^{1}H-^{1}H COSY spectrum of compound **4** in CD3OD.

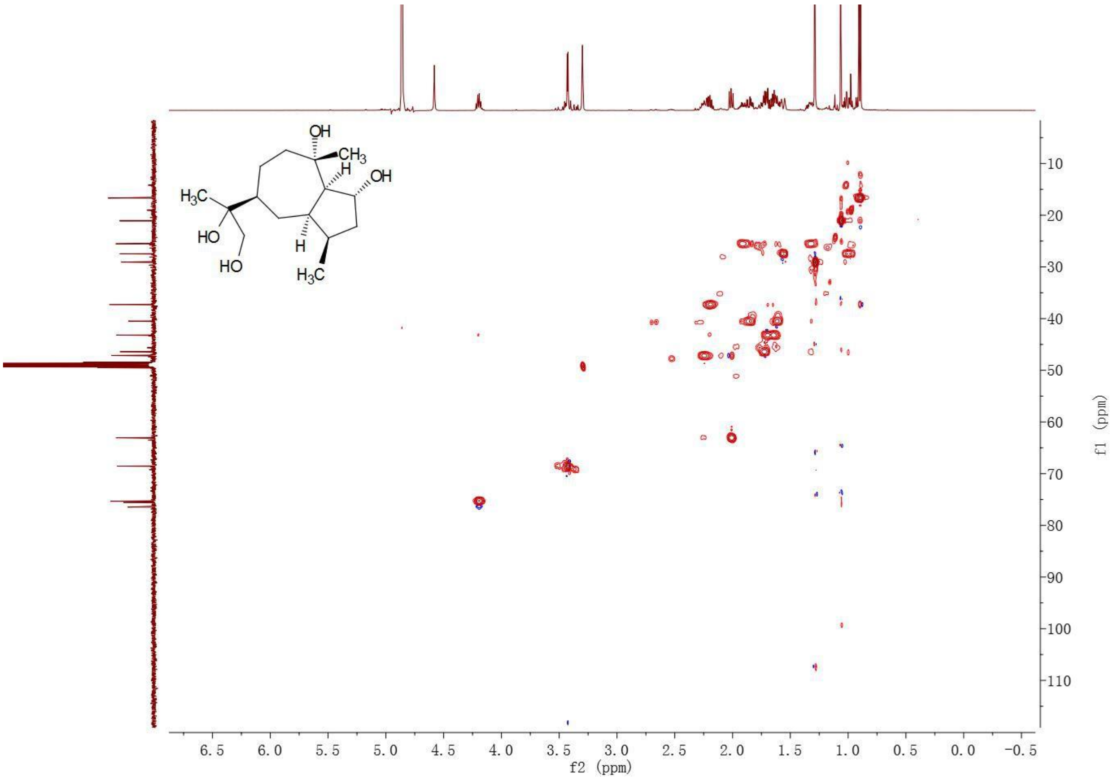

Figure S36. HSQC spectrum of compound **4** in CD3OD.

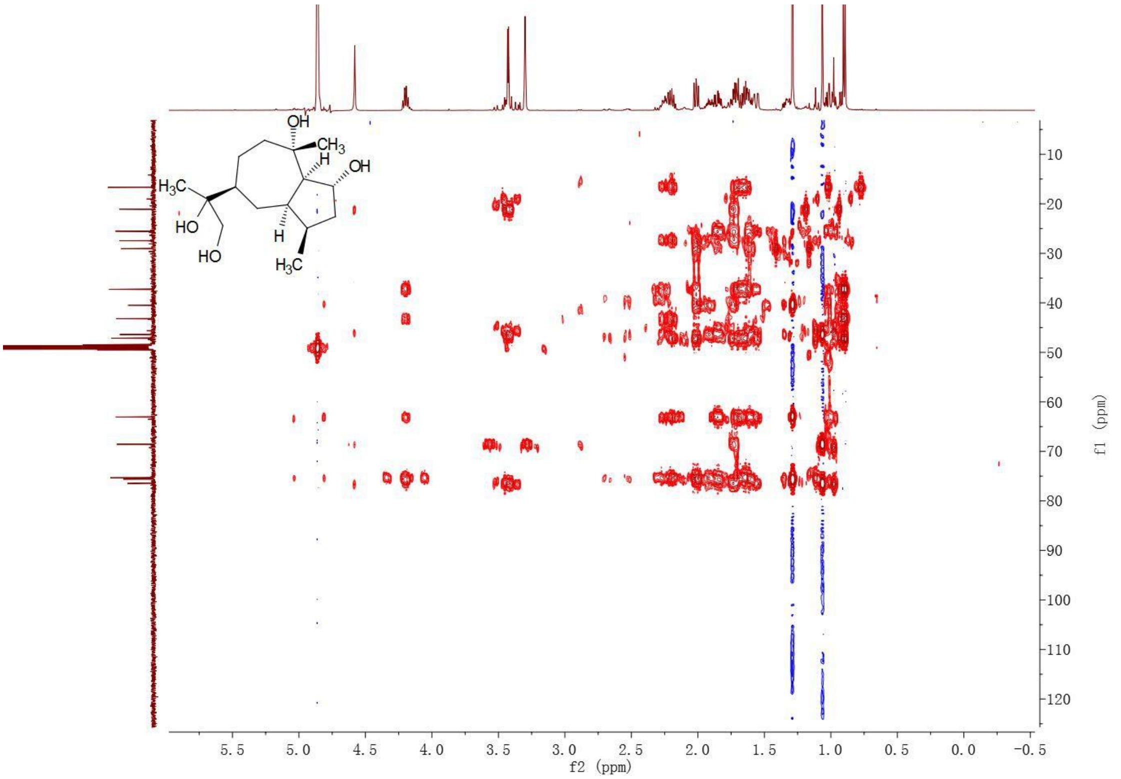

Figure S37. HMBC spectrum of compound **4** in CD3OD.

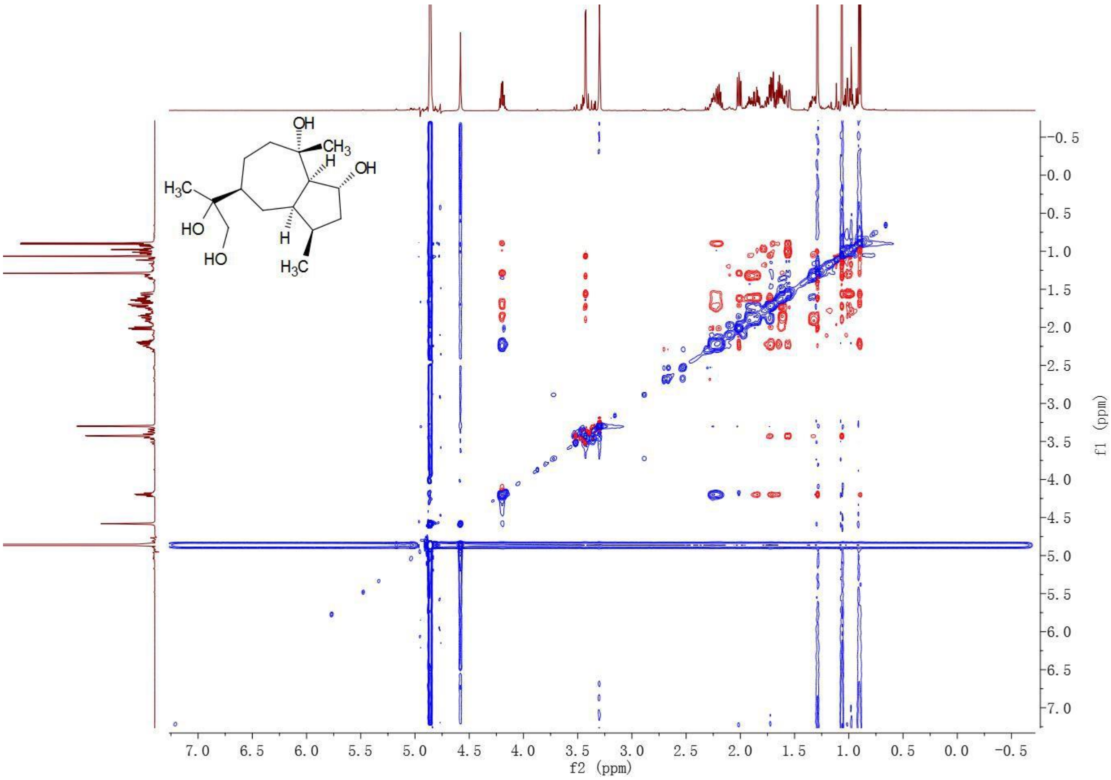

Figure S38. ROESY spectrum of compound **4** in CD3OD.

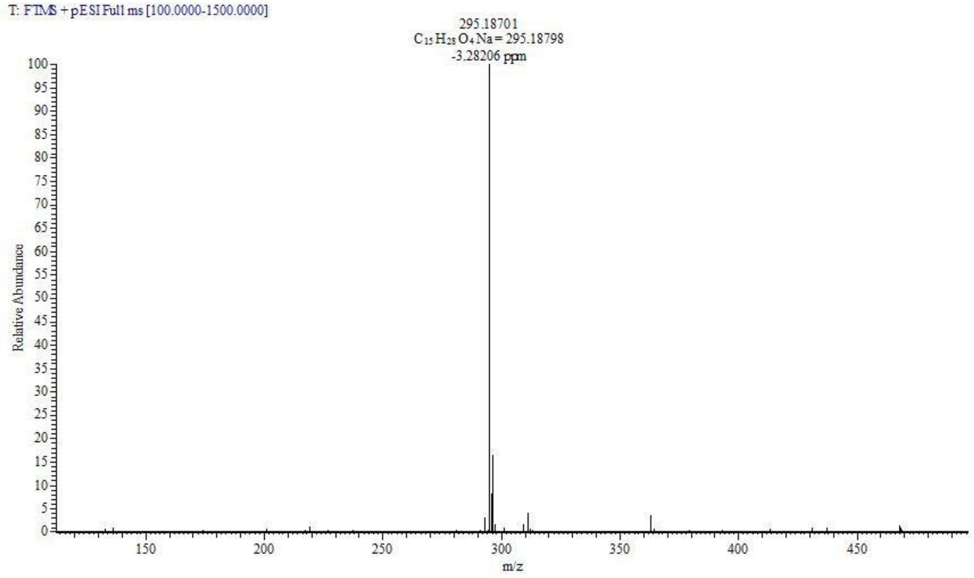

Figure S39. HRESIMS spectrum of compound **4.**

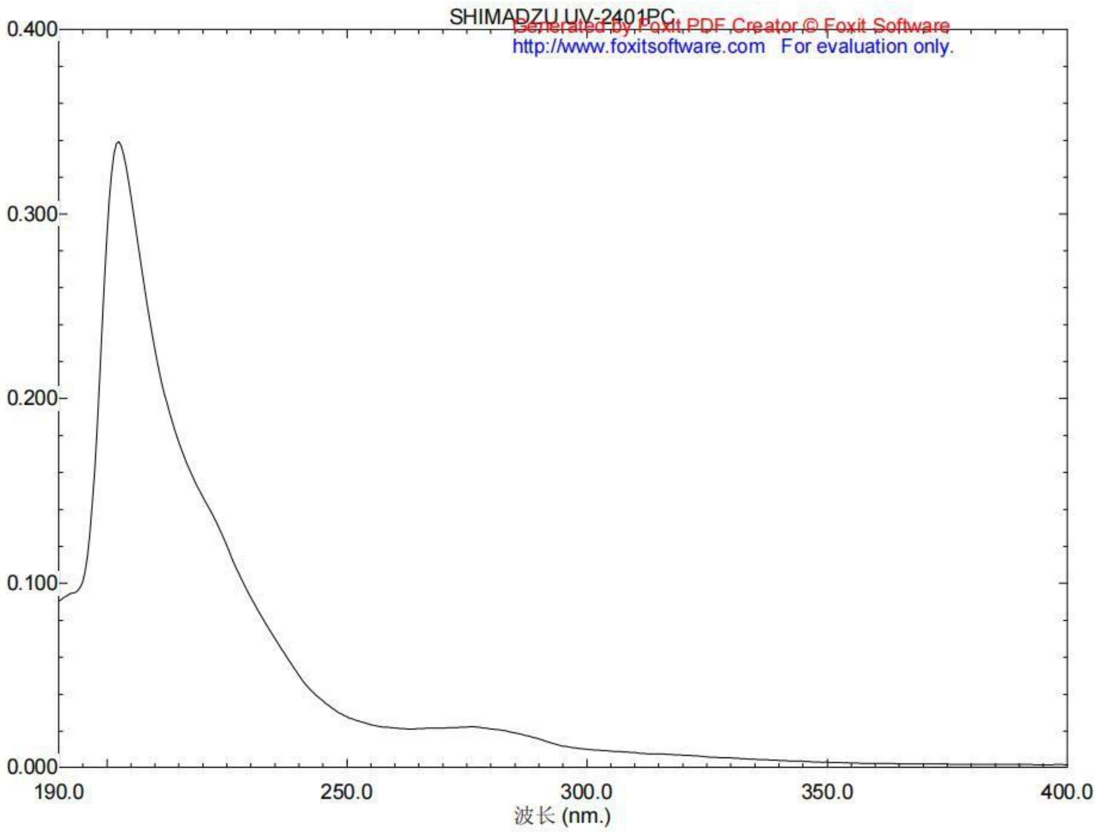

Figure S40. UV spectrum of **4** in methanol.

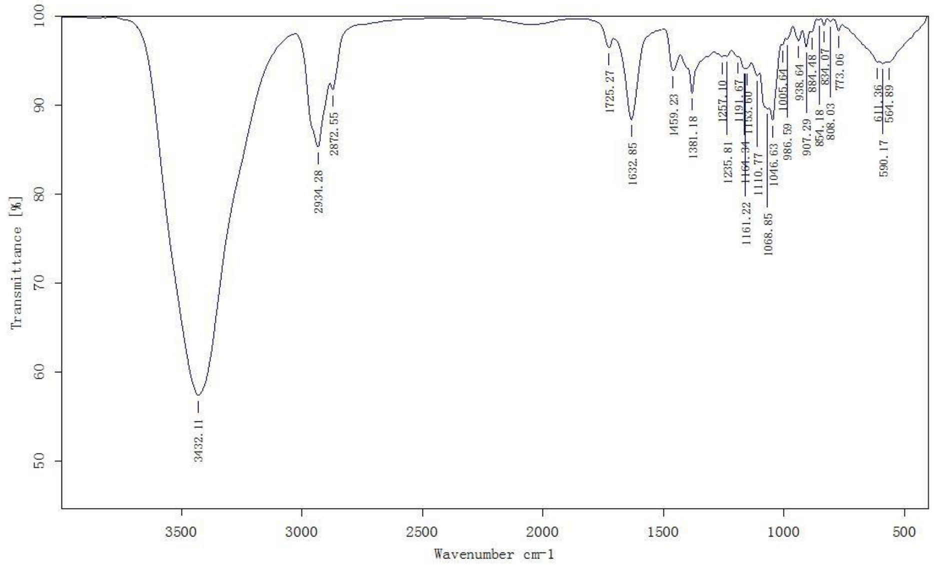

Figure S41. IR spectrum of **4** in methanol.

Rudolph Research Analytical

This sample was measured on an Autopol VI, Serial #91058
Manufactured by Rudolph Research Analytical, Hackettstown, NJ, USA.

Measurement Date : Tuesday, 30-AUG-2022

Set Temperature : OFF

Time Delay : Disabled

Delay between Measurement : Disabled

n	Average	Std.Dev.	% RSD	Maximum	Minimum
5	-1.88	0.64	-34.04	-0.75	-2.33

S.No	Sample ID	Time	Result	Scale	OR °Arc	WLG.nm	Lg.mm	Conc.g/100ml	Temp.
1	05R49	10:50:31 AM	-0.75	SR	-0.0009	589	100.00	0.120	24.7
2	05R49	10:50:40 AM	-2.08	SR	-0.0025	589	100.00	0.120	24.7
3	05R49	10:50:52 AM	-2.17	SR	-0.0026	589	100.00	0.120	24.6
4	05R49	10:51:00 AM	-2.33	SR	-0.0028	589	100.00	0.120	24.6
5	05R49	10:51:09 AM	-2.08	SR	-0.0025	589	100.00	0.120	24.6

Figure S42. Specific Rotation of **4** in methanol.

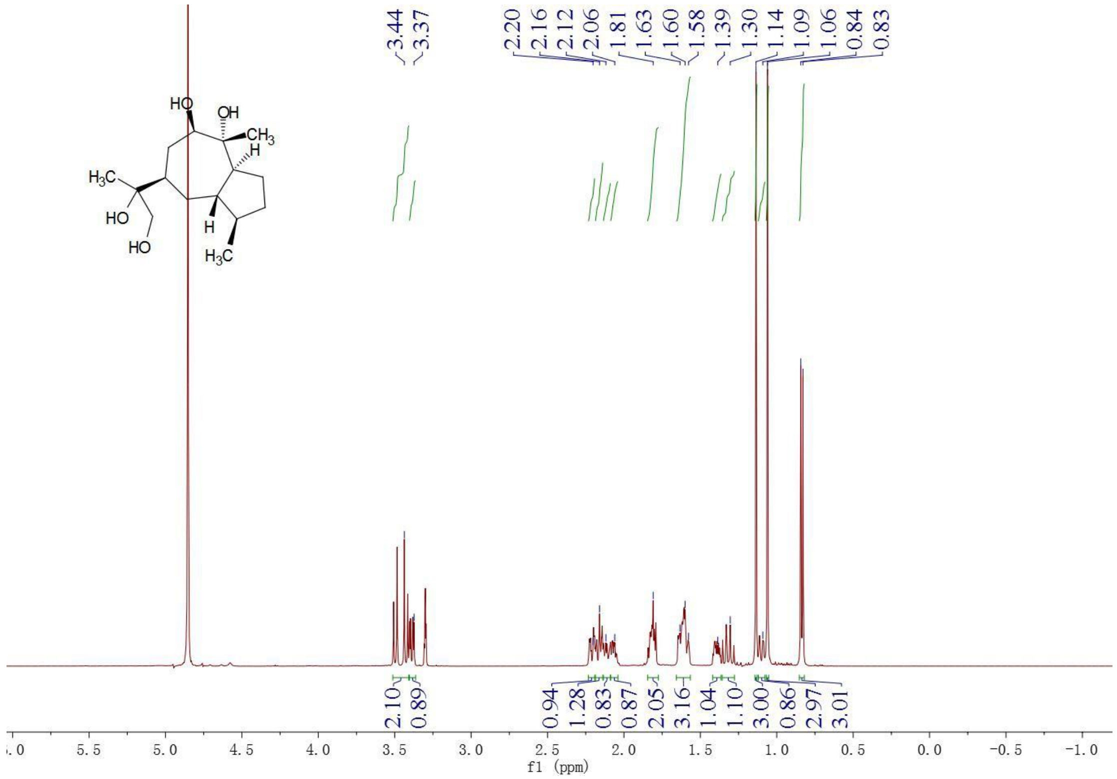

Figure S43. ^{1}H NMR spectrum of compound **5** in CD3OD.

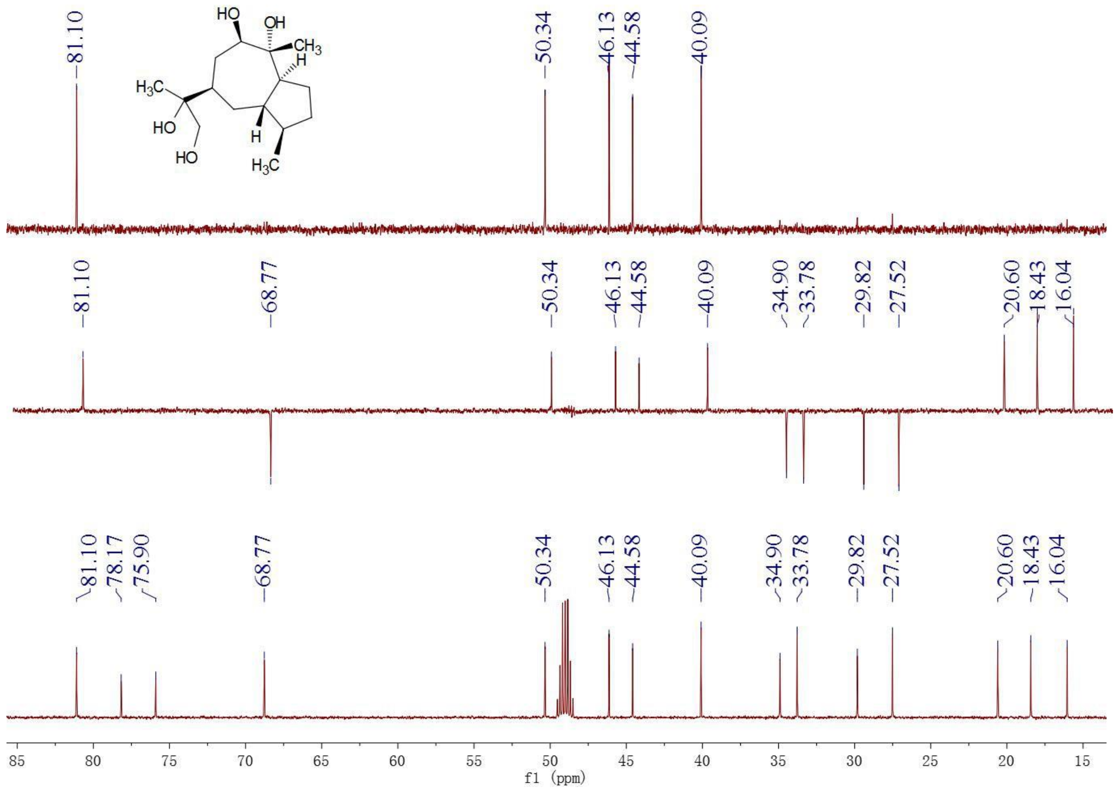

Figure S44. ^{13}C NMR and DEPT spectrum of compound **5** in CD3OD.

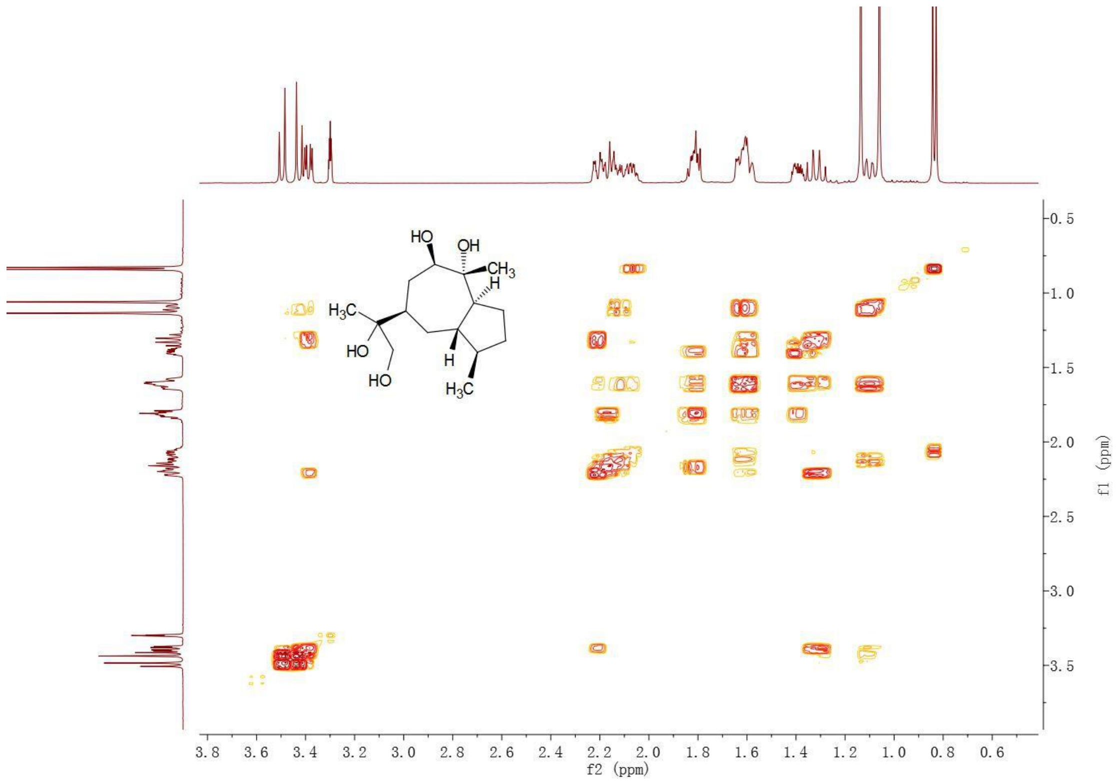

Figure S45. ^{1}H-^{1}H COSY spectrum of compound **5** in CD3OD.

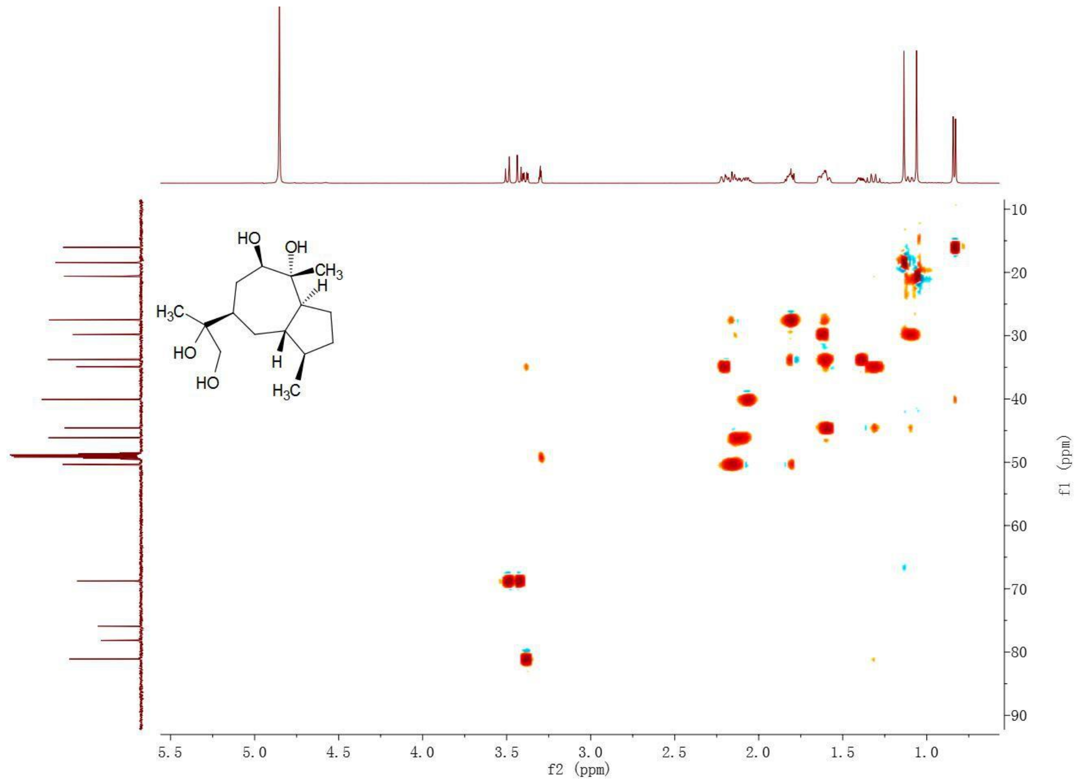

Figure S46. HSQC spectrum of compound **5** in CD3OD.

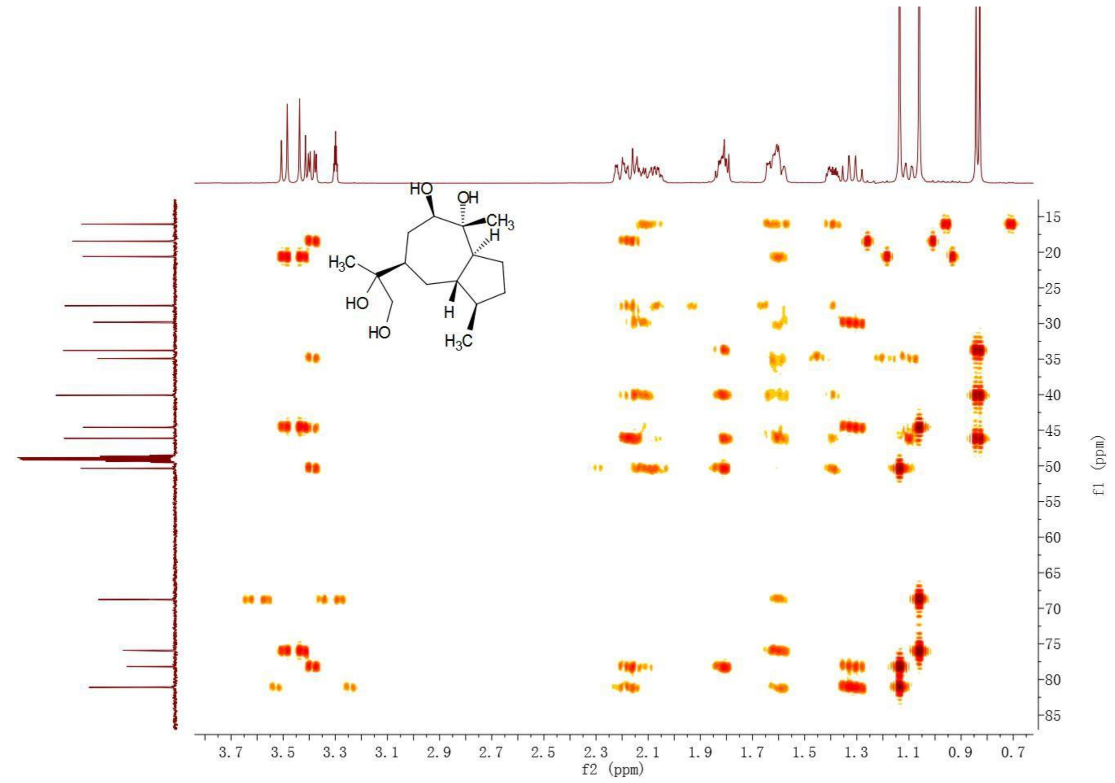

Figure S47. HMBC spectrum of compound **5** in CD3OD.

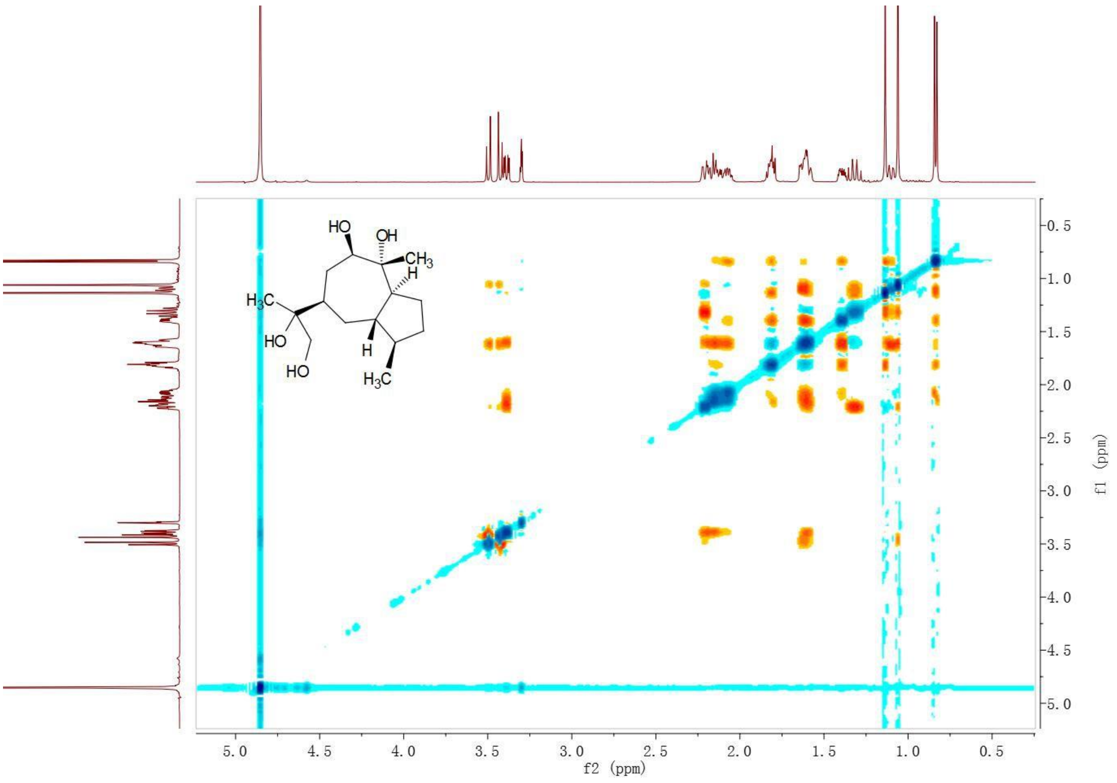

Figure S48. ROESY spectrum of compound **5** in CD3OD.

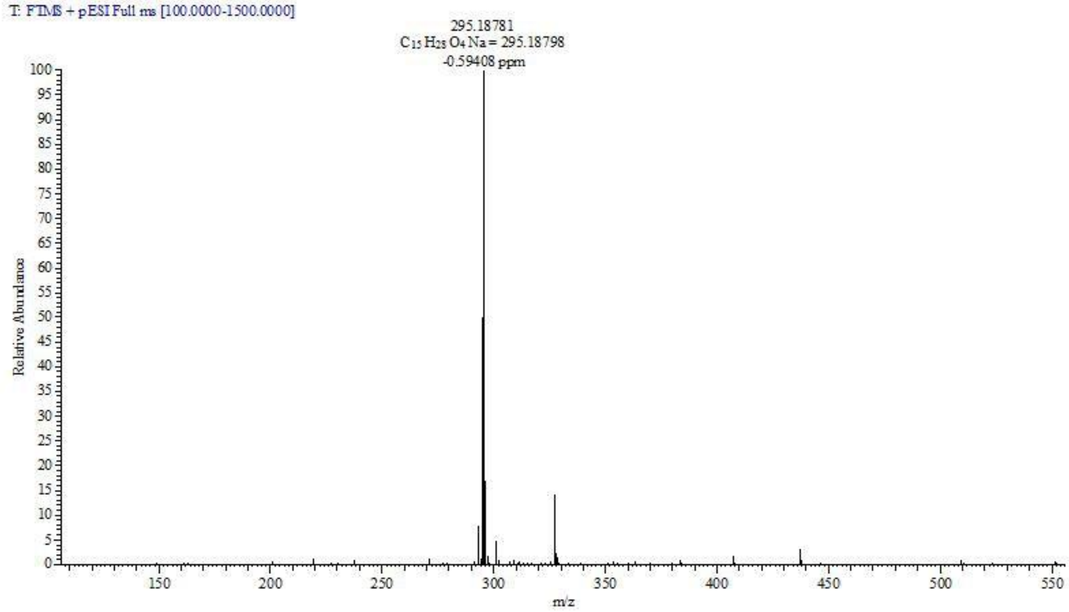

Figure S49. HRESIMS spectrum of compound **5.**

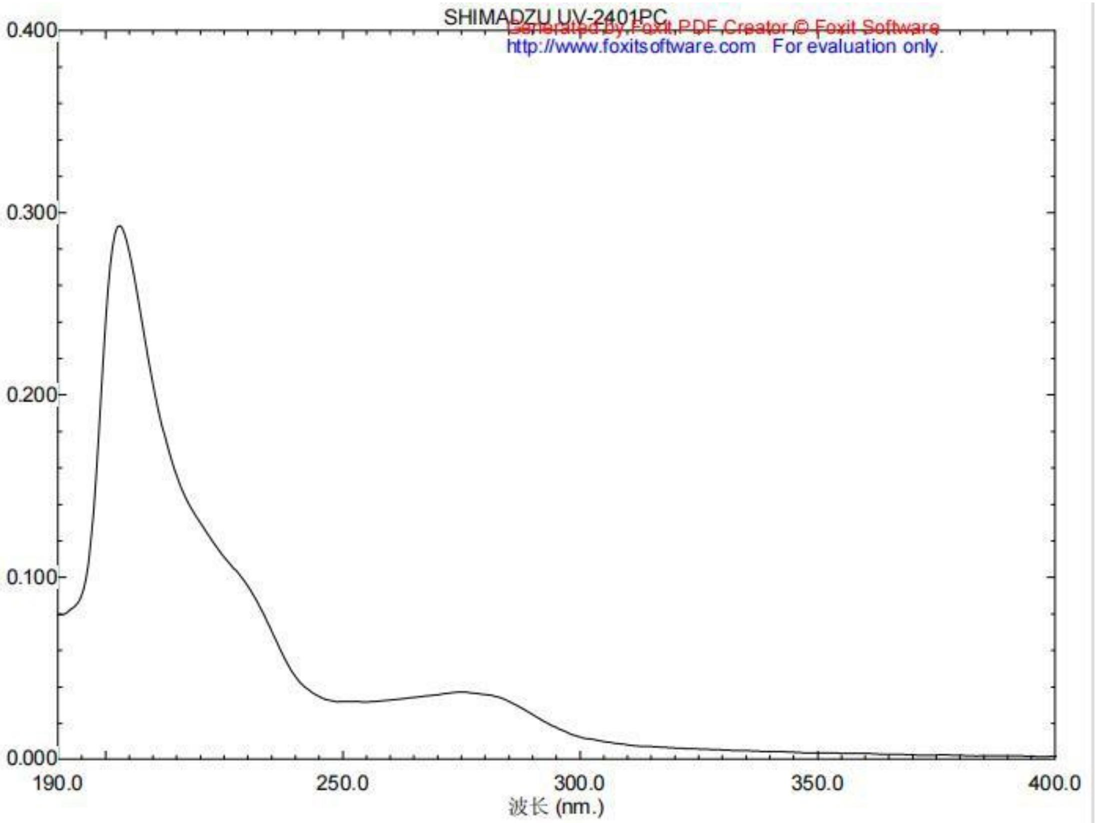

Figure S50. UV spectrum of **5** in methanol.

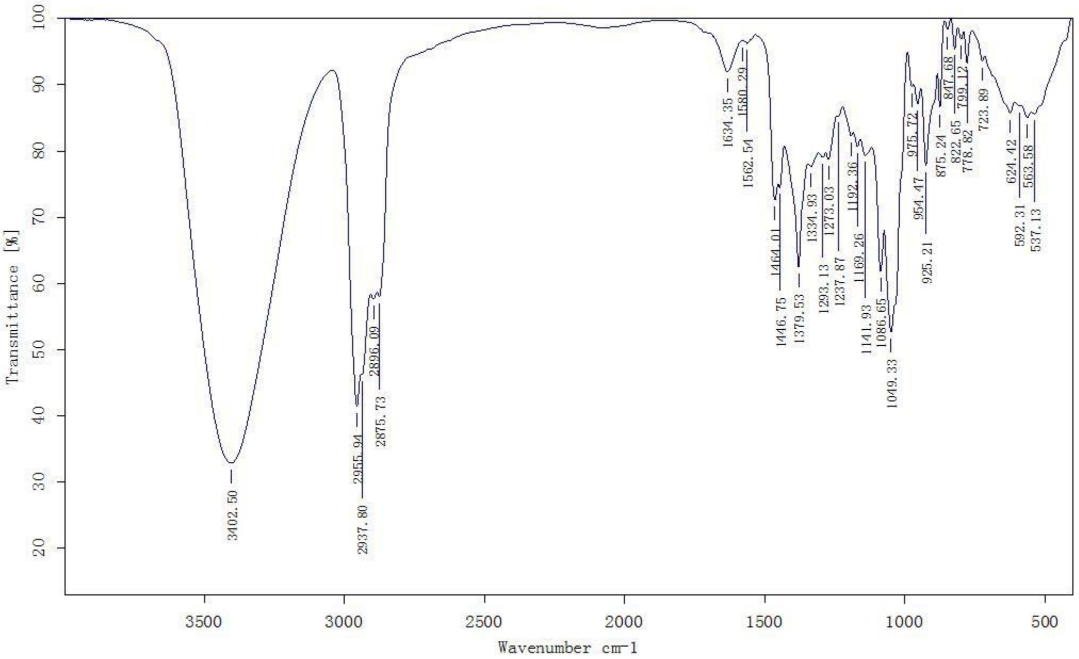

Figure S51. IR spectrum of **5** in methanol.

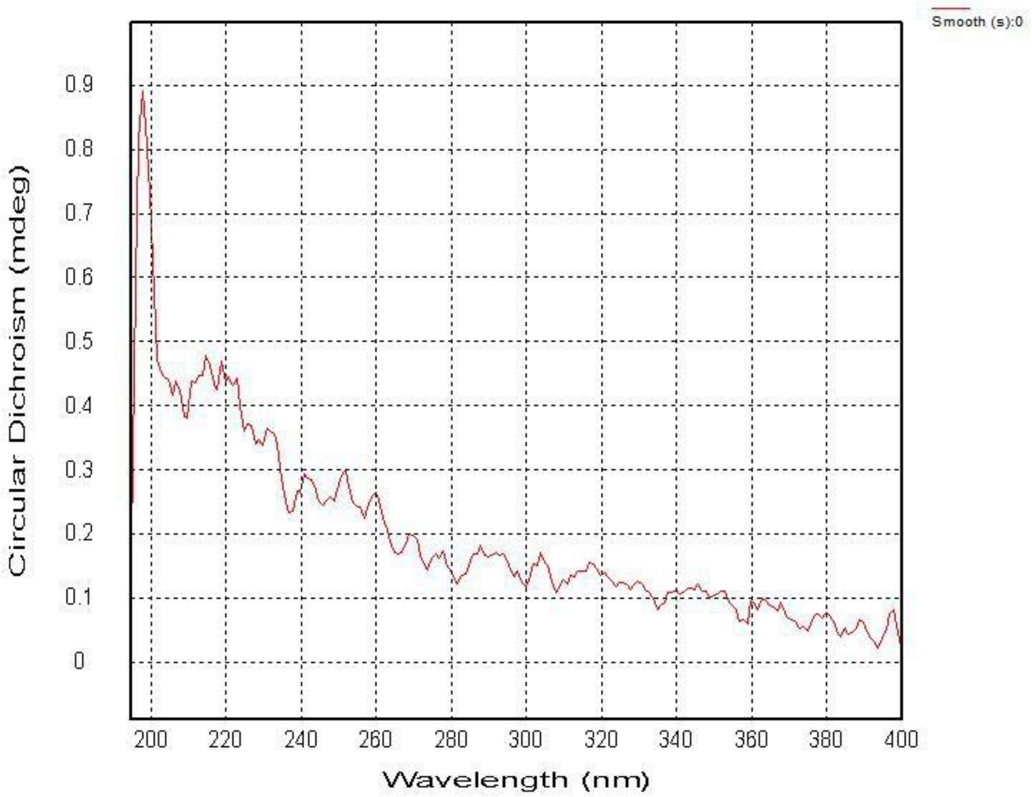

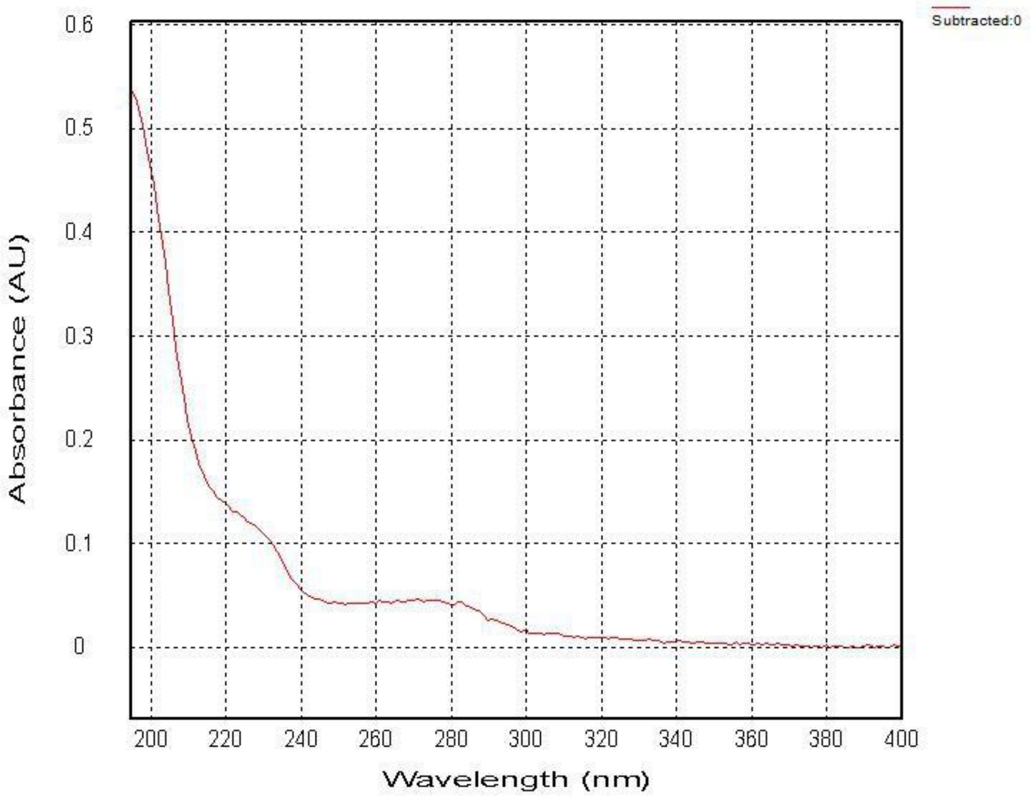

Figure S52. CD spectrum of **5** in methanol.

Rudolph Research Analytical

This sample was measured on an Autopol VI, Serial #91058
Manufactured by Rudolph Research Analytical, Hackettstown, NJ, USA.

Measurement Date : Thursday, 07-APR-2022

Set Temperature : 20.0

Time Delay : Disabled

Delay between Measurement : Disabled

n	Average	Std.Dev.	% RSD	Maximum	Minimum
5	-4.50	0.45	-10.00	-4.17	-5.00

S.No	Sample ID	Time	Result	Scale	OR °Arc	WLG.nm	Lg.mm	Conc.g/100ml	Temp.
1	05R53	11:31:06 AM	-4.17	SR	-0.005	589	100.00	0.120	20.7
2	05R53	11:31:13 AM	-5.00	SR	-0.006	589	100.00	0.120	20.6
3	05R53	11:31:20 AM	-5.00	SR	-0.006	589	100.00	0.120	20.5
4	05R53	11:31:26 AM	-4.17	SR	-0.005	589	100.00	0.120	20.4
5	05R53	11:31:33 AM	-4.17	SR	-0.005	589	100.00	0.120	20.3

Figure S53. Specific Rotation of **5** in methanol.

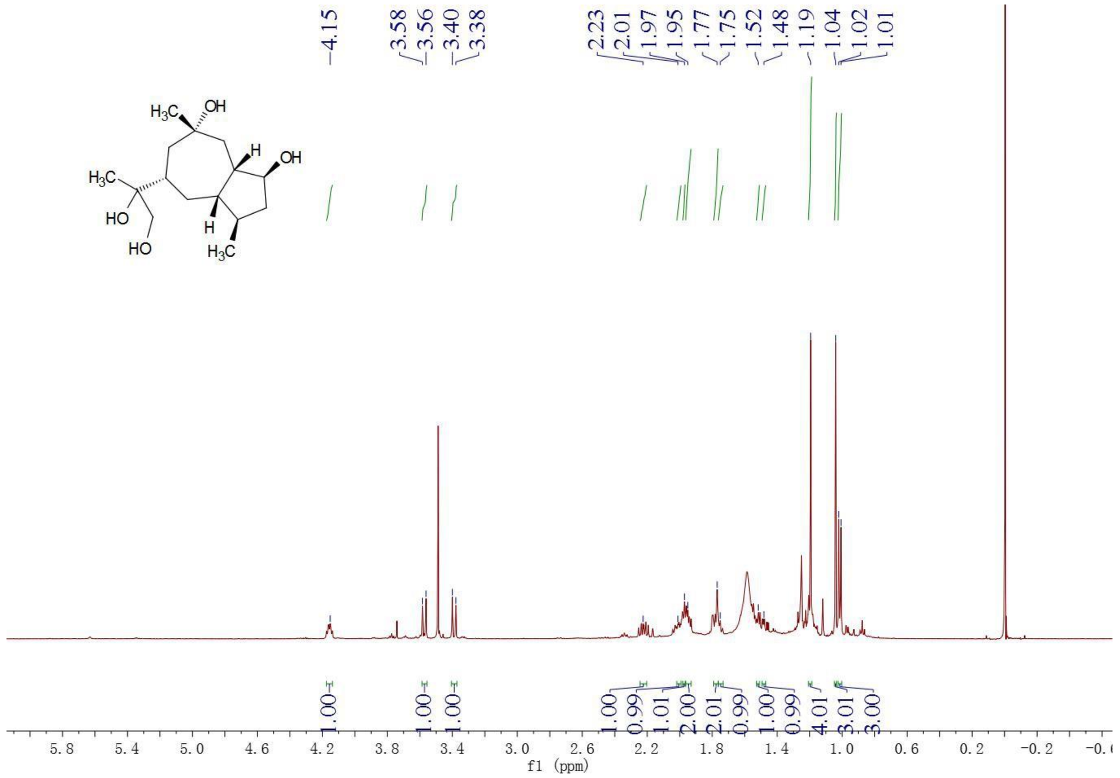

Figure S54. ^{1}H NMR spectrum of compound **6** in CDCl3.

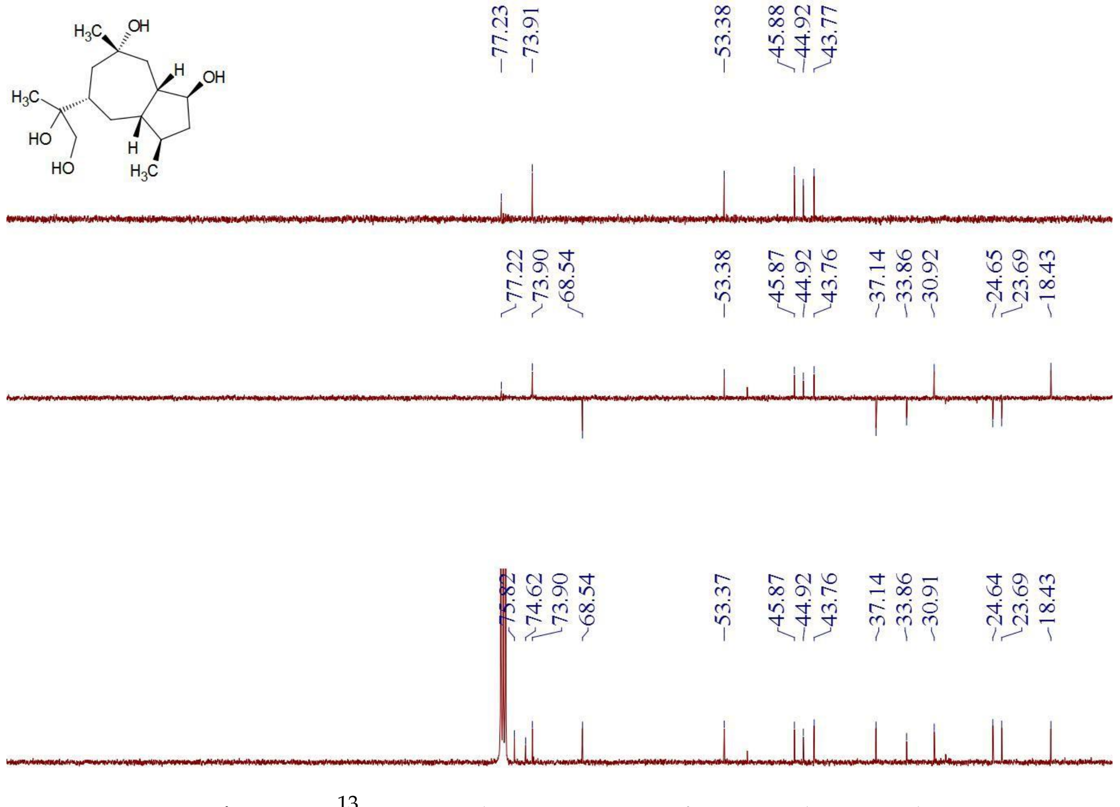

Figure S55. ^{13}C NMR and DEPT spectrum of compound **6** in CDCl3.

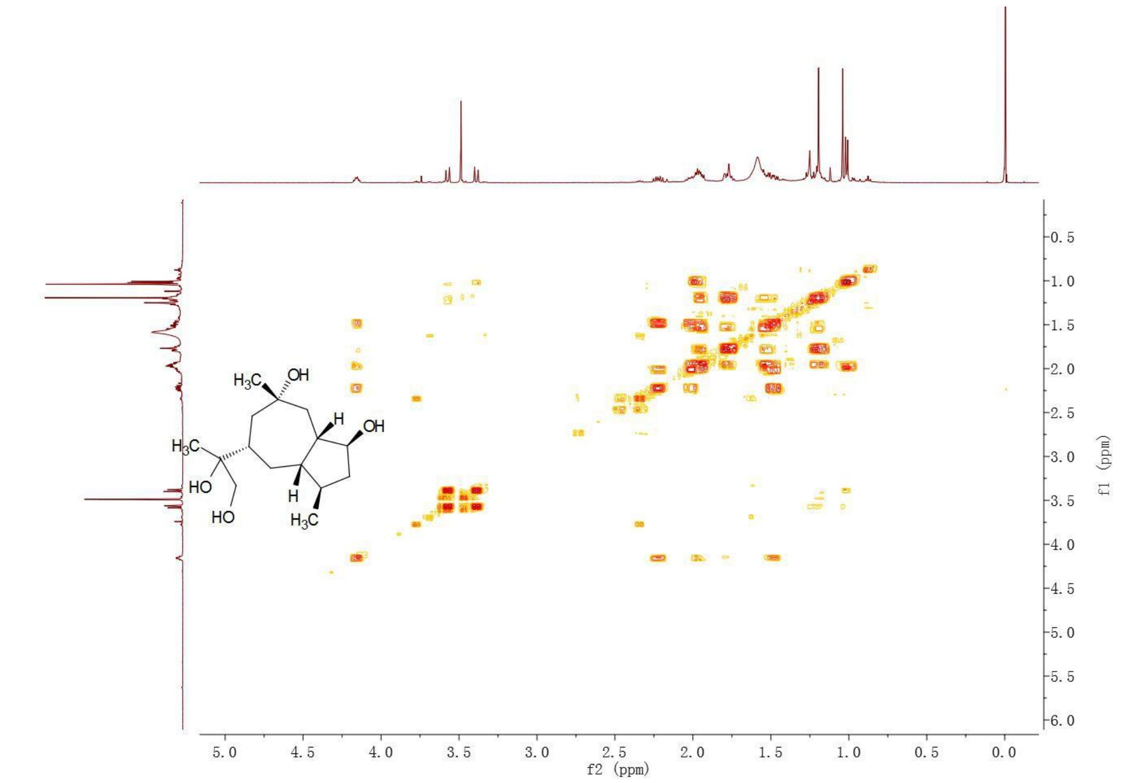

Figure S56. ^{1}H-^{1}H COSY spectrum of compound **6** in CDCl3.

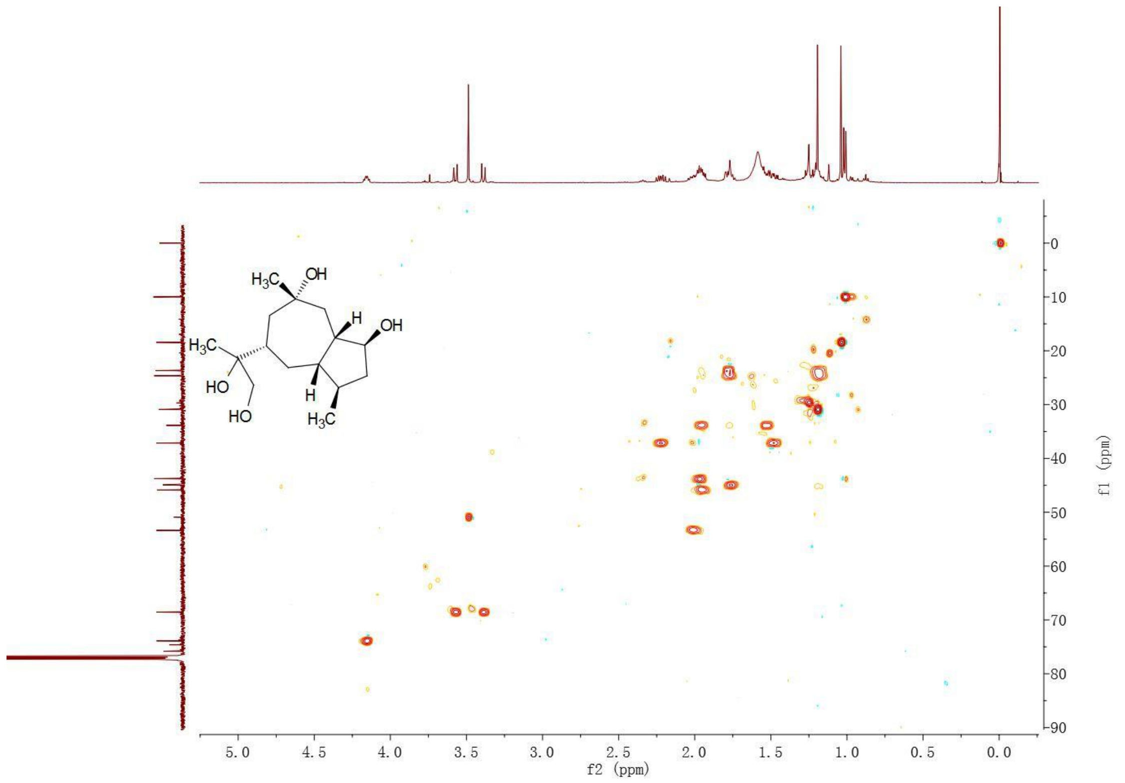

Figure S57. HSQC spectrum of compound **6** in CDCl3.

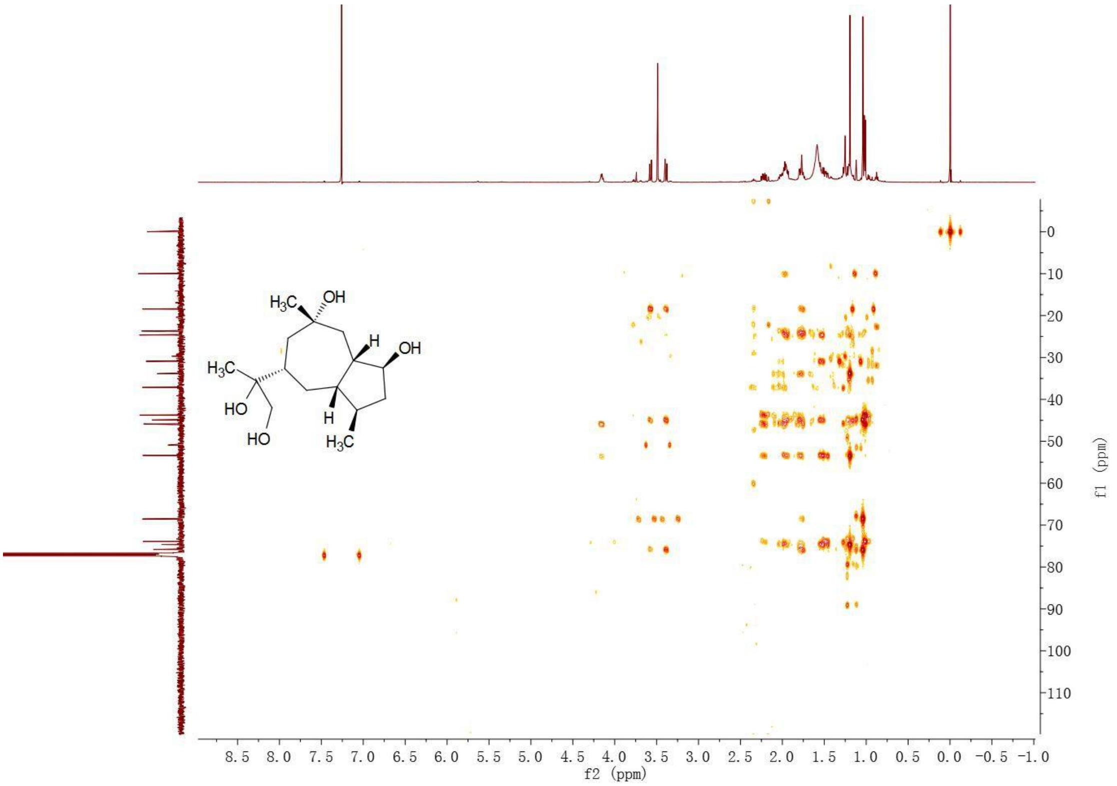

Figure S58. HMBC spectrum of compound **6** in CDCl3.

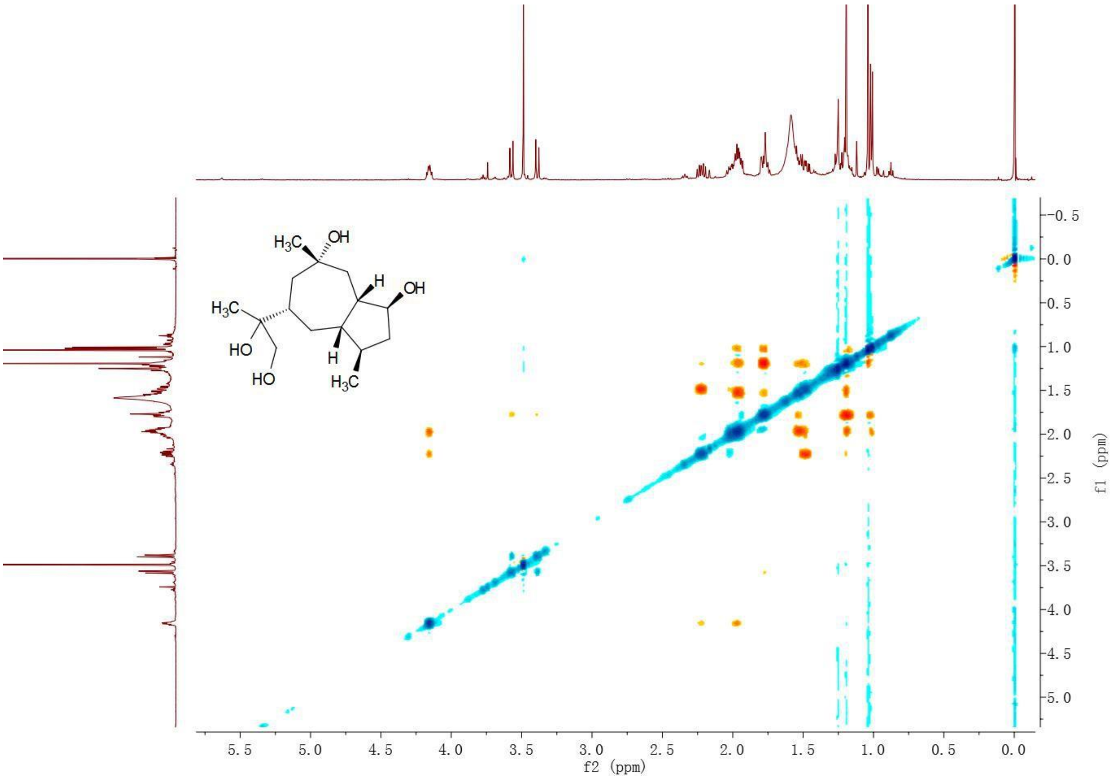

Figure S59. ROESY spectrum of compound **6** in CDCl3.

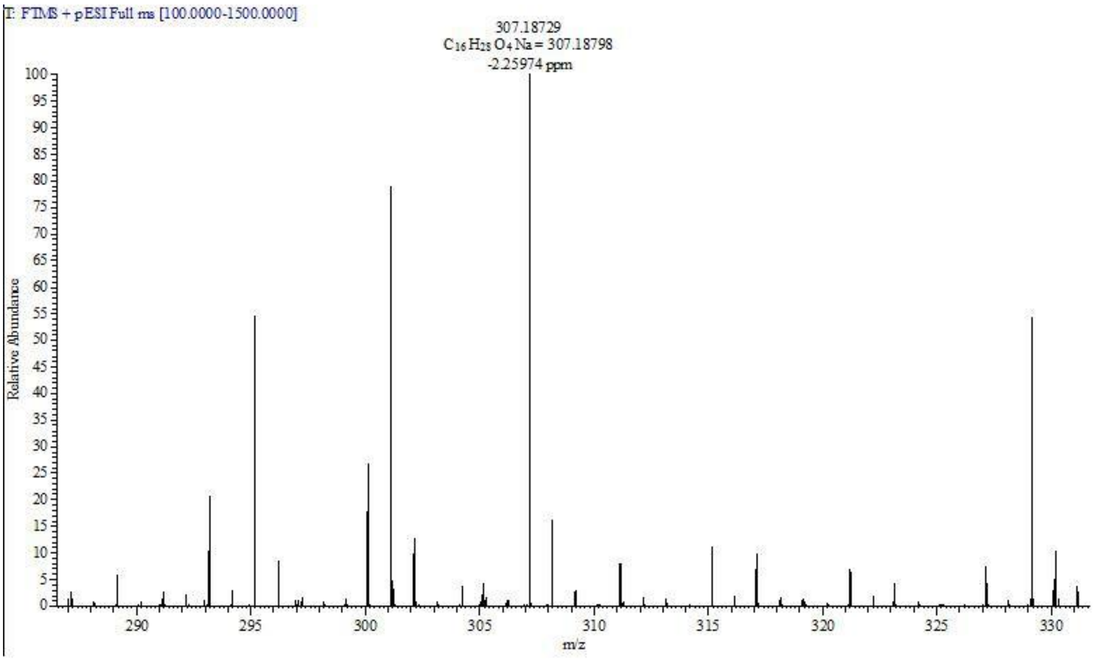

Figure S60. HRESIMS spectrum of compound **6.**

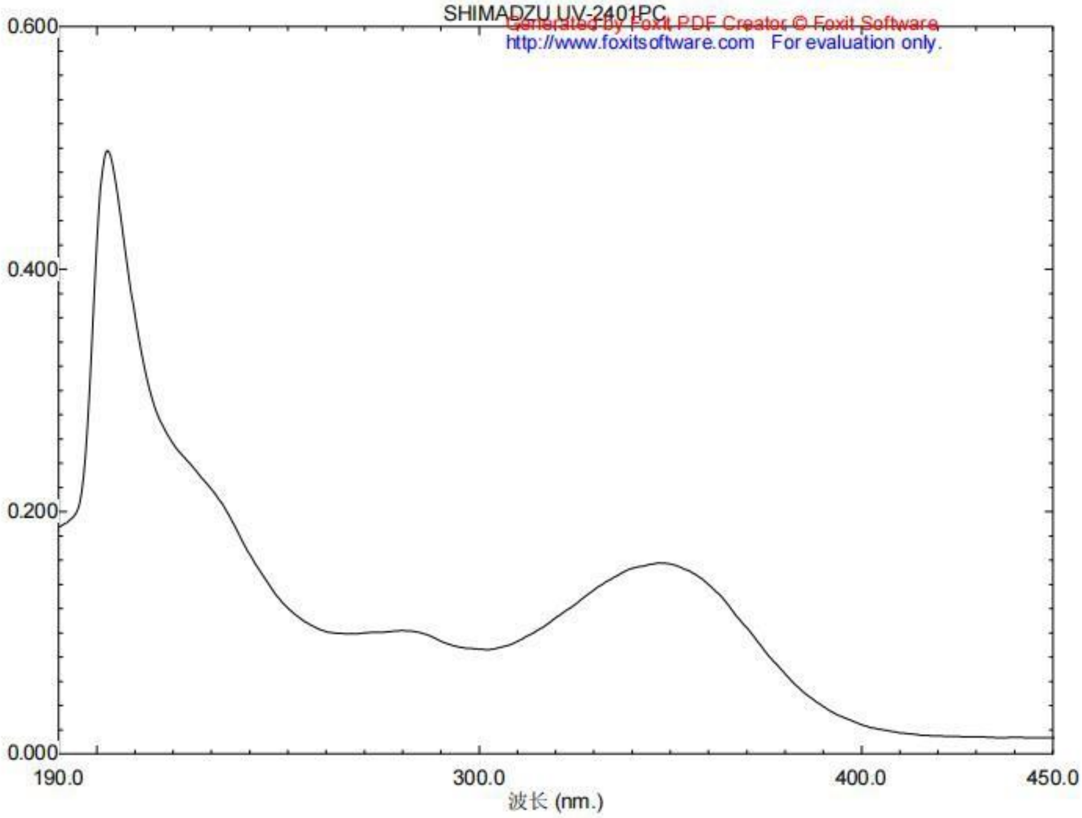

Figure S61. UV spectrum of **6** in methanol.

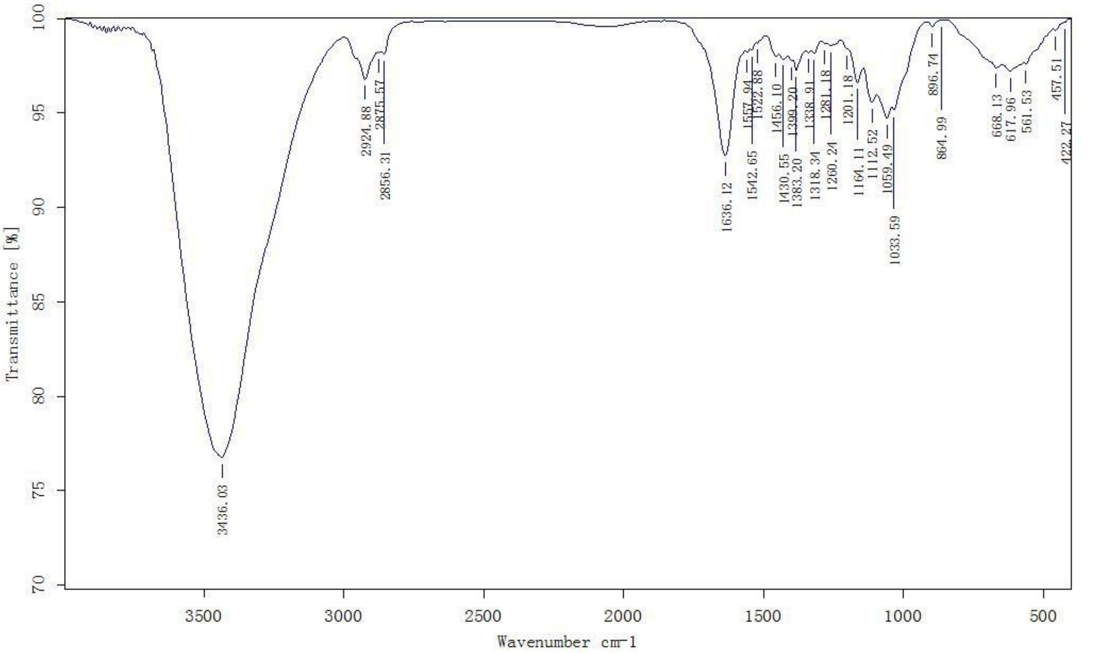

Figure S62. IR spectrum of **6** in methanol.

Rudolph Research Analytical

This sample was measured on an Autopol VI, Serial #91058
Manufactured by Rudolph Research Analytical, Hackettstown, NJ, USA.

Measurement Date : Saturday, 18-JUN-2022

Set Temperature : OFF

Time Delay : Disabled

Delay between Measurement : Disabled

n	Average	Std.Dev.	% RSD	Maximum	Minimum
5	-11.30	0.86	-7.61	-10.50	-12.70

S.No	Sample ID	Time	Result	Scale	OR °Arc	WLG.nm	Lg.mm	Conc.g/100ml	Temp.
1	05R55	04:44:29 PM	-10.80	SR	-0.0108	589	100.00	0.100	23.8
2	05R55	04:44:37 PM	-10.50	SR	-0.0105	589	100.00	0.100	23.8
3	05R55	04:44:45 PM	-12.70	SR	-0.0127	589	100.00	0.100	23.8
4	05R55	04:44:53 PM	-11.00	SR	-0.0110	589	100.00	0.100	23.8
5	05R55	04:45:01 PM	-11.50	SR	-0.0115	589	100.00	0.100	23.8

Figure S63. Specific Rotation of **6** in methanol.

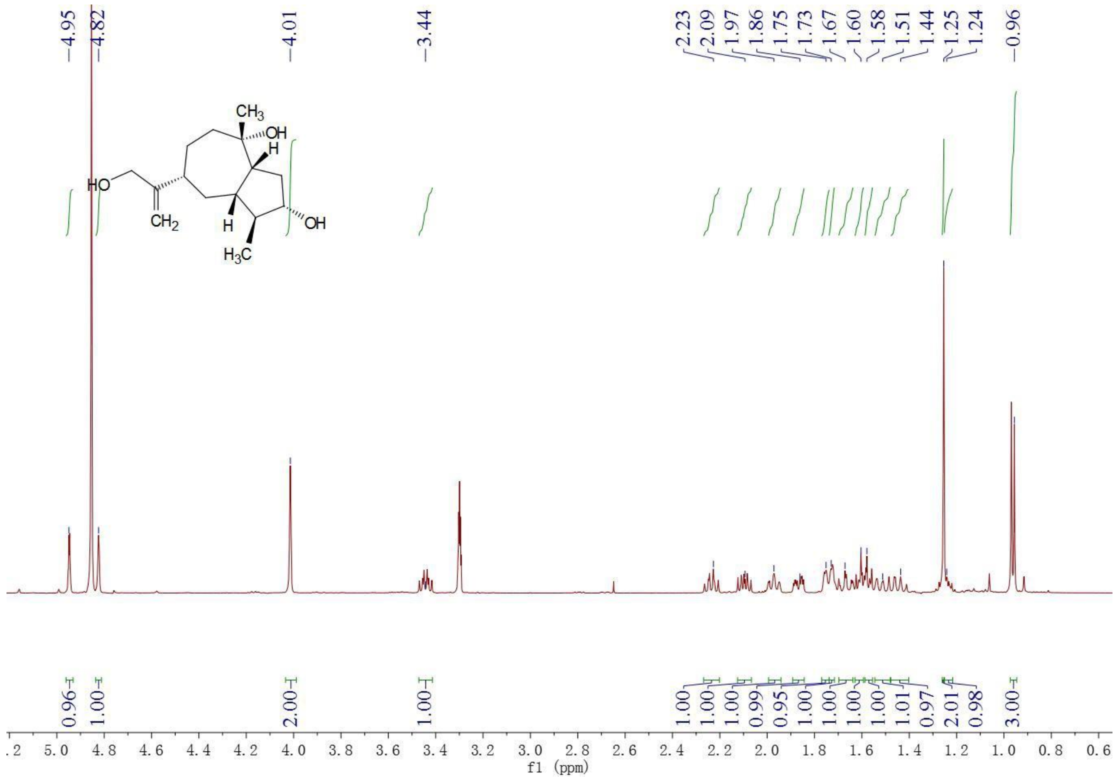

Figure S64. ^{1}H NMR spectrum of compound **7** in CD3OD.

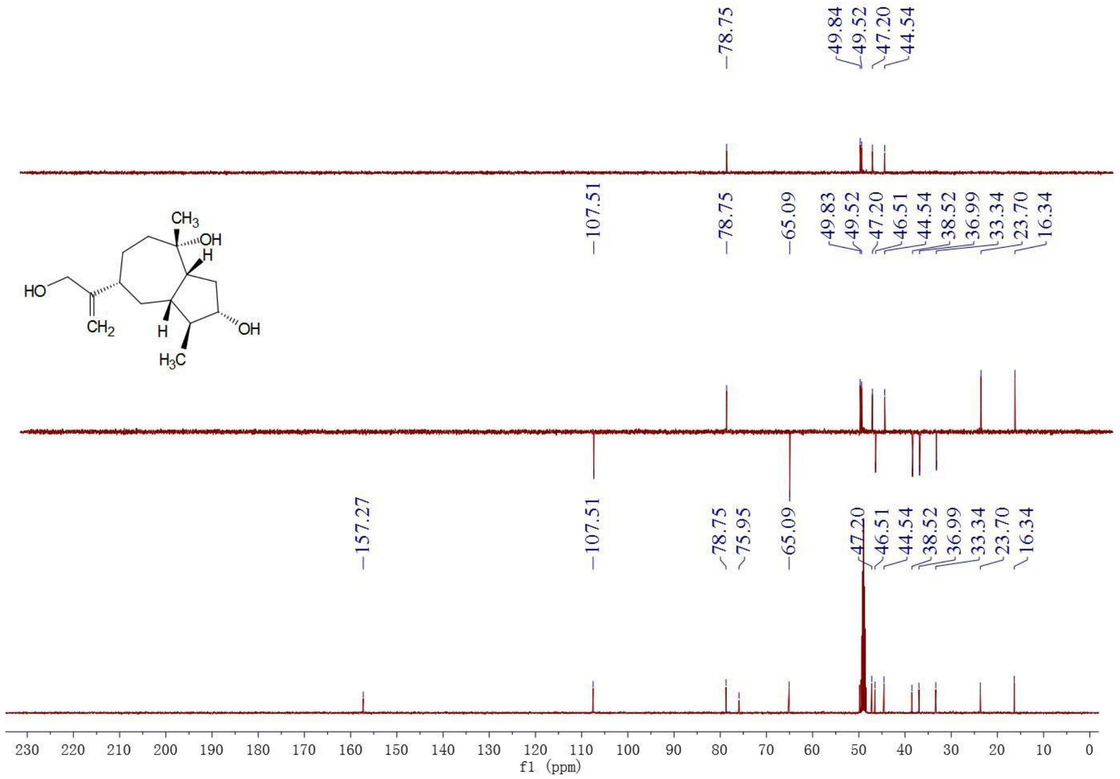

Figure S65. ^{13}C NMR and DEPT spectrum of compound **7** in CD3OD.

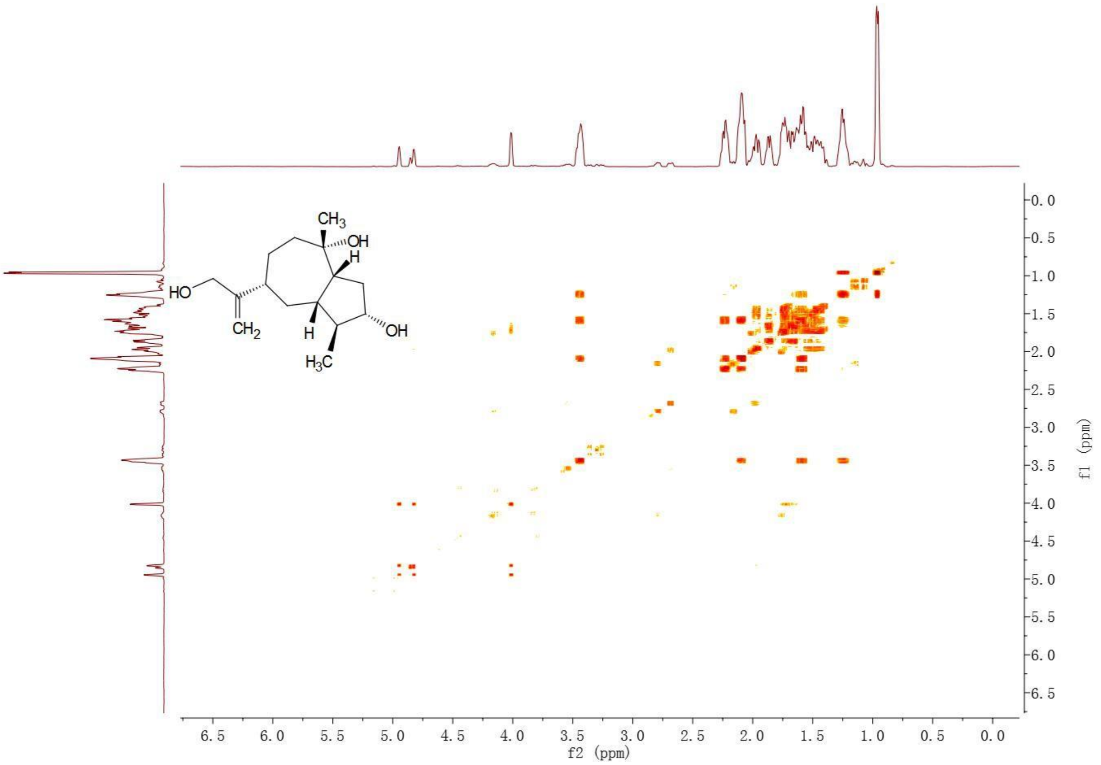

Figure S66. ^{1}H-^{1}H COSY spectrum of compound **7** in CD3OD.

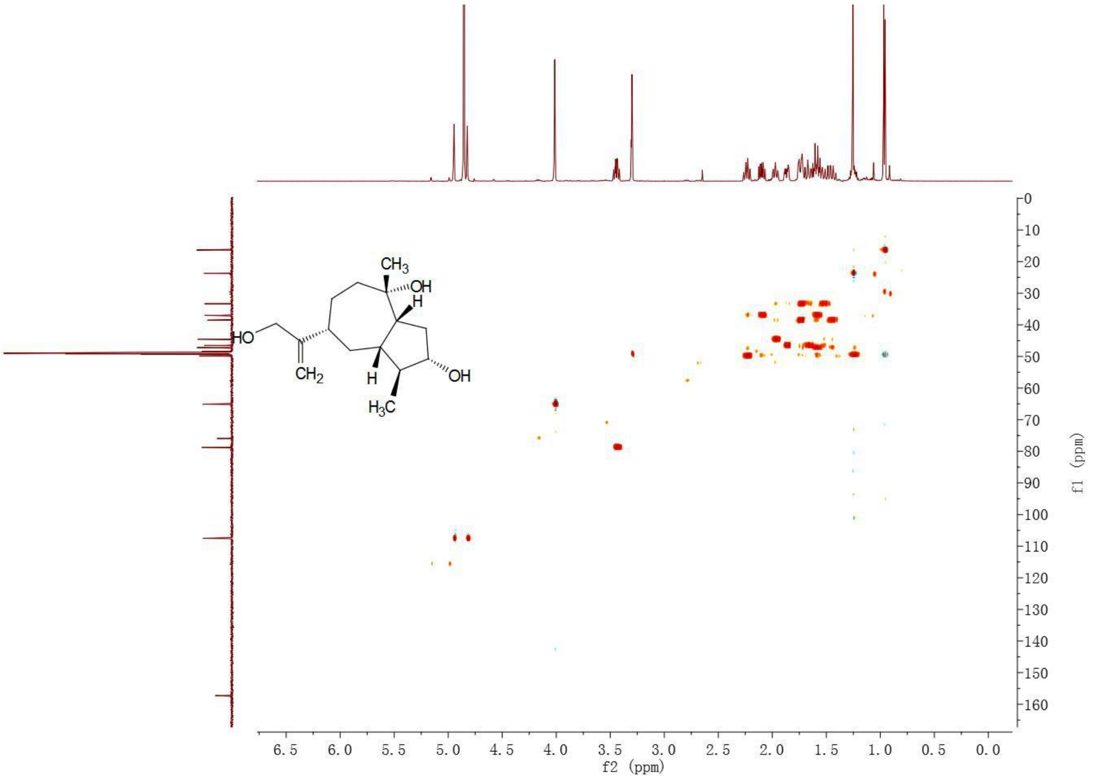

Figure S67. HSQC spectrum of compound **7** in CD3OD.

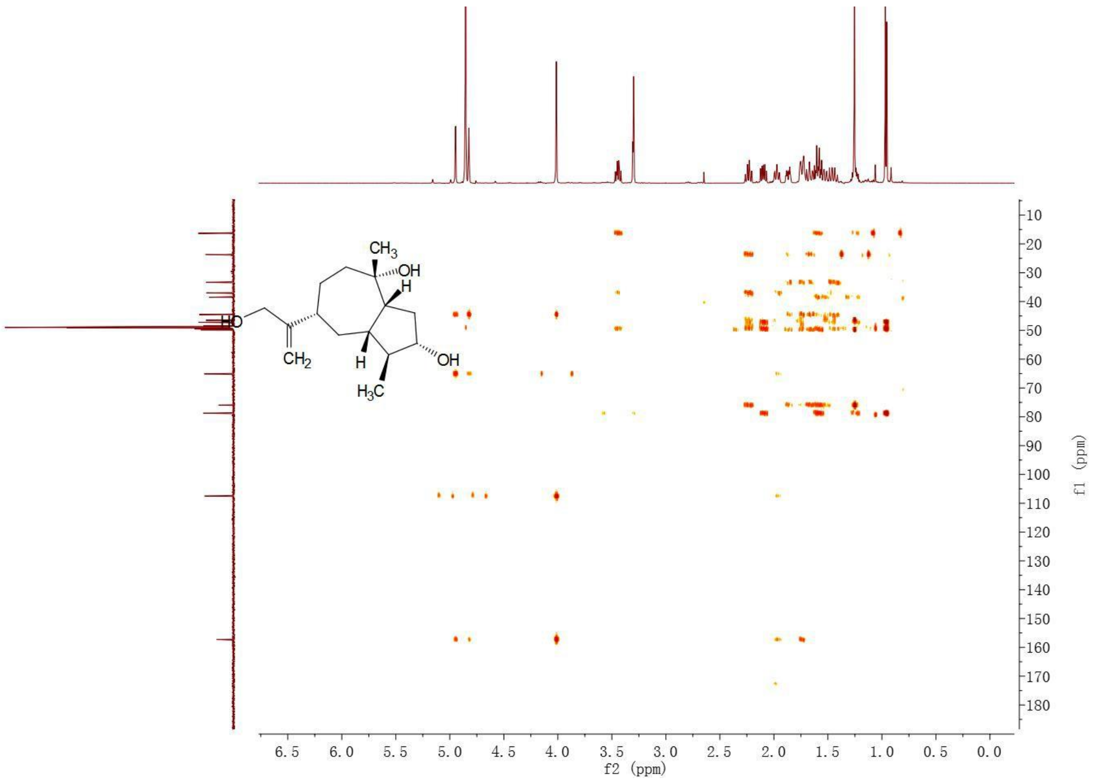

Figure S68. HMBC spectrum of compound **7** in CD3OD.

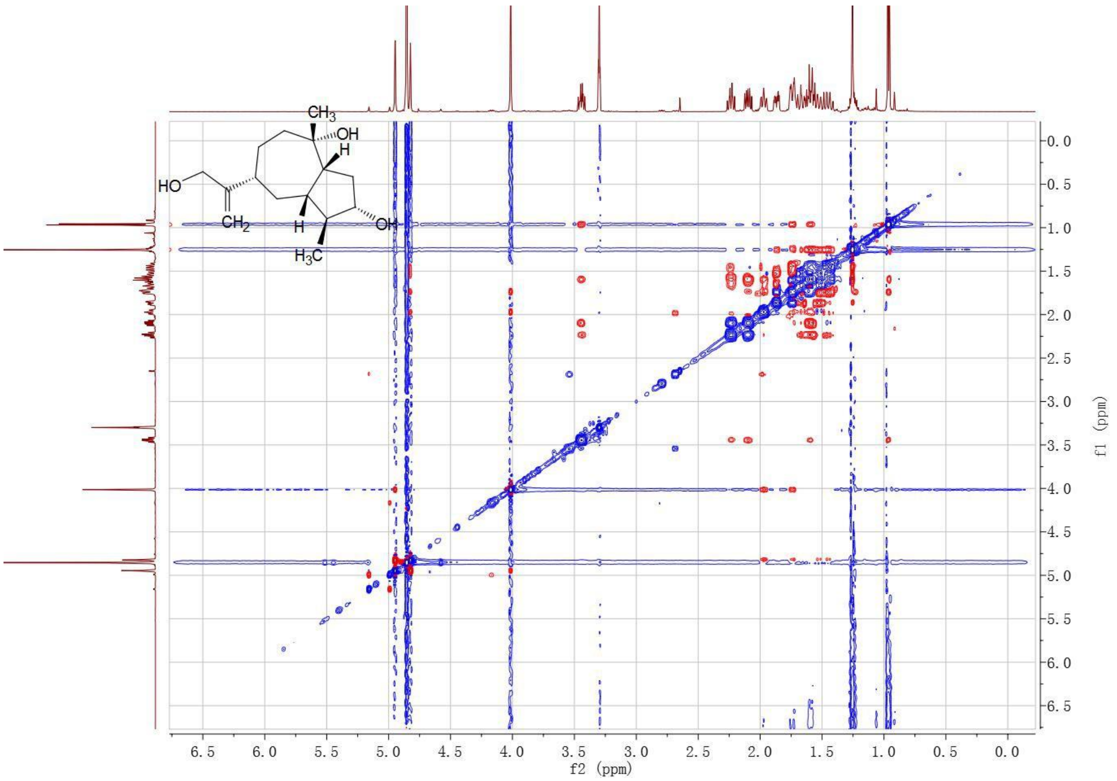

Figure S69. ROESY spectrum of compound **7** in CD3OD.

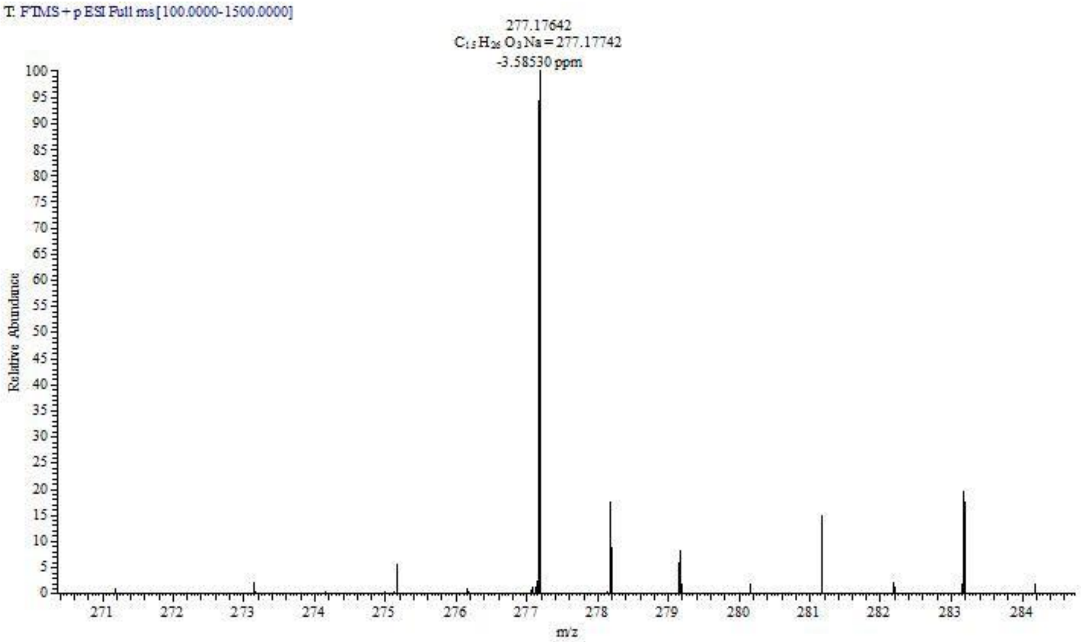

Figure S70. HRESIMS spectrum of compound **7.**

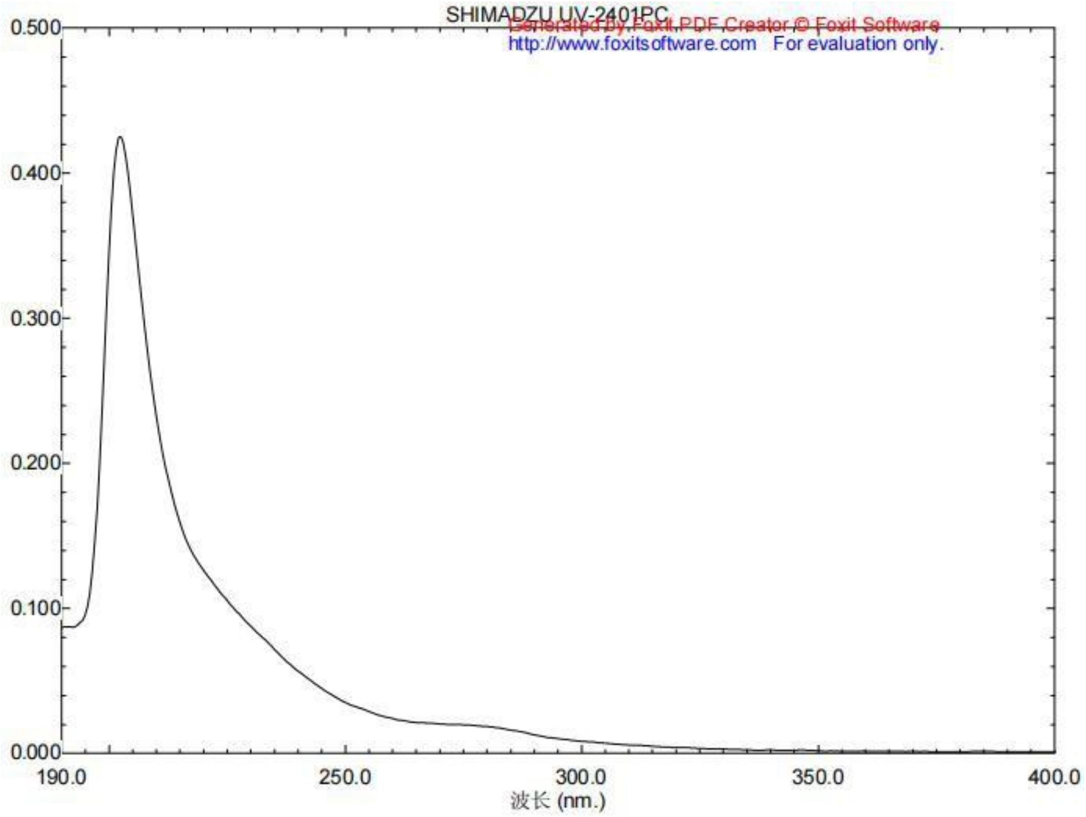

Figure S71. UV spectrum of **7** in methanol.

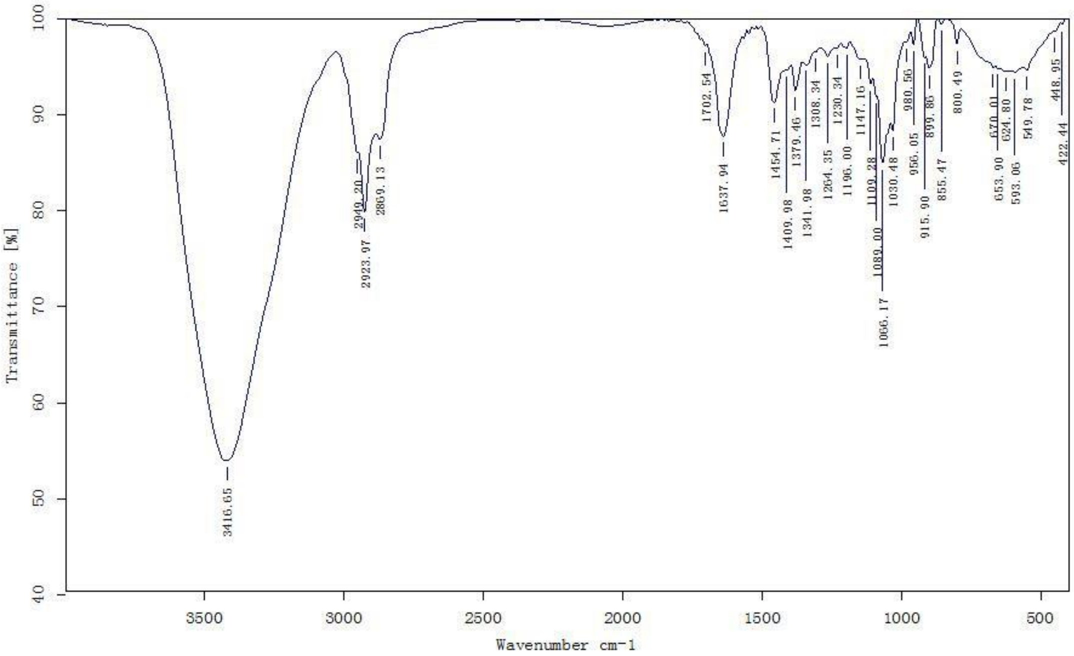

Figure S72. IR spectrum of **7** in methanol.

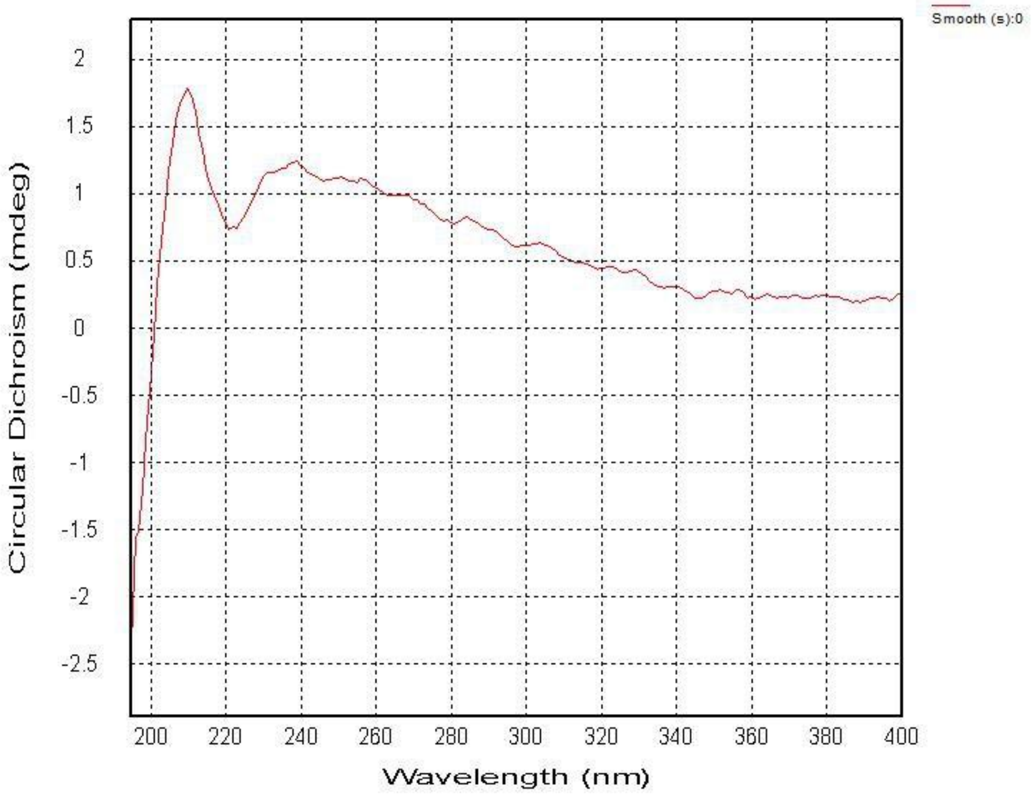

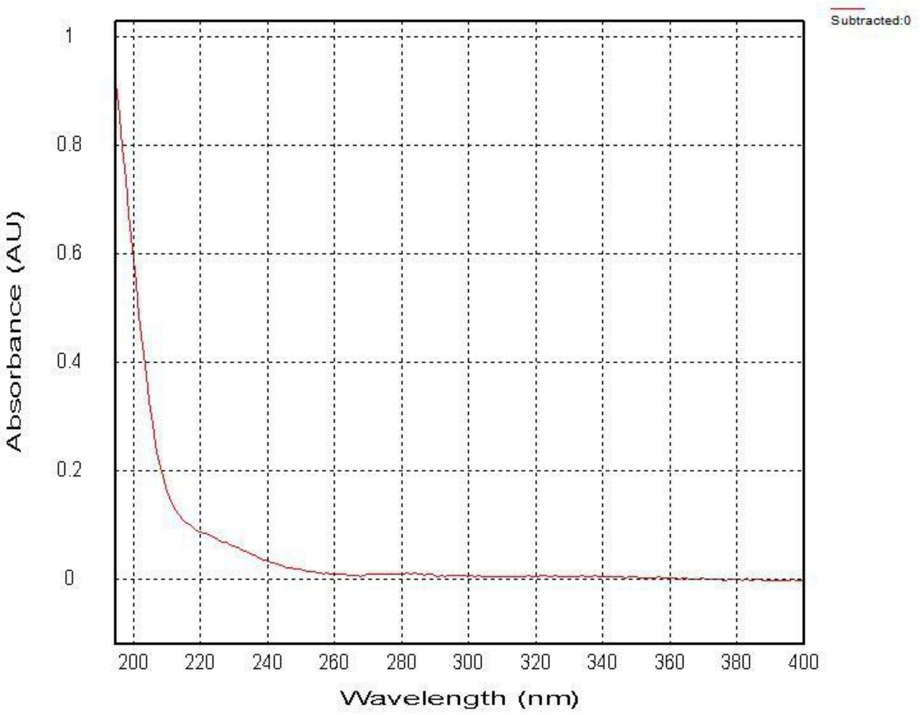

Figure S73. a CD spectrum of **7** in methanol.

Rudolph Research Analytical

This sample was measured on an Autopol VI, Serial #91058
Manufactured by Rudolph Research Analytical, Hackettstown, NJ, USA.

Measurement Date : Saturday, 28-MAY-2022

Set Temperature : OFF

Time Delay : Disabled

Delay between Measurement : Disabled

n	Average	Std.Dev.	% RSD	Maximum	Minimum
5	-15.25	0.21	-1.37	-15.05	-15.50

S.No	Sample ID	Time	Result	Scale	OR °Arc	WLG.nm	Lg.mm	Conc.g/100ml	Temp.
1	05R58	11:17:06 AM	-15.45	SR	-0.0309	589	100.00	0.200	24.6
2	05R58	11:17:14 AM	-15.05	SR	-0.0301	589	100.00	0.200	24.6
3	05R58	11:17:23 AM	-15.50	SR	-0.0310	589	100.00	0.200	24.6
4	05R58	11:17:30 AM	-15.10	SR	-0.0302	589	100.00	0.200	24.6
5	05R58	11:17:39 AM	-15.15	SR	-0.0303	589	100.00	0.200	24.6

Figure S73. b Specific Rotation of **7** in methanol.

Three conformers for **1a**

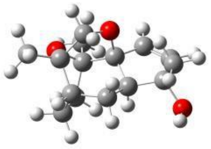

1a_1

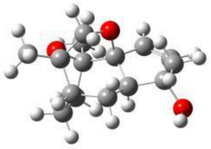

1a_2

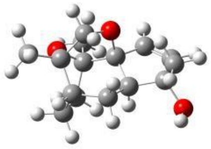

1a_3

Figure S74. Optimized geometries of predominant conformers for compound 1a at the B3LYP/6-31G(d) level.

Three conformers for **1b**

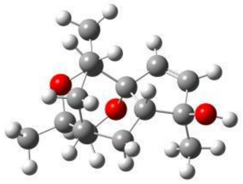

1b_1

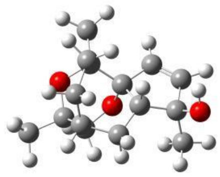

1b_2

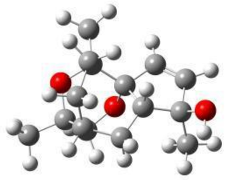

1b_3

Figure S75. Optimized geometries of predominant conformers for compound **1b** at the B3LYP/6-31G(d) level.

Table S1. Boltzmann distribution of conformers **1a_1–1a_3.**

	Conformer	E(Hartree)	ΔG(kcal/mol)	Proportion(%)
	**1a_1**	-810.157367	0.336345038	28.52
	**1a_2**	-810.157903	0	50.34
**1a**	**1a_3**	-810.157084	0.513930198	21.13

Table S2. Cartesian coordinates of conformers **1a_1–1a_2.**

Atomic Type	1a_1			1a_2		
	X	Y	Z	X	Y	Z
C	-0.329781	0.702491	-0.39409	-0.332725	0.693737	-0.40107
C	-1.056445	-0.279275	0.568202	-1.056214	-0.280924	0.571146
C	1.022015	1.310801	0.189614	1.016941	1.315123	0.173949
C	-0.305577	-1.594106	0.771802	-0.299376	-1.589374	0.791231
H	-1.108574	0.225346	1.536037	-1.097055	0.236327	1.534684
O	-0.041135	0.081734	-1.674432	-0.042716	0.059556	-1.674044
C	1.175389	1.12046	1.710982	1.172872	1.143992	1.697354
O	2.143794	0.59761	-0.374959	2.140788	0.599051	-0.38241
C	1.215054	-1.338618	0.877691	1.220168	-1.325839	0.894306
H	-0.509422	-2.304978	-0.035015	-0.500644	-2.309895	-0.007497
H	-0.64901	-2.07635	1.695237	-0.641863	-2.062495	1.719776
C	0.978216	-0.927217	-1.690121	0.980494	-0.94594	-1.678294
C	1.556932	-0.355673	2.024533	1.55802	-0.327431	2.029085
H	1.984909	1.779158	2.0455	1.981555	1.808506	2.022094
H	0.278422	1.442418	2.249658	0.276393	1.471493	2.233786
C	1.876343	-0.816136	-0.456807	1.87871	-0.816706	-0.446844
H	1.685183	-2.301077	1.113138	1.694761	-2.283164	1.141417
H	1.584808	-0.73852	-2.583619	1.585698	-0.765108	-2.574252
H	0.525547	-1.922193	-1.794391	0.531294	-1.943564	-1.770787
H	2.635367	-0.407413	2.208993	2.636386	-0.373801	2.215112
H	1.071053	-0.694012	2.94708	1.072482	-0.655825	2.955414
C	3.221744	-1.496362	-0.694793	3.226597	-1.494508	-0.677397
H	3.850906	-1.427951	0.199076	3.85586	-1.412994	0.215294
H	3.750307	-1.018555	-1.526107	3.752886	-1.024599	-1.51461
H	3.080157	-2.556168	-0.933891	3.088949	-2.557612	-0.903752
C	-1.453912	1.676557	-0.709045	-1.462009	1.658625	-0.729433
H	-1.286691	2.644419	-1.16857	-1.298165	2.620697	-1.202009
C	-2.646579	1.132318	-0.459861	-2.653834	1.111308	-0.480048
H	-3.610125	1.594557	-0.662589	-3.619942	1.562158	-0.6938
C	-2.538499	-0.271664	0.114074	-2.544908	-0.283116	0.112605
C	-2.923418	-1.335825	-0.920229	-2.916349	-1.361798	-0.907215
H	-2.852638	-2.337598	-0.482556	-2.861964	-2.354623	-0.447135
H	-3.95724	-1.184463	-1.256638	-3.941702	-1.207281	-1.260214
H	-2.269382	-1.280183	-1.79447	-2.237652	-1.325697	-1.762286
O	-3.338093	-0.436342	1.301467	-3.446849	-0.485048	1.214329
H	-4.264487	-0.515869	1.019928	-3.30467	0.242225	1.842776
C	1.223642	2.764167	-0.230622	1.21215	2.763946	-0.264454
H	0.509887	3.425525	0.270895	0.495662	3.42853	0.228913
H	2.235394	3.081613	0.041375	2.222522	3.089189	0.003327
H	1.10836	2.876772	-1.313663	1.096207	2.862491	-1.348784

Table S3. Cartesian coordinates of conformers **1a_3.**

Atomic Type	(+)-1c		
	X	Y	Z
C	-0.329135	0.702433	-0.389408
C	-1.05673	-0.283601	0.568713
C	1.022177	1.31006	0.196861
C	-0.303644	-1.599116	0.76435
H	-1.108923	0.216746	1.538429
O	-0.039285	0.090623	-1.673525
C	1.175569	1.112619	1.717228
O	2.1444	0.600355	-0.371032
C	1.217017	-1.342594	0.871932
H	-0.506475	-2.305874	-0.04653
H	-0.642819	-2.089527	1.685939
C	0.979559	-0.918585	-1.693959
C	1.557464	-0.364949	2.023698
H	1.984942	1.769908	2.054907
H	0.278275	1.431447	2.257145
C	1.877846	-0.812831	-0.460087
H	1.688174	-2.305706	1.102663
H	1.585868	-0.726031	-2.586825
H	0.526972	-1.913169	-1.80248
H	2.635816	-0.417322	2.208525
H	1.071221	-0.708038	2.944345
C	3.223444	-1.491454	-0.701509
H	3.852697	-1.427126	0.192626
H	3.751702	-1.009372	-1.53056
H	3.082174	-2.550136	-0.945762
C	-1.454276	1.676791	-0.699234
H	-1.288814	2.64229	-1.164013
C	-2.647856	1.138482	-0.442463
H	-3.611708	1.601684	-0.637071
C	-2.546155	-0.267588	0.113649
C	-2.922831	-1.315662	-0.942344
H	-2.861958	-2.328846	-0.524106
H	-3.950972	-1.152068	-1.283002
H	-2.250197	-1.257373	-1.80202
O	-3.444761	-0.368686	1.233453
H	-3.448652	-1.297572	1.517243
C	1.221589	2.765623	-0.216739
H	0.506014	3.423333	0.287053
H	2.232469	3.083922	0.057613
H	1.107138	2.882726	-1.299415

Table S4. Boltzmann distribution of conformers **1b_1–1b_3.**

	Conformer	E(Hartree)	ΔG(kcal/mol)	Proportion(%)
1b	**1b_1**	-810.157367	0.336345038	28.52
	**1b_2**	-810.157903	0	50.34
	**1b_3**	-810.157084	0.513930198	21.13

Table S5. Cartesian coordinates of conformers **1b_1–1b_2.**

Atomic Type	1b_1			1b_2		
	X	Y	Z	X	Y	Z
C	0.329781	0.702491	-0.39409	0.332725	0.693737	-0.40107
C	1.056445	-0.279275	0.568202	1.056214	-0.280924	0.571146
C	-1.022015	1.310801	0.189614	-1.016941	1.315123	0.173949
C	0.305577	-1.594106	0.771802	0.299376	-1.589374	0.791231
H	1.108574	0.225346	1.536037	1.097055	0.236327	1.534684
O	0.041135	0.081734	-1.674432	0.042716	0.059556	-1.674044
C	-1.175389	1.12046	1.710982	-1.172872	1.143992	1.697354
O	-2.143794	0.59761	-0.374959	-2.140788	0.599051	-0.38241
C	-1.215054	-1.338618	0.877691	-1.220168	-1.325839	0.894306
H	0.509422	-2.304978	-0.035015	0.500644	-2.309895	-0.007497
H	0.64901	-2.07635	1.695237	0.641863	-2.062495	1.719776
C	-0.978216	-0.927217	-1.690121	-0.980494	-0.94594	-1.678294
C	-1.556932	-0.355673	2.024533	-1.55802	-0.327431	2.029085

H	-1.984909	1.779158	2.0455	-1.981555	1.808506	2.022094
H	-0.278422	1.442418	2.249658	-0.276393	1.471493	2.233786
C	-1.876343	-0.816136	-0.456807	-1.87871	-0.816706	-0.446844
H	-1.685183	-2.301077	1.113138	-1.694761	-2.283164	1.141417
H	-1.584808	-0.73852	-2.583619	-1.585698	-0.765108	-2.574252
H	-0.525547	-1.922193	-1.79439	-0.531294	-1.943564	-1.770787
H	-2.635367	-0.407413	2.208993	-2.636386	-0.373801	2.215112
H	-1.071053	-0.694012	2.94708	-1.072482	-0.655825	2.955414
C	-3.221744	-1.496362	-0.694793	-3.226597	-1.494508	-0.677397
H	-3.850906	-1.427951	0.199076	-3.85586	-1.412994	0.215294
H	-3.750307	-1.018555	-1.526107	-3.752886	-1.024599	-1.51461
H	-3.080157	-2.556168	-0.933891	-3.088949	-2.557612	-0.903752
C	1.453912	1.676557	-0.709045	1.462009	1.658625	-0.729433
H	1.286691	2.644419	-1.16857	1.298165	2.620697	-1.202009
C	2.646579	1.132318	-0.459861	2.653834	1.111308	-0.480048
H	3.610125	1.594557	-0.662589	3.619942	1.562158	-0.6938
C	2.538499	-0.271664	0.114074	2.544908	-0.283116	0.112605
C	2.923418	-1.335825	-0.920229	2.916349	-1.361798	-0.907215
H	2.852638	-2.337598	-0.482556	2.861964	-2.354623	-0.447135
H	3.95724	-1.184463	-1.256638	3.941702	-1.207281	-1.260214
H	2.269382	-1.280183	-1.79447	2.237652	-1.325697	-1.762286
O	3.338093	-0.436342	1.301467	3.446849	-0.485048	1.214329
H	4.264487	-0.515869	1.019928	3.30467	0.242225	1.842776
C	-1.223642	2.764167	-0.230622	-1.21215	2.763946	-0.264454
H	-0.509887	3.425525	0.270895	-0.495662	3.428529	0.228913
H	-2.235394	3.081613	0.041375	-2.222522	3.089189	0.003327
H	-1.10836	2.876772	-1.313663	-1.096207	2.862491	-1.348784

Table S6. Cartesian coordinates of conformers **1b_3.**

**Atomic Type**	**1b_3**		
	**X**	**Y**	**Z**
C	0.329135	0.702433	-0.38941
C	1.05673	-0.2836	0.568713
C	-1.02218	1.31006	0.196861
C	0.303644	-1.59912	0.76435
H	1.108923	0.216746	1.538429
O	0.039285	0.090623	-1.67353
C	-1.17557	1.112619	1.717228
O	-2.1444	0.600355	-0.37103
C	-1.21702	-1.34259	0.871932
H	0.506475	-2.30587	-0.04653
H	0.642819	-2.08953	1.685939
C	-0.97956	-0.91859	-1.69396
C	-1.55746	-0.36495	2.023698
H	-1.98494	1.769908	2.054907
H	-0.27828	1.431447	2.257145
C	-1.87785	-0.81283	-0.46009
H	-1.68817	-2.30571	1.102663
H	-1.58587	-0.72603	-2.58683
H	-0.52697	-1.91317	-1.80248
H	-2.63582	-0.41732	2.208525
H	-1.07122	-0.70804	2.944345
C	-3.22344	-1.49145	-0.70151
H	-3.8527	-1.42713	0.192626
H	-3.7517	-1.00937	-1.53056
H	-3.08217	-2.55014	-0.94576
C	1.454275	1.676791	-0.69923
H	1.288814	2.64229	-1.16401
C	2.647856	1.138482	-0.44246
H	3.611708	1.601684	-0.63707
C	2.546155	-0.26759	0.113649
C	2.922831	-1.31566	-0.94234
H	2.861958	-2.32885	-0.52411
H	3.950972	-1.15207	-1.283
H	2.250197	-1.25737	-1.80202
O	3.444761	-0.36869	1.233453
H	3.448652	-1.29757	1.517243
C	-1.22159	2.765623	-0.21674
H	-0.50601	3.423333	0.287053
H	-2.23247	3.083922	0.057613

H	-1.10714	2.882726	-1.29942

Table S7. Gibbs free energies[a] and equilibrium populations[b] of low-energy conformers of compound **2**.

Conformers	ΔG(a.u.)	P(%)/100	G(a.u.)
**2_a**	0.0058	0.12	-813.72172
**2_b**	0.0	55.84	-813.727517
**2_c**	0.01318	0.0	-813.714337
**2_d**	0.0014	12.71	-813.726119
**2-e**	0.00714	0.03	-813.720374
**2_f**	0.0037	1.1	-813.723813
**2_g**	0.00058	30.2	-813.726936

[a]wB97M-V/def2-TZVP, in a.u.m[b]From ΔG values at 298.15K.

Table S8. Cartesian coordinates for the low-energy reoptimized random reseach conformers of compound **2** at B3LYP-D3(BJ)/6-31G* level of theory in methanol.

**2_a**			**Standard Orientation (A.U.)**		
**Center number**	**Atomic number**	**Atomic Type**	**X**	**Y**	**Z**
0	6	0	0.783819	-0.605193	3.423168
1	6	0	-0.877688	-2.145008	1.598982
2	6	0	-1.715782	-0.478772	-0.630255
3	6	0	0.274598	0.163238	-2.642882
4	6	0	2.866612	1.249449	-1.818345
5	6	0	4.594293	-0.134303	0.096514
6	6	0	3.622086	-0.478661	2.850835
7	6	0	-0.651123	2.345135	-4.379528
8	6	0	-0.002107	4.732711	-2.832737
9	6	0	2.472811	4.119149	-1.405103
10	8	0	6.916375	1.268129	0.198901
11	6	0	5.355782	-2.70157	-1.012161
12	8	0	2.37878	4.694623	1.227486
13	6	0	-3.38489	2.236634	-5.296052
14	6	0	-3.167898	-3.3918	2.954516
15	6	0	-4.755459	-1.590374	4.584725
16	8	0	-5.754795	0.525914	3.286709
17	1	0	4.001666	1.178141	-3.567079
18	1	0	0.238348	-3.739629	0.835322
19	1	0	0.605445	-1.558235	-3.776992
20	6	0	-2.352007	-5.67545	4.546806
21	1	0	-4.434697	-4.121634	1.449225
22	1	0	0.030383	1.333145	3.501663
23	1	0	0.615736	-1.368878	5.358717
24	1	0	-3.275948	-1.446935	-1.632296
25	1	0	-2.543864	1.255841	0.164396
26	1	0	4.387057	1.098291	3.973175
27	1	0	4.546104	-2.199594	3.592946
28	1	0	0.580969	2.331515	-6.069204
29	1	0	0.145626	6.435773	-4.02871
30	1	0	-1.499321	5.082697	-1.42059
31	1	0	4.08501	5.152567	-2.243022
32	1	0	6.523088	2.846106	1.05767
33	1	0	3.738574	-4.011252	-1.094683
34	1	0	6.126626	-2.469842	-2.936685
35	1	0	6.83905	-3.551731	0.180812
36	1	0	2.001495	6.480996	1.421624
37	1	0	-3.774232	0.451724	-6.309571
38	1	0	-4.739286	2.385987	-3.716097
39	1	0	-3.783488	3.810033	-6.611066
40	1	0	-3.597463	-0.801852	6.127308
41	1	0	-6.294995	-2.696508	5.49072
42	1	0	-6.652676	-0.077558	1.802927
43	1	0	-3.994586	-6.613071	5.432775
44	1	0	-1.368029	-7.089827	3.369533
45	1	0	-1.048356	-5.11572	6.079714
**2_b**			Standard Orientation (A.U.)		
Center number	Atomic number	Atomic Type	X	Y	Z

0	6	0	0.9965	-3.384837	1.400963
1	6	0	-1.677936	-2.591786	0.536248
2	6	0	-1.755781	0.02062	-0.735075
3	6	0	0.245041	0.485187	-2.796167
4	6	0	2.809922	1.506477	-1.806855
5	6	0	4.315851	0.058701	0.25671
6	6	0	2.716118	-1.232141	2.310733
7	6	0	-0.493224	2.700776	-4.578764
8	6	0	0.079092	5.068963	-2.971998
9	6	0	2.26297	4.335111	-1.184736
10	8	0	5.832791	1.842086	1.681973
11	6	0	6.118548	-1.833585	-0.998634
12	8	0	1.53037	4.75027	1.356569
13	6	0	-3.129647	2.656782	-5.75071
14	6	0	-3.696259	-2.775055	2.659707
15	6	0	-3.022527	-1.28206	5.061179
16	8	0	-4.991551	-1.296464	6.877245
17	1	0	4.05903	1.566935	-3.483749
18	1	0	-2.273082	-3.977599	-0.912
19	1	0	0.503731	-1.267644	-3.905937
20	6	0	-4.362533	-5.515874	3.321824
21	1	0	-5.431842	-1.882343	1.887323
22	1	0	0.830256	-4.796328	2.925633
23	1	0	1.938259	-4.400534	-0.155093
24	1	0	-3.650552	0.20688	-1.583766
25	1	0	-1.608977	1.52815	0.689751
26	1	0	1.609551	0.230978	3.286315
27	1	0	4.079813	-1.962769	3.709899
28	1	0	0.87838	2.649002	-6.157167
29	1	0	0.530707	6.729016	-4.148196
30	1	0	-1.564722	5.576443	-1.794044
31	1	0	3.97157	5.475511	-1.596911
32	1	0	7.054437	2.605169	0.54058
33	1	0	5.105679	-3.137844	-2.27071
34	1	0	7.524944	-0.815493	-2.163949
35	1	0	7.140349	-2.952028	0.43568
36	1	0	2.973458	4.189852	2.366636
37	1	0	-4.630058	2.934884	-4.327982
38	1	0	-3.325793	4.189139	-7.156825
39	1	0	-3.497212	0.8481	-6.730873
40	1	0	-2.485399	0.683366	4.576453
41	1	0	-1.387066	-2.152944	6.017056
42	1	0	-6.4635	-0.492235	6.128811
43	1	0	-5.089316	-6.540692	1.65366
44	1	0	-2.695056	-6.555118	4.033396
45	1	0	-5.808692	-5.568106	4.820443

**2_c**			Standard Orientation (A.U.)		
Center number	Atomic number	Atomic Type	X	Y	Z
0	6	0	1.818025	-3.670372	1.559486
1	6	0	-0.841921	-3.050792	0.54816
2	6	0	-0.731796	-2.196132	-2.245676
3	6	0	0.589467	0.265905	-3.187224
4	6	0	2.594124	1.681472	-1.561094
5	6	0	4.547211	0.242473	0.081957
6	6	0	3.50413	-1.423247	2.23718
7	6	0	-1.162975	2.518122	-3.929577
8	6	0	-1.40714	4.078715	-1.494866
9	6	0	1.23506	3.972627	-0.280456
10	8	0	6.141578	2.114555	1.215041
11	6	0	6.279919	-1.300855	-1.645325
12	8	0	1.195251	3.74807	2.411834
13	6	0	-3.650875	1.928337	-5.263237
14	6	0	-2.455221	-1.405306	2.350191
15	6	0	-2.374377	-2.413785	5.079956
16	8	0	-3.88608	-1.001653	6.779633
17	1	0	3.805738	2.608228	-2.979666
18	1	0	-1.833088	-4.895635	0.451805
19	1	0	1.546708	-0.324881	-4.940593
20	6	0	-5.212889	-1.239463	1.472568
21	1	0	-1.661877	0.514582	2.430606

22	1	0	1.631998	-4.854689	3.266986
23	1	0	2.77974	-4.889104	0.161359
24	1	0	0.22402	-3.772522	-3.223546
25	1	0	-2.66349	-2.218657	-3.022794
26	1	0	2.50757	-0.188168	3.579261
27	1	0	5.190518	-2.138123	3.240639
28	1	0	-0.028871	3.664196	-5.2642
29	1	0	-2.054785	6.029943	-1.856297
30	1	0	-2.787886	3.186922	-0.215015
31	1	0	2.308232	5.702581	-0.747474
32	1	0	5.070795	3.018101	2.412839
33	1	0	7.716475	-2.300588	-0.510861
34	1	0	5.213726	-2.70594	-2.756134
35	1	0	7.27251	-0.028697	-2.966618
36	1	0	0.25545	5.162342	3.111823
37	1	0	-4.549093	3.689262	-5.93737
38	1	0	-3.346943	0.697375	-6.922709
39	1	0	-5.023027	0.981455	-4.011258
40	1	0	-0.446637	-2.283734	5.846774
41	1	0	-2.91583	-4.446944	5.096019
42	1	0	-5.650725	-1.322055	6.401317
43	1	0	-6.314669	0.032165	2.706706
44	1	0	-5.387905	-0.489397	-0.458801
45	1	0	-6.120193	-3.126573	1.491836

**2_d**			Standard Orientation (A.U.)		
Center number	Atomic number	Atomic Type	X	Y	Z
0	6	0	1.181423	-2.566571	2.898415
1	6	0	-1.144046	-2.456787	1.13134
2	6	0	-1.627322	0.155465	-0.064658
3	6	0	-0.014957	0.659255	-2.428336
4	6	0	2.799492	1.333695	-1.990723
5	6	0	4.431954	-0.185835	-0.084783
6	6	0	3.215336	-0.537026	2.550296
7	6	0	-0.905675	2.988263	-3.981476
8	6	0	0.295927	5.232253	-2.584964
9	6	0	2.875254	4.278486	-1.628393
10	8	0	6.759634	1.148071	0.238582
11	6	0	5.170467	-2.744043	-1.208611
12	8	0	3.467526	5.156821	0.858885
13	6	0	-3.738248	3.2612	-4.453582
14	6	0	-3.540913	-3.607343	2.380225
15	6	0	-4.526535	-2.219316	4.716237
16	8	0	-5.145968	0.316692	4.120506
17	1	0	3.731714	1.040203	-3.83462
18	1	0	-0.750633	-3.729672	-0.479729
19	1	0	-0.156118	-1.043575	-3.630242
20	6	0	-3.157935	-6.408822	3.034248
21	1	0	-5.063823	-3.494444	0.951367
22	1	0	0.554095	-2.416851	4.884085
23	1	0	2.062571	-4.450191	2.743058
24	1	0	-3.628861	0.222668	-0.625357
25	1	0	-1.431384	1.66661	1.355137
26	1	0	2.479743	1.296277	3.202432
27	1	0	4.798957	-0.990819	3.831711
28	1	0	0.031513	2.832437	-5.845702
29	1	0	0.455644	6.943286	-3.764858
30	1	0	-0.891082	5.74901	-0.940876
31	1	0	4.431293	5.044355	-2.771194
32	1	0	6.305062	2.771687	0.994662
33	1	0	6.35849	-2.436477	-2.893787
34	1	0	6.282174	-3.847488	0.169845
35	1	0	3.499904	-3.857927	-1.765728
36	1	0	2.018369	4.882039	1.952505
37	1	0	-4.791064	3.625177	-2.688909
38	1	0	-4.115528	4.857012	-5.747465
39	1	0	-4.532598	1.537748	-5.32696
40	1	0	-3.097397	-2.300234	6.251728
41	1	0	-6.217624	-3.248373	5.415178
42	1	0	-5.813969	1.120153	5.627355
43	1	0	-2.476736	-7.47095	1.371757

44	1	0	-1.756955	-6.664948	4.561635
45	1	0	-4.942932	-7.29098	3.664298

**2_e**			Standard Orientation (A.U.)		
Center number	Atomic number	Atomic Type	X	Y	Z
0	6	0	0.888031	-1.976937	3.39925
1	6	0	-1.368567	-1.948302	1.560776
2	6	0	-1.258381	0.205073	-0.401671
3	6	0	0.823883	-0.100267	-2.41724
4	6	0	3.472604	0.93884	-1.667692
5	6	0	4.283429	0.803532	1.144399
6	6	0	3.564738	-1.776815	2.307483
7	6	0	0.264414	1.169296	-5.071836
8	6	0	2.436633	3.106251	-5.50638
9	6	0	3.609569	3.569423	-2.914252
10	8	0	3.094281	2.705416	2.634949
11	6	0	7.167469	1.155926	1.322654
12	8	0	2.280562	5.492138	-1.525456
13	6	0	-2.350469	2.372565	-5.411399
14	6	0	-3.926722	-1.882095	3.010693
15	6	0	-4.110328	-3.945665	5.058591
16	8	0	-6.456083	-3.9497	6.353272
17	1	0	4.867732	-0.213783	-2.712024
18	1	0	-1.338684	-3.74673	0.479572
19	1	0	0.999558	-2.149298	-2.743496
20	6	0	-6.236691	-2.083507	1.260205
21	1	0	-4.018091	-0.036409	4.00886
22	1	0	0.654737	-0.42556	4.772511
23	1	0	0.833434	-3.756488	4.483721
24	1	0	-3.08558	0.23539	-1.3777
25	1	0	-1.076531	2.017552	0.610813
26	1	0	4.880062	-2.149644	3.88538
27	1	0	3.914809	-3.279941	0.897799
28	1	0	0.417946	-0.319084	-6.521422
29	1	0	3.88955	2.300261	-6.765817
30	1	0	1.790574	4.889659	-6.373781
31	1	0	5.554964	4.291476	-3.040408
32	1	0	3.005033	4.242286	1.607435
33	1	0	8.197652	-0.408303	0.397077
34	1	0	7.766154	2.948107	0.435657
35	1	0	7.723004	1.226187	3.330605
36	1	0	0.476487	5.153387	-1.614213
37	1	0	-2.494097	3.227076	-7.310582
38	1	0	-3.890867	0.977872	-5.235647
39	1	0	-2.722576	3.890645	-4.02366
40	1	0	-2.542617	-3.767252	6.427558
41	1	0	-3.952723	-5.829523	4.169981
42	1	0	-6.632262	-2.338042	7.217137
43	1	0	-6.018423	-3.683409	-0.069637
44	1	0	-7.948255	-2.433151	2.392853
45	1	0	-6.553887	-0.356444	0.139492

**2_f**			Standard Orientation (A.U.)		
Center number	Atomic number	Atomic Type	X	Y	Z
0	6	0	0.980897	-1.068625	4.260427
1	6	0	-1.297157	-1.700282	2.585461
2	6	0	-1.587934	0.035065	0.268804
3	6	0	0.320718	-0.474305	-1.870022
4	6	0	3.035416	0.669516	-1.590109
5	6	0	4.073377	1.196718	1.100227
6	6	0	3.566505	-1.007736	2.957357
7	6	0	-0.553051	0.576955	-4.476191
8	6	0	0.413934	3.296387	-4.427703
9	6	0	3.12818	3.037533	-3.438486
10	8	0	3.043906	3.523249	2.118039
11	6	0	6.933467	1.645547	0.966191
12	8	0	4.150016	5.294541	-2.472319
13	6	0	-3.336918	0.26043	-5.168763
14	6	0	-3.751479	-1.868406	4.178566
15	6	0	-5.829976	-3.386464	2.830441
16	8	0	-5.035459	-5.796276	1.992717

17	1	0	4.33493	-0.756465	-2.376816
18	1	0	-0.98986	-3.623316	1.83383
19	1	0	0.466893	-2.550628	-2.007985
20	6	0	-4.798108	0.695416	5.03326
21	1	0	-3.262741	-2.954781	5.905512
22	1	0	0.681732	0.751886	5.245619
23	1	0	1.081234	-2.494628	5.784522
24	1	0	-3.48701	-0.29195	-0.512523
25	1	0	-1.595402	2.04935	0.83385
26	1	0	5.011936	-0.899603	4.45833
27	1	0	3.891857	-2.807435	1.946894
28	1	0	0.564699	-0.453069	-5.91669
29	1	0	0.332446	4.262281	-6.274055
30	1	0	-0.700591	4.440009	-3.074786
31	1	0	4.352672	2.531639	-5.04915
32	1	0	1.247073	3.297842	2.402983
33	1	0	7.378844	3.129293	-0.420397
34	1	0	7.631752	2.24276	2.838527
35	1	0	7.908495	-0.116485	0.422349
36	1	0	3.640142	5.408619	-0.695532
37	1	0	-3.968315	-1.717133	-4.927777
38	1	0	-4.577338	1.477381	-4.012113
39	1	0	-3.649583	0.787781	-7.1646
40	1	0	-6.472692	-2.383646	1.118945
41	1	0	-7.494294	-3.535518	4.102589
42	1	0	-4.485217	-6.766125	3.452736
43	1	0	-5.449387	1.834802	3.408795
44	1	0	-6.430069	0.445922	6.312178
45	1	0	-3.369608	1.812367	6.064257

Table S9. Gibbs free energies[a] and equilibrium populations[b] of low-energy conformers of compound **5**.

**Conformers**	**ΔG(a.u.)**	**P(%)/100**	**G(a.u.)**
**5_a**	0.00348	1.29	-888.977565
**5_b**	0.00949	0.0	-888.971558
**5_c**	0.01665	0.0	-888.964394
**5_d**	0.0	51.35	-888.981043
**5_e**	0.00354	1.21	-888.977502
**5_f**	0.004	0.74	-888.977044
5_**g**	0.00018	42.27	-888.980859
5_**h**	0.00264	3.14	-888.978405

[a]wB97M-V/def2-TZVP, in a.u. [b]From ΔG values at 298.15K.

Table S10. Cartesian coordinates for the low-energy reoptimized random reseach conformers of compound **5** at B3LYP-D3(BJ)/6-31G* level of theory in methanol.

5_a			**Standard Orientation (A.U.)**		
**Center number**	**Atomic number**	**Atomic Type**	**X**	Y	Z
0	6	0	3.699469	0.641288	-0.746003
1	6	0	1.768089	2.454823	-1.966636
2	6	0	-0.943302	1.397064	-2.279731
3	6	0	-1.955092	-0.418293	-0.26766
4	6	0	-1.088262	-3.183682	-0.639001
5	6	0	1.645903	-3.79879	0.097068
6	6	0	3.536922	-2.162656	-1.451177
7	6	0	-4.865208	-0.663891	-0.274873
8	6	0	-5.295831	-3.021901	1.364356
9	6	0	-3.134867	-4.850354	0.66112
10	1	0	-1.182159	-3.575632	-2.687197
11	1	0	-1.411399	0.270973	1.622853
12	6	0	-6.337484	1.656869	0.59901
13	6	0	2.138385	-3.573278	2.955625
14	8	0	2.062013	-6.34876	-0.659493
15	8	0	5.992326	-3.301519	-1.273811
16	6	0	1.90386	5.136652	-0.731963
17	6	0	4.580262	6.235096	-0.948932
18	6	0	1.051808	5.291951	2.048626
19	8	0	0.167522	6.799777	-1.990585

20	8	0	-1.556512	4.922214	2.361318
21	1	0	2.454133	2.787412	-3.918845
22	1	0	3.623876	0.810618	1.329899
23	1	0	5.606409	1.256596	-1.314449
24	1	0	-2.221987	3.026188	-2.465673
25	1	0	-1.039253	0.400307	-4.113644
26	1	0	3.035547	-2.376105	-3.461135
27	1	0	-5.445327	-1.118442	-2.238888
28	1	0	-7.191558	-3.850615	1.097374
29	1	0	-5.150632	-2.464834	3.374384
30	1	0	-2.401041	-5.848856	2.330553
31	1	0	-3.779894	-6.328571	-0.65516
32	1	0	-8.388101	1.257785	0.633058
33	1	0	-6.046946	3.285414	-0.67621
34	1	0	-5.74616	2.240815	2.512291
35	1	0	1.13777	-5.100407	3.951247
36	1	0	4.170306	-3.814645	3.379923
37	1	0	1.517064	-1.753247	3.75823
38	1	0	3.890072	-6.507096	-0.859103
39	1	0	6.73296	-2.830435	0.34084
40	1	0	4.53376	8.261296	-0.464863
41	1	0	5.309011	6.045068	-2.897923
42	1	0	5.9249	5.287337	0.33316
43	1	0	2.02861	3.854365	3.195951
44	1	0	1.630603	7.172151	2.7802
45	1	0	0.384037	6.629889	-3.806237
46	1	0	-2.379291	5.881796	1.02139
	**5_b**		Standard Orientation (A.U.)		
Center number	Atomic number	Atomic Type	X	Y	Z
0	6	0	3.131929	0.864815	-1.553838
1	6	0	0.552609	2.188494	-1.000073
2	6	0	-0.88175	1.135844	1.304431
3	6	0	-1.998958	-1.512508	0.863139
4	6	0	-0.566698	-3.207296	-1.013526
5	6	0	2.200464	-3.861703	-0.412441
6	6	0	3.88123	-1.468347	-0.016207
7	6	0	-4.716812	-1.508056	-0.223274
8	6	0	-5.032282	-4.12709	-1.532303
9	6	0	-2.415707	-5.420855	-1.388392
10	1	0	-0.493922	-2.19444	-2.835648
11	1	0	-2.065232	-2.511249	2.701735
12	6	0	-6.748572	-0.935457	1.743931
13	6	0	2.49262	-5.672849	1.830159
14	8	0	3.303958	-5.002738	-2.615
15	8	0	4.13016	-0.721606	2.559012
16	6	0	0.820938	5.10355	-0.88323
17	6	0	1.985454	6.168882	-3.322575
18	6	0	2.321938	5.98263	1.441722
19	8	0	-1.679144	6.086933	-0.64152
20	8	0	2.075894	8.664291	1.549909
21	1	0	-0.683769	1.893159	-2.655464
22	1	0	4.693223	2.209336	-1.259215
23	1	0	3.228139	0.321189	-3.562664
24	1	0	0.389009	1.068732	2.939354
25	1	0	-2.422887	2.460035	1.75079
26	1	0	5.752772	-2.066752	-0.733866
27	1	0	-4.792954	-0.015782	-1.684319
28	1	0	-5.636564	-3.891221	-3.513766
29	1	0	-6.496775	-5.280541	-0.596914
30	1	0	-2.348658	-6.701742	0.25649
31	1	0	-2.005141	-6.570034	-3.083871
32	1	0	-8.650795	-0.887033	0.882033
33	1	0	-6.423574	0.914102	2.656796
34	1	0	-6.762755	-2.396114	3.241087
35	1	0	1.614371	-7.514454	1.400975
36	1	0	4.516468	-6.043315	2.195341
37	1	0	1.6097	-4.924539	3.563403
38	1	0	2.179293	-6.336868	-3.183763
39	1	0	5.061004	-2.020124	3.45648
40	1	0	3.923727	5.477851	-3.656831
41	1	0	2.059112	8.248977	-3.216366

42	1	0	0.792945	5.630632	-4.946784
43	1	0	1.512809	5.105283	3.154333
44	1	0	4.330315	5.416344	1.304588
45	1	0	-1.467549	7.82574	-0.067313
46	1	0	2.631344	9.250815	3.193663

**5_c**			Standard Orientation (A.U.)		
Center number	Atomic number	Atomic Type	X	Y	Z
0	6	0	3.066514	0.984322	-2.068045
1	6	0	0.654612	2.672417	-2.091579
2	6	0	-1.932444	1.333732	-1.634926
3	6	0	-1.970845	-1.157174	-0.170817
4	6	0	-0.456512	-3.26491	-1.541485
5	6	0	2.250477	-3.603967	-0.496751
6	6	0	3.553163	-1.021745	-0.000915
7	6	0	-4.659947	-2.231523	0.210252
8	6	0	-4.181421	-5.076445	0.53455
9	6	0	-2.18339	-5.644988	-1.48847
10	1	0	-0.188107	-2.701036	-3.528096
11	1	0	-1.144146	-0.855223	1.713243
12	6	0	-6.133195	-0.994697	2.358962
13	6	0	2.388472	-5.221082	1.903593
14	8	0	3.784757	-4.742484	-2.423609
15	8	0	2.956569	-0.243187	2.473669
16	6	0	0.834976	5.258407	-0.652997
17	6	0	3.082451	6.851236	-1.575947
18	6	0	0.842021	5.118588	2.257593
19	8	0	-1.424746	6.665863	-1.155781
20	8	0	3.260622	4.638627	3.330504
21	1	0	0.583487	3.331797	-4.078372
22	1	0	4.719019	2.25212	-2.108436
23	1	0	3.164838	-0.049757	-3.875266
24	1	0	-3.209319	2.702535	-0.720746
25	1	0	-2.789037	0.937336	-3.500972
26	1	0	5.595568	-1.478378	-0.134228
27	1	0	-5.728732	-1.966202	-1.573706
28	1	0	-5.909996	-6.2293	0.335012
29	1	0	-3.403887	-5.434666	2.442215
30	1	0	-1.160141	-7.428422	-1.144751
31	1	0	-3.127593	-5.851911	-3.33786
32	1	0	-6.379535	1.054111	2.035321
33	1	0	-5.12842	-1.234551	4.176723
34	1	0	-8.035429	-1.833487	2.564759
35	1	0	1.150108	-4.4926	3.406937
36	1	0	1.851335	-7.193455	1.485597
37	1	0	4.340909	-5.230894	2.630666
38	1	0	2.94753	-6.286676	-2.957179
39	1	0	3.623991	1.453997	2.805072
40	1	0	2.996609	7.083075	-3.649258
41	1	0	4.934088	6.011077	-1.124057
42	1	0	2.975663	8.755939	-0.726465
43	1	0	0.082203	6.931712	2.963678
44	1	0	-0.399415	3.593878	2.927064
45	1	0	-1.771579	6.573404	-2.957766
46	1	0	4.370802	6.066318	3.028382

**5_d**			Standard Orientation (A.U.)		
Center number	Atomic number	Atomic Type	X	Y	Z
0	6	0	3.102627	0.955056	0.590152
1	6	0	1.129208	2.458071	-0.943328
2	6	0	-1.644799	1.699074	-0.42049
3	6	0	-2.11676	-1.066623	0.29323
4	6	0	-0.693741	-3.088292	-1.29423
5	6	0	1.948072	-3.782446	-0.265903
6	6	0	3.823468	-1.57252	-0.607729
7	6	0	-4.92677	-1.840377	0.137662
8	6	0	-4.793485	-4.733975	0.23136
9	6	0	-2.541688	-5.380217	-1.48411
10	1	0	-0.377711	-2.347333	-3.217052
11	1	0	-1.571423	-1.320334	2.290189
12	6	0	-6.624159	-0.648314	2.140876
13	6	0	1.920782	-4.759553	2.462155
14	8	0	3.062304	-5.698025	-1.852573

15	8	0	6.232252	-2.278732	0.32071
16	6	0	1.506849	5.358197	-0.705556
17	6	0	3.89628	6.241475	-2.102964
18	6	0	1.603403	6.213923	2.072811
19	8	0	-0.633826	6.525963	-1.844068
20	8	0	1.422733	8.897595	2.058699
21	1	0	1.499383	2.042007	-2.954544
22	1	0	2.495869	0.69248	2.569473
23	1	0	4.894166	2.010389	0.675203
24	1	0	-2.419698	2.89271	1.102781
25	1	0	-2.76533	2.18325	-2.109233
26	1	0	3.945869	-1.275093	-2.680115
27	1	0	-5.633764	-1.2849	-1.755083
28	1	0	-6.56687	-5.657325	-0.365638
29	1	0	-4.40829	-5.337864	2.19721
30	1	0	-1.662719	-7.19795	-0.957529
31	1	0	-3.182864	-5.601819	-3.454613
32	1	0	-6.678209	1.431021	1.965969
33	1	0	-5.939285	-1.113425	4.062296
34	1	0	-8.586464	-1.346316	1.9794
35	1	0	1.30577	-3.312979	3.826155
36	1	0	0.635324	-6.395284	2.654917
37	1	0	3.840834	-5.349318	3.000322
38	1	0	2.215227	-7.292519	-1.529278
39	1	0	6.570119	-3.935331	-0.411113
40	1	0	4.121657	8.302002	-1.878124
41	1	0	3.701828	5.814283	-4.134164
42	1	0	5.628176	5.325663	-1.387238
43	1	0	-0.001116	5.376552	3.121664
44	1	0	3.382985	5.584778	2.973283
45	1	0	-0.630162	8.265199	-1.234008
46	1	0	1.153831	9.498628	3.767997
**5_e**			Standard Orientation (A.U.)		
Center number	Atomic number	Atomic Type	X	Y	Z
0	6	0	2.869227	1.123008	0.290773
1	6	0	0.956568	2.363618	-1.533701
2	6	0	-1.85609	1.767177	-0.966131
3	6	0	-2.410231	-0.726433	0.399014
4	6	0	-1.058054	-3.068297	-0.742831
5	6	0	1.526377	-3.673562	0.429261
6	6	0	3.483481	-1.63966	-0.33285
7	6	0	-5.295695	-1.374665	0.405402
8	6	0	-5.499662	-3.834748	-1.15564
9	6	0	-3.012782	-5.216924	-0.621958
10	1	0	-0.707991	-2.704194	-2.771785
11	1	0	-1.810315	-0.504348	2.378212
12	6	0	-6.330576	-1.686478	3.092053
13	6	0	1.447094	-4.033032	3.315613
14	8	0	2.340026	-5.987763	-0.691863
15	8	0	5.912954	-2.3476	0.619491
16	6	0	1.365859	5.267506	-1.847612
17	6	0	3.849726	5.851381	-3.231146
18	6	0	1.237819	6.748128	0.663825
19	8	0	-0.67056	6.321717	-3.277374
20	8	0	3.354908	6.425181	2.255569
21	1	0	1.378871	1.56158	-3.420927
22	1	0	2.255713	1.343746	2.271735
23	1	0	4.684503	2.124229	0.161574
24	1	0	-2.692532	3.310119	0.159257
25	1	0	-2.905231	1.783523	-2.77031
26	1	0	3.696385	-1.80827	-2.399413
27	1	0	-6.369201	0.158346	-0.517804
28	1	0	-5.610705	-3.374653	-3.191245
29	1	0	-7.196874	-4.953052	-0.681443
30	1	0	-3.060363	-6.071786	1.280966
31	1	0	-2.582208	-6.74569	-1.965669
32	1	0	-6.056036	0.059841	4.206071
33	1	0	-5.362222	-3.235669	4.106953
34	1	0	-8.374402	-2.118114	3.071868
35	1	0	3.271156	-4.803185	3.970185
36	1	0	1.076374	-2.268888	4.363111

37	1	0	-0.017675	-5.417907	3.834625
38	1	0	4.14799	-6.097343	-0.350388
39	1	0	5.984092	-1.90986	2.40256
40	1	0	5.534314	5.143134	-2.228922
41	1	0	4.040242	7.91342	-3.498543
42	1	0	3.841184	4.963995	-5.122425
43	1	0	0.933401	8.759989	0.165997
44	1	0	-0.41838	6.135399	1.76554
45	1	0	-0.816151	5.387603	-4.851716
46	1	0	4.800503	7.261349	1.497103
**5_f**			Standard Orientation (A.U.)		
Center number	Atomic number	Atomic Type	X	Y	Z
0	6	0	3.535278	0.859307	-1.066386
1	6	0	1.382529	2.44449	-2.240873
2	6	0	-1.207603	1.100887	-2.331028
3	6	0	-2.020241	-0.515142	-0.064005
4	6	0	-0.957433	-3.226292	-0.064857
5	6	0	1.889597	-3.578929	0.36615
6	6	0	3.468676	-1.995279	-1.546049
7	6	0	-4.9238	-0.943088	-0.020213
8	6	0	-5.336606	-3.535509	1.309918
9	6	0	-2.675136	-4.601747	1.829638
10	1	0	-1.323074	-4.037669	-1.957722
11	1	0	-1.453655	0.416029	1.714615
12	6	0	-6.401434	1.230138	1.175938
13	6	0	2.706854	-3.034804	3.104215
14	8	0	2.389979	-6.166113	-0.194932
15	8	0	5.989608	-2.998561	-1.583517
16	6	0	1.241861	5.16996	-1.111264
17	6	0	3.850062	6.403371	-0.98525
18	6	0	-0.073059	5.348809	1.477984
19	8	0	-0.18972	6.747187	-2.775233
20	8	0	1.20743	3.89991	3.33476
21	1	0	1.900136	2.79781	-4.232095
22	1	0	3.617041	1.209212	0.979077
23	1	0	5.330239	1.542228	-1.873566
24	1	0	-2.685609	2.536032	-2.697365
25	1	0	-1.263593	-0.124707	-4.020484
26	1	0	2.743051	-2.412286	-3.453286
27	1	0	-5.540288	-1.123385	-2.009691
28	1	0	-6.43124	-4.814562	0.080126
29	1	0	-6.434027	-3.323816	3.072013
30	1	0	-2.099927	-4.114046	3.773468
31	1	0	-2.545739	-6.670711	1.648035
32	1	0	-6.066426	3.037053	0.180775
33	1	0	-5.848992	1.491384	3.175746
34	1	0	-8.458166	0.869081	1.128753
35	1	0	2.004471	-4.549965	4.344242
36	1	0	4.790313	-3.049247	3.261132
37	1	0	2.021152	-1.199433	3.815161
38	1	0	4.185185	-6.226622	-0.621817
39	1	0	6.912179	-2.286135	-0.162879
40	1	0	5.016879	5.560595	0.515835
41	1	0	3.617882	8.436785	-0.586331
42	1	0	4.836159	6.212014	-2.813029
43	1	0	-0.11499	7.375123	2.001598
44	1	0	-2.058836	4.713162	1.279993
45	1	0	-1.831805	5.971458	-3.04678
46	1	0	0.366341	4.126507	4.948708
**5_G**			Standard Orientation (A.U.)		
Center number	Atomic number	Atomic Type	X	Y	Z
0	6	0	3.117522	1.087177	0.25631
1	6	0	1.064396	2.352071	-1.39171
2	6	0	-1.681144	1.666959	-0.650143
3	6	0	-2.067343	-0.93715	0.545109
4	6	0	-0.754124	-3.184278	-0.813516
5	6	0	1.920674	-3.75838	0.17507
6	6	0	3.778846	-1.617977	-0.546793
7	6	0	-4.869994	-1.723803	0.76655
8	6	0	-4.687615	-4.576207	1.256189
9	6	0	-2.607361	-5.459531	-0.563943

10	1	0	-0.527638	-2.734889	-2.837621
11	1	0	-1.350095	-0.850842	2.50081
12	6	0	-6.383678	-0.251105	2.729819
13	6	0	1.995033	-4.322184	3.030036
14	8	0	2.75519	-5.959534	-1.141431
15	8	0	6.268384	-2.302804	0.249761
16	6	0	1.435078	5.265799	-1.562887
17	6	0	3.707753	5.974827	-3.21027
18	6	0	1.654458	6.518518	1.057638
19	8	0	-0.784683	6.427759	-2.617277
20	8	0	1.540763	9.166094	0.865244
21	1	0	1.372466	1.641287	-3.333949
22	1	0	2.597337	1.163846	2.277379
23	1	0	4.900745	2.146467	0.067014
24	1	0	-2.426519	3.102782	0.662136
25	1	0	-2.874348	1.816189	-2.355167
26	1	0	3.899649	-1.656098	-2.626384
27	1	0	-5.759164	-1.449258	-1.111531
28	1	0	-6.50188	-5.568505	0.976962
29	1	0	-4.111165	-4.890557	3.243111
30	1	0	-1.640314	-7.194594	0.050926
31	1	0	-3.436987	-5.882136	-2.428938
32	1	0	-6.472036	1.784563	2.274069
33	1	0	-5.520255	-0.444244	4.625249
34	1	0	-8.346423	-0.954583	2.85259
35	1	0	3.85947	-5.10204	3.545285
36	1	0	1.641925	-2.645613	4.218626
37	1	0	0.576059	-5.767359	3.513907
38	1	0	4.580319	-6.027164	-0.892933
39	1	0	6.393667	-2.038593	2.0637
40	1	0	5.472125	5.091862	-2.53904
41	1	0	3.96453	8.04137	-3.184496
42	1	0	3.402239	5.350898	-5.183288
43	1	0	0.137383	5.782955	2.305983
44	1	0	3.482393	6.035198	1.929658
45	1	0	-0.837482	6.084204	-4.419434
46	1	0	0.157538	9.509786	-0.304884

**5_h**			Standard Orientation (A.U.)		
Center number	Atomic number	Atomic Type	X	Y	Z
0	6	0	3.457619	0.971606	-0.106925
1	6	0	0.861474	2.04747	-0.897477
2	6	0	-1.203043	1.401563	1.051564
3	6	0	-2.218614	-1.331934	0.993668
4	6	0	-0.615668	-3.376284	-0.318162
5	6	0	2.091082	-3.771406	0.646175
6	6	0	3.883459	-1.83692	-0.629373
7	6	0	-4.803505	-1.669652	-0.323974
8	6	0	-5.033249	-4.577154	-0.717565
9	6	0	-2.370407	-5.687443	-0.205162
10	1	0	-0.454963	-2.867098	-2.339375
11	1	0	-2.490275	-1.94828	2.972714
12	6	0	-7.030722	-0.506105	1.094246
13	6	0	2.291066	-3.70763	3.541193
14	8	0	2.885479	-6.191562	-0.2317
15	8	0	6.446438	-2.514459	-0.103185
16	6	0	0.987414	4.935844	-1.462259
17	6	0	2.557107	5.508314	-3.82803
18	6	0	1.949868	6.483429	0.80526
19	8	0	-1.518465	5.914059	-1.860531
20	8	0	1.706192	9.093141	0.361425
21	1	0	0.332819	1.176223	-2.724856
22	1	0	3.761514	1.351805	1.928635
23	1	0	4.993416	1.950513	-1.117049
24	1	0	-0.450368	1.815191	2.952836
25	1	0	-2.789121	2.717528	0.773799
26	1	0	3.689045	-2.165259	-2.678471
27	1	0	-4.667512	-0.743015	-2.197624
28	1	0	-5.683204	-5.012704	-2.650404
29	1	0	-6.45158	-5.386715	0.580997
30	1	0	-2.286724	-6.544416	1.695268
31	1	0	-1.804029	-7.158678	-1.558615

32	1	0	-6.79584	1.552858	1.356519
33	1	0	-7.235494	-1.371982	2.986932
34	1	0	-8.821824	-0.808126	0.062379
35	1	0	4.238319	-4.172359	4.134447
36	1	0	1.782497	-1.85449	4.347997
37	1	0	1.03893	-5.152232	4.369108
38	1	0	4.729222	-6.151324	-0.183013
39	1	0	6.874029	-1.913641	1.580209
40	1	0	4.517853	4.824603	-3.665082
41	1	0	2.61781	7.566941	-4.131953
42	1	0	1.705972	4.594872	-5.506941
43	1	0	0.896816	5.907835	2.524545
44	1	0	3.969891	6.092841	1.130179
45	1	0	-2.117659	5.361128	-3.505982
46	1	0	0.006977	9.31646	-0.317875

Table S11. Gibbs free energies[a] and equilibrium populations[b] of low-energy conformers of compound **7**.

Conformers	ΔG(a.u.)	P(%)/100	G(a.u.)
**7_a**	0.00726	0.02	-812.530382
**7_b**	0.0	52.84	-812.537641
**7_c**	0.0042	0.62	-812.533444
**7_d**	0.0032	1.78	-812.534442
**7_e**	0.00381	0.94	-812.533832
**7_f**	0.00725	0.02	-812.53039
**7_g**	0.00757	0.02	-812.530067
**7_h**	0.0003	38.58	-812.537344
**7_i**	0.00468	0.37	-812.532966
**7_j**	0.00333	1.56	-812.534316
**7_k**	0.00494	0.28	-812.532705
**7_l**	0.006	0.09	-812.531639
**7_m**	0.00724	0.03	-812.530398
**7_n**	0.00324	1.72	-812.534405
**7_o**	0.00448	0.46	-812.533162
**7_p**	0.00528	0.2	-812.532358
**7_q**	0.00459	0.41	-812.533056
**7_r**	0.00646	0.06	-812.531183

[a]wB97M-V/def2-TZVP, in a.u. [b]From ΔG values at 298.15K.

Table S12. Cartesian coordinates for the low-energy reoptimized random reseach conformers of compound **7** at B3LYP-D3(BJ)/6-31G* level of theory in methanol.

7_a			Standard Orientation (A.U.)		
Center number	Atomic number	Atomic Type	X	Y	Z
0	6	0	1.136158	0.534212	2.967955
1	6	0	3.58578	1.652329	1.90576
2	6	0	4.05392	1.559806	-0.977049
3	6	0	1.666924	1.398074	-2.648644
4	6	0	-0.130501	-0.967768	-2.333009
5	6	0	0.619817	-3.001278	-0.403628
6	6	0	0.640192	-2.272517	2.452027
7	6	0	-0.091077	3.726914	-2.546431
8	6	0	-2.677266	2.698803	-3.363123
9	6	0	-2.832253	0.111634	-2.057451
10	6	0	-4.925545	-1.593774	-3.059179
11	8	0	-4.755256	4.327802	-2.881984
12	6	0	5.643227	3.865357	-1.749165
13	8	0	5.586369	-0.663734	-1.383295
14	6	0	-1.717376	-3.245151	3.783368
15	6	0	-1.786477	-6.072951	4.209977
16	6	0	-3.603216	-1.815054	4.670146
17	8	0	0.217719	-6.890739	5.825107
18	1	0	-0.090958	-1.947845	-4.163656
19	1	0	2.409516	1.306266	-4.589162
20	1	0	2.209073	-3.334078	3.297869
21	1	0	-0.454695	1.667026	2.295403
22	1	0	1.170111	0.806635	5.023748
23	1	0	5.221873	0.727134	2.775809

24	1	0	3.654511	3.63342	2.509598
25	1	0	2.504518	-3.664124	-0.905447
26	1	0	-0.659892	-4.607485	-0.679812
27	1	0	0.551335	5.288085	-3.738292
28	1	0	-0.243661	4.463412	-0.61126
29	1	0	-2.693508	2.428676	-5.42039
30	1	0	-3.223712	0.477437	-0.053736
31	1	0	-4.581361	-2.104739	-5.039631
32	1	0	-5.063323	-3.342704	-1.959997
33	1	0	-6.751559	-0.624464	-2.959576
34	1	0	-4.848652	4.552484	-1.063205
35	1	0	4.631675	5.642688	-1.437114
36	1	0	6.150613	3.766855	-3.757898
37	1	0	7.392799	3.901931	-0.645876
38	1	0	5.886263	-0.788619	-3.189355
39	1	0	-3.639325	-6.634553	4.967487
40	1	0	-1.492878	-7.107585	2.442639
41	1	0	-5.223575	-2.661912	5.608548
42	1	0	-3.628628	0.229201	4.502932
43	1	0	0.050673	-5.943533	7.386885

**7_b**			Standard Orientation (A.U.)		
Center number	Atomic number	Atomic Type	X	Y	Z
0	6	0	0.901526	0.684223	4.710762
1	6	0	2.980503	2.112956	3.298678
2	6	0	2.407808	3.112088	0.629844
3	6	0	2.387537	0.956846	-1.357383
4	6	0	0.18847	-1.03739	-1.343121
5	6	0	-1.429306	-1.421526	1.022514
6	6	0	0.053835	-1.822346	3.51384
7	6	0	2.435524	1.970272	-4.118798
8	6	0	0.643499	0.269379	-5.640296
9	6	0	-1.384381	-0.462208	-3.723838
10	6	0	-3.117222	-2.585198	-4.613392
11	8	0	-0.487445	1.472822	-7.76175
12	6	0	0.067909	4.823885	0.60342
13	8	0	4.597859	4.655818	0.108285
14	6	0	-1.51992	-3.434154	5.297995
15	6	0	-1.120506	-6.258325	5.090251
16	6	0	-3.177091	-2.494408	6.952474
17	8	0	-1.391243	-7.232012	2.583412
18	1	0	1.114269	-2.871388	-1.695419
19	1	0	4.161657	-0.04846	-1.006512
20	1	0	1.779434	-2.903345	3.058977
21	1	0	-0.750773	1.913057	4.951345
22	1	0	1.60032	0.293527	6.621782
23	1	0	4.675986	0.92357	3.16815
24	1	0	3.523816	3.761974	4.428972
25	1	0	-2.607098	-3.087009	0.690045
26	1	0	-2.752524	0.148767	1.294256
27	1	0	4.35015	2.035913	-4.902069
28	1	0	1.649039	3.884901	-4.241878
29	1	0	1.639031	-1.459995	-6.246996
30	1	0	-2.519496	1.246237	-3.377043
31	1	0	-4.63969	-2.955817	-3.262465
32	1	0	-3.972737	-2.092853	-6.430919
33	1	0	-2.049869	-4.347281	-4.861744
34	1	0	0.821747	1.756119	-9.010655
35	1	0	-0.189447	5.715053	-1.247015
36	1	0	0.317674	6.332425	1.996968
37	1	0	-1.674547	3.812179	1.045287
38	1	0	4.343519	5.418752	-1.538634
39	1	0	0.768036	-6.736333	5.834828
40	1	0	-2.511505	-7.267601	6.23699
41	1	0	-4.325723	-3.729322	8.125684
42	1	0	-3.45341	-0.478475	7.217053
43	1	0	0.052532	-6.665741	1.608873

**7_C**			Standard Orientation (A.U.)		
Center number	Atomic number	Atomic Type	X	Y	Z
0	6	0	1.188632	0.466549	4.032789

1	6	0	2.110019	2.48218	2.164602
2	6	0	3.508282	1.510683	-0.206475
3	6	0	1.708811	0.77492	-2.387599
4	6	0	-0.355318	-1.257707	-1.907106
5	6	0	-1.901573	-1.053258	0.553769
6	6	0	-0.38941	-1.75191	2.927061
7	6	0	0.272589	3.013062	-3.620959
8	6	0	-2.243564	1.950156	-4.634531
9	6	0	-2.070601	-0.943742	-4.258511
10	6	0	-1.00682	-2.254224	-6.614703
11	8	0	-4.220715	3.074161	-3.163933
12	6	0	5.356119	3.535489	-1.133679
13	8	0	5.108837	-0.597598	0.414477
14	6	0	-1.947796	-2.906227	5.041542
15	6	0	-0.436194	-4.131217	7.131123
16	6	0	-4.46204	-2.781036	5.213686
17	8	0	1.438871	-5.74235	6.044625
18	1	0	0.507987	-3.147074	-1.922539
19	1	0	2.970324	-0.025209	-3.824031
20	1	0	0.931684	-3.258699	2.380788
21	1	0	0.040094	1.436742	5.460596
22	1	0	2.822213	-0.324655	5.027441
23	1	0	3.387287	3.73757	3.208551
24	1	0	0.515963	3.660299	1.56745
25	1	0	-3.467411	-2.396158	0.362586
26	1	0	-2.76792	0.817029	0.720776
27	1	0	1.397226	3.890479	-5.116152
28	1	0	-0.184553	4.49892	-2.258761
29	1	0	-2.530219	2.420178	-6.640088
30	1	0	-3.960838	-1.721329	-3.889323
31	1	0	-0.780612	-4.286298	-6.290741
32	1	0	-2.272241	-2.003321	-8.235714
33	1	0	0.843914	-1.496561	-7.151181
34	1	0	-5.795594	2.279369	-3.662497
35	1	0	6.829782	3.816009	0.289858
36	1	0	4.396223	5.339467	-1.450474
37	1	0	6.263919	2.9458	-2.897474
38	1	0	4.033911	-2.003168	0.88662
39	1	0	0.474089	-2.648546	8.27663
40	1	0	-1.70726	-5.194353	8.385584
41	1	0	-5.465167	-3.637167	6.789098
42	1	0	-5.614626	-1.809893	3.823294
43	1	0	2.603633	-6.216426	7.374293
**7_d**			Standard Orientation (A.U.)		
Center number	Atomic number	Atomic Type	X	Y	Z
0	6	0	3.032459	-0.451013	3.169789
1	6	0	3.425818	1.96585	1.614134
2	6	0	3.649107	1.672321	-1.282495
3	6	0	1.074462	1.548024	-2.655986
4	6	0	-0.7922	-0.598178	-1.942145
5	6	0	-1.307966	-0.952685	0.899251
6	6	0	0.867065	-2.248048	2.361751
7	6	0	-0.570873	3.947965	-2.518406
8	6	0	-3.288847	3.122725	-3.239973
9	6	0	-3.217756	0.185596	-3.386792
10	6	0	-3.222442	-0.756497	-6.126709
11	8	0	-5.160903	4.069491	-1.547761
12	6	0	5.200358	3.870395	-2.345521
13	8	0	5.110755	-0.536854	-1.902198
14	6	0	-0.202016	-3.740675	4.577315
15	6	0	-0.461363	-6.548171	4.097253
16	6	0	-0.976845	-2.697166	6.737883
17	8	0	-1.375921	-7.983566	6.156911
18	1	0	-0.109527	-2.422907	-2.665258
19	1	0	1.571179	1.241091	-4.646408
20	1	0	1.666686	-3.653185	1.048048
21	1	0	2.715228	0.140042	5.125206
22	1	0	4.797297	-1.532808	3.186471
23	1	0	5.173282	2.838635	2.306687
24	1	0	1.919886	3.323057	2.036783

25	1	0	-3.001853	-2.132271	1.081915
26	1	0	-1.774857	0.845857	1.824929
27	1	0	0.138912	5.458965	-3.736185
28	1	0	-0.623853	4.722826	-0.597703
29	1	0	-3.839238	3.91138	-5.06981
30	1	0	-4.910625	-0.558214	-2.45079
31	1	0	-3.155633	-2.824838	-6.204736
32	1	0	-4.93457	-0.135123	-7.111478
33	1	0	-1.595326	-0.035162	-7.187012
34	1	0	-4.821799	3.347741	0.103289
35	1	0	4.41496	5.691041	-1.757957
36	1	0	5.247492	3.802811	-4.412877
37	1	0	7.142539	3.736792	-1.647862
38	1	0	4.078953	-2.005606	-1.541907
39	1	0	-1.803662	-6.869686	2.54682
40	1	0	1.376739	-7.276462	3.424596
41	1	0	-1.872932	-3.829978	8.193709
42	1	0	-0.795261	-0.686844	7.110147
43	1	0	-0.184031	-7.765809	7.533478
	**7_e**		Standard Orientation (A.U.)		
Center number	Atomic number	Atomic Type	X	Y	Z
0	6	0	0.411193	0.450463	4.289338
1	6	0	1.067646	2.831003	2.768396
2	6	0	3.033755	2.528229	0.631463
3	6	0	1.893124	1.58212	-1.885892
4	6	0	0.484887	-0.98756	-1.916137
5	6	0	-1.515392	-1.389158	0.16301
6	6	0	-0.376757	-1.947986	2.775683
7	6	0	0.039403	3.406205	-3.21015
8	6	0	-1.564096	1.778051	-5.049652
9	6	0	-0.719615	-0.998129	-4.585498
10	6	0	1.121328	-1.918337	-6.625253
11	8	0	-4.217816	2.152793	-4.741882
12	6	0	4.36104	5.060753	0.191631
13	8	0	5.032048	0.857739	1.40799
14	6	0	-2.027563	-3.58481	4.458934
15	6	0	-0.644901	-4.939549	6.562554
16	6	0	-4.526663	-3.840271	4.204964
17	8	0	1.340902	-6.515968	5.632559
18	1	0	1.840839	-2.552797	-1.75391
19	1	0	3.535834	1.349523	-3.129494
20	1	0	1.334542	-3.07981	2.444869
21	1	0	-1.142264	0.953219	5.565801
22	1	0	2.02378	-0.029236	5.49336
23	1	0	1.795238	4.219541	4.124184
24	1	0	-0.657725	3.666862	1.985441
25	1	0	-2.670419	-3.010251	-0.407858
26	1	0	-2.811854	0.229271	0.280299
27	1	0	1.025464	4.941381	-4.180157
28	1	0	-1.248941	4.304382	-1.859651
29	1	0	-1.220686	2.313597	-7.016226
30	1	0	-2.397853	-2.212841	-4.639334
31	1	0	2.815562	-0.730867	-6.726308
32	1	0	1.738758	-3.859046	-6.24987
33	1	0	0.223288	-1.882938	-8.490677
34	1	0	-4.647908	1.643149	-3.034258
35	1	0	2.99322	6.569682	-0.168053
36	1	0	5.655741	4.939306	-1.417667
37	1	0	5.460594	5.555933	1.871521
38	1	0	4.341933	-0.834975	1.518306
39	1	0	0.298316	-3.584814	7.811667
40	1	0	-1.990089	-6.023716	7.717774
41	1	0	-5.61246	-5.00966	5.499477
42	1	0	-5.593077	-2.886708	2.733946
43	1	0	0.560837	-7.744064	4.51554
	**7_f**		Standard Orientation (A.U.)		
Center number	Atomic number	Atomic Type	X	Y	Z
0	6	0	-0.053564	0.844798	2.994473
1	6	0	2.288205	2.494633	2.578635

2	6	0	3.678671	2.363855	0.01148
3	6	0	2.076433	1.478926	-2.262252
4	6	0	0.869886	-1.249944	-2.173501
5	6	0	1.395833	-2.895212	0.158631
6	6	0	0.301723	-2.020074	2.750876
7	6	0	-0.103903	3.270411	-3.01423
8	6	0	-1.974433	1.568807	-4.402306
9	6	0	-1.948905	-0.88863	-2.858555
10	6	0	-3.144676	-3.152658	-4.182079
11	8	0	-4.373418	2.772512	-4.542522
12	6	0	4.848202	4.960306	-0.561143
13	8	0	5.734962	0.607286	0.396574
14	6	0	-2.077164	-3.494353	3.418018
15	6	0	-1.634485	-6.202607	4.224403
16	6	0	-4.426787	-2.563215	3.446551
17	8	0	-0.169822	-6.348299	6.489204
18	1	0	1.725311	-2.293481	-3.753064
19	1	0	3.415245	1.469566	-3.854189
20	1	0	1.699527	-2.584883	4.176034
21	1	0	-1.549526	1.486141	1.724294
22	1	0	-0.755819	1.23623	4.906362
23	1	0	3.716751	2.075271	4.018703
24	1	0	1.704206	4.456743	2.898129
25	1	0	3.440587	-3.057528	0.349902
26	1	0	0.6938	-4.800054	-0.259641
27	1	0	0.520795	4.866704	-4.168288
28	1	0	-1.060085	4.051448	-1.35147
29	1	0	-1.25498	1.173781	-6.322971
30	1	0	-3.043587	-0.50235	-1.148672
31	1	0	-3.12868	-4.825737	-2.963812
32	1	0	-5.126903	-2.781006	-4.670513
33	1	0	-2.131624	-3.621751	-5.930531
34	1	0	-5.498445	1.651836	-5.456626
35	1	0	5.990849	4.885192	-2.290786
36	1	0	6.082815	5.523543	1.000317
37	1	0	3.404071	6.419031	-0.817042
38	1	0	6.627818	0.466757	-1.200471
39	1	0	-3.452437	-7.188233	4.441174
40	1	0	-0.526043	-7.235603	2.815083
41	1	0	-6.026953	-3.748757	3.955072
42	1	0	-4.864786	-0.617776	2.967031
43	1	0	-1.063419	-5.363819	7.752824

**7_g**			Standard Orientation (A.U.)		
Center number	Atomic number	Atomic Type	X	Y	Z
0	6	0	-0.188022	1.121713	2.975795
1	6	0	2.238167	2.657747	2.608672
2	6	0	3.76109	2.338251	0.135021
3	6	0	2.246659	1.403689	-2.178531
4	6	0	0.916668	-1.264429	-2.02517
5	6	0	1.24717	-2.811217	0.407867
6	6	0	0.069542	-1.762423	2.897683
7	6	0	0.182024	3.237107	-3.130995
8	6	0	-1.693799	1.530342	-4.535817
9	6	0	-1.844764	-0.8351	-2.884204
10	6	0	-3.065304	-3.105071	-4.171835
11	8	0	-4.15283	2.575674	-4.837863
12	6	0	5.074765	4.851171	-0.496915
13	8	0	5.715683	0.521272	0.718856
14	6	0	-2.394727	-3.105045	3.536715
15	6	0	-2.109293	-5.800437	4.43048
16	6	0	-4.700066	-2.087201	3.384233
17	8	0	-0.582455	-5.81457	6.659935
18	1	0	1.808623	-2.419023	-3.504282
19	1	0	3.665194	1.261131	-3.69347
20	1	0	1.377278	-2.292618	4.415865
21	1	0	-1.588863	1.751349	1.595757
22	1	0	-0.971816	1.633761	4.825399
23	1	0	3.567045	2.261212	4.147276
24	1	0	1.720014	4.655563	2.794493
25	1	0	3.270305	-3.058741	0.710376

26	1	0	0.474699	-4.698615	0.037987
27	1	0	0.945317	4.75045	-4.320676
28	1	0	-0.820999	4.133986	-1.557197
29	1	0	-0.899982	1.012188	-6.396096
30	1	0	-3.016721	-0.325194	-1.259491
31	1	0	-3.227478	-4.703568	-2.865962
32	1	0	-4.965879	-2.623926	-4.832393
33	1	0	-1.949203	-3.723934	-5.807945
34	1	0	-4.016997	4.006786	-5.972714
35	1	0	6.30465	4.641137	-2.154435
36	1	0	6.24755	5.440353	1.102134
37	1	0	3.713548	6.355499	-0.901995
38	1	0	6.677032	0.255026	-0.821392
39	1	0	-3.982813	-6.620563	4.802038
40	1	0	-1.211382	-6.947984	2.942523
41	1	0	-6.370745	-3.164441	3.9014
42	1	0	-5.029483	-0.165253	2.750287
43	1	0	-0.023595	-7.535264	6.938829
**7_h**			Standard Orientation (A.U.)		
Center number	Atomic number	Atomic Type	X	Y	Z
0	6	0	0.710083	0.499388	4.78846
1	6	0	2.681608	2.157548	3.477417
2	6	0	2.030259	3.289339	0.880748
3	6	0	2.210322	1.290479	-1.255638
4	6	0	0.255287	-0.950997	-1.356256
5	6	0	-1.375331	-1.591837	0.944787
6	6	0	0.073593	-1.999124	3.454053
7	6	0	2.11659	2.480141	-3.939792
8	6	0	0.641587	0.608858	-5.592512
9	6	0	-1.31524	-0.497266	-3.764956
10	6	0	-2.70639	-2.794778	-4.804933
11	8	0	-0.363617	1.647255	-7.852003
12	6	0	-0.454188	4.783876	0.956307
13	8	0	4.070746	5.058799	0.491797
14	6	0	-1.421012	-3.802197	5.120187
15	6	0	-0.810804	-6.577814	4.801016
16	6	0	-3.185434	-3.062328	6.765412
17	8	0	-0.951723	-7.452881	2.247493
18	1	0	1.394123	-2.654876	-1.729859
19	1	0	4.090877	0.468465	-0.992331
20	1	0	1.883035	-2.931082	2.995155
21	1	0	-1.03474	1.582498	5.071503
22	1	0	1.420044	0.063731	6.685552
23	1	0	4.456857	1.102778	3.27389
24	1	0	3.108875	3.764539	4.713002
25	1	0	-2.393933	-3.333724	0.496469
26	1	0	-2.837673	-0.158234	1.257237
27	1	0	3.988945	2.906206	-4.703927
28	1	0	1.043722	4.259923	-3.930664
29	1	0	1.894112	-0.913923	-6.23503
30	1	0	-2.700048	1.01158	-3.361774
31	1	0	-1.398233	-4.383632	-5.063648
32	1	0	-4.223479	-3.423141	-3.546702
33	1	0	-3.533164	-2.351333	-6.649336
34	1	0	-1.536786	2.971362	-7.363524
35	1	0	-0.37244	6.161182	2.497821
36	1	0	-2.109843	3.586107	1.240569
37	1	0	-0.745896	5.840114	-0.800361
38	1	0	3.798452	5.858433	-1.134862
39	1	0	1.089246	-6.952703	5.574006
40	1	0	-2.1514	-7.734352	5.865784
41	1	0	-4.268502	-4.430259	7.850085
42	1	0	-3.617864	-1.086466	7.110276
43	1	0	0.486268	-6.766533	1.344474
**7_i**			Standard Orientation (A.U.)		
Center number	Atomic number	Atomic Type	X	Y	Z
0	6	0	-0.026912	0.111227	4.281932
1	6	0	2.20936	1.677209	3.323078
2	6	0	2.127352	2.69206	0.598924

3	6	0	2.362553	0.4705	-1.316778
4	6	0	-0.013712	-1.15289	-1.89258
5	6	0	-1.867452	-1.79068	0.245141
6	6	0	-0.650024	-2.349659	2.824564
7	6	0	3.210407	1.202856	-4.015043
8	6	0	0.830557	1.49874	-5.646817
9	6	0	-1.312776	0.128105	-4.182941
10	6	0	-2.854103	-1.696305	-5.809644
11	8	0	0.362283	4.14691	-5.984141
12	6	0	-0.120929	4.470089	0.18834
13	8	0	4.416148	4.166083	0.458325
14	6	0	-2.270483	-4.030903	4.497688
15	6	0	-0.874875	-5.570864	6.467888
16	6	0	-4.789828	-4.114435	4.37774
17	8	0	1.007468	-7.198613	5.419302
18	1	0	0.744531	-2.965547	-2.578106
19	1	0	3.826951	-0.743178	-0.498134
20	1	0	1.12632	-3.372532	2.476764
21	1	0	-1.751173	1.262789	4.333486
22	1	0	0.379994	-0.360204	6.259616
23	1	0	3.956792	0.573388	3.511115
24	1	0	2.434742	3.32078	4.564399
25	1	0	-2.965698	-3.442516	-0.345553
26	1	0	-3.241069	-0.253798	0.473477
27	1	0	4.349103	-0.350701	-4.775761
28	1	0	4.394775	2.890631	-4.117708
29	1	0	1.109768	0.631155	-7.515738
30	1	0	-2.580839	1.602535	-3.462574
31	1	0	-1.646928	-3.193913	-6.58336
32	1	0	-4.359857	-2.603852	-4.716509
33	1	0	-3.74532	-0.721365	-7.407827
34	1	0	-1.245396	4.318994	-6.848264
35	1	0	-1.935248	3.524318	0.468614
36	1	0	-0.104525	5.24228	-1.730078
37	1	0	0.003738	6.029853	1.543232
38	1	0	4.294442	5.204039	-1.046699
39	1	0	-0.022739	-4.314407	7.893847
40	1	0	-2.206537	-6.801495	7.459221
41	1	0	-5.878656	-5.313341	5.641874
42	1	0	-5.868278	-2.998544	3.035059
43	1	0	2.503798	-6.194199	5.104963

	**7_j**		Standard Orientation (A.U.)		
Center number	Atomic number	Atomic Type	X	Y	Z
0	6	0	3.056426	-0.283504	3.33907
1	6	0	3.34018	2.132184	1.756221
2	6	0	3.619245	1.819279	-1.132774
3	6	0	1.074185	1.530903	-2.535521
4	6	0	-0.66651	-0.719453	-1.828928
5	6	0	-1.209353	-1.077467	1.006521
6	6	0	1.013982	-2.215071	2.526677
7	6	0	-0.7196	3.820017	-2.434525
8	6	0	-3.33662	2.829266	-3.292878
9	6	0	-3.1138	-0.106896	-3.320973
10	6	0	-3.052087	-1.160726	-6.01846
11	8	0	-5.364859	3.744262	-1.773718
12	6	0	5.055579	4.089904	-2.204555
13	8	0	5.212229	-0.30928	-1.709255
14	6	0	-0.009216	-3.744122	4.74131
15	6	0	-0.253622	-6.52629	4.188935
16	6	0	-0.761824	-2.756698	6.932933
17	8	0	-1.469631	-7.80712	6.198336
18	1	0	0.143933	-2.50377	-2.520452
19	1	0	1.609119	1.243194	-4.519756
20	1	0	1.918694	-3.587951	1.245505
21	1	0	2.679069	0.317808	5.279889
22	1	0	4.87942	-1.262507	3.401469
23	1	0	5.028506	3.10608	2.460599
24	1	0	1.758029	3.411655	2.141489
25	1	0	-2.825479	-2.364497	1.170137
26	1	0	-1.814906	0.694771	1.90166

27	1	0	-0.060946	5.400552	-3.591026
28	1	0	-0.905213	4.54604	-0.502316
29	1	0	-3.794366	3.517527	-5.187992
30	1	0	-4.775701	-0.893963	-2.364567
31	1	0	-2.91804	-3.227444	-6.009847
32	1	0	-4.768338	-0.636222	-7.051611
33	1	0	-1.431791	-0.431421	-7.084644
34	1	0	-5.084125	3.136637	-0.066337
35	1	0	4.158904	5.868983	-1.650082
36	1	0	5.133435	4.000594	-4.270261
37	1	0	6.992813	4.077118	-1.481203
38	1	0	4.283771	-1.827966	-1.281263
39	1	0	-1.303826	-6.761644	2.397348
40	1	0	1.657477	-7.296626	3.844457
41	1	0	-1.569297	-3.943112	8.393927
42	1	0	-0.606141	-0.748949	7.330612
43	1	0	-1.467325	-9.593765	5.805641

**7_k**			Standard Orientation (A.U.)		
Center number	Atomic number	Atomic Type	X	Y	Z
0	6	0	2.223233	-0.982448	3.604622
1	6	0	1.979974	1.84758	2.992572
2	6	0	3.15061	2.574691	0.420719
3	6	0	1.796275	1.472346	-1.910458
4	6	0	0.834856	-1.30328	-1.879457
5	6	0	-1.114396	-1.899054	0.204999
6	6	0	-0.046242	-2.610823	2.831894
7	6	0	-0.50434	2.943203	-2.918519
8	6	0	-1.789784	1.139747	-4.843548
9	6	0	-0.459837	-1.469946	-4.505066
10	6	0	1.396544	-2.036586	-6.655617
11	8	0	-4.468543	1.030093	-4.56531
12	6	0	3.34539	5.461029	0.214981
13	8	0	5.668385	1.53705	0.248465
14	6	0	-2.191765	-2.642846	4.74415
15	6	0	-4.311352	-4.493204	4.211155
16	6	0	-2.329144	-1.162104	6.786545
17	8	0	-3.429675	-6.977825	3.643621
18	1	0	2.412748	-2.637882	-1.73995
19	1	0	3.25628	1.556866	-3.378702
20	1	0	0.617927	-4.571062	2.675225
21	1	0	2.582076	-1.25201	5.625301
22	1	0	3.906922	-1.679829	2.630225
23	1	0	2.963701	2.95713	4.448317
24	1	0	0.00491	2.471873	3.059236
25	1	0	-2.285541	-3.479804	-0.438067
26	1	0	-2.400674	-0.287015	0.440559
27	1	0	0.019402	4.747533	-3.777107
28	1	0	-1.854116	3.383305	-1.407755
29	1	0	-1.522505	1.825929	-6.775345
30	1	0	-1.913816	-2.945696	-4.520497
31	1	0	0.404845	-2.111	-8.472457
32	1	0	2.870738	-0.590303	-6.80939
33	1	0	2.335596	-3.858898	-6.363437
34	1	0	-4.812069	0.32384	-2.909112
35	1	0	4.578865	6.194755	1.712048
36	1	0	1.500059	6.373391	0.420727
37	1	0	4.156339	6.008936	-1.60731
38	1	0	6.610655	2.155932	1.696832
39	1	0	-5.646454	-4.511604	5.804483
40	1	0	-5.375806	-3.919626	2.530869
41	1	0	-3.918447	-1.293005	8.082988
42	1	0	-0.904863	0.239013	7.246193
43	1	0	-2.456965	-7.54359	5.09154

**7_l**			Standard Orientation (A.U.)		
Center number	Atomic number	Atomic Type	X	Y	Z
0	6	0	3.14743	-2.759871	1.477411
1	6	0	2.141485	-1.035948	3.579406
2	6	0	1.682416	1.787377	2.852203
3	6	0	1.480502	2.220921	-0.033977

4	6	0	-0.600071	0.771827	-1.495219
5	6	0	-1.194861	-1.916505	-0.623377
6	6	0	1.116666	-3.698988	-0.425517
7	6	0	0.99286	5.00449	-0.777615
8	6	0	-1.830657	5.238257	-1.515332
9	6	0	-2.921528	2.53797	-1.473108
10	6	0	-4.844026	2.035987	-3.566386
11	8	0	-3.298441	6.743758	0.188413
12	6	0	-0.544935	2.810439	4.39878
13	8	0	3.920696	3.226371	3.469108
14	6	0	0.153826	-6.35219	0.122851
15	6	0	-0.681108	-7.849543	-2.162996
16	6	0	0.00131	-7.375486	2.427355
17	8	0	1.339816	-8.239287	-3.911841
18	1	0	0.069164	0.65547	-3.463337
19	1	0	3.333783	1.66499	-0.755155
20	1	0	1.991928	-3.786265	-2.302835
21	1	0	4.057221	-4.408438	2.334622
22	1	0	4.649686	-1.766166	0.461392
23	1	0	3.501912	-1.021442	5.144127
24	1	0	0.401898	-1.845951	4.356488
25	1	0	-2.560581	-2.743028	-1.944579
26	1	0	-2.160832	-1.863164	1.208453
27	1	0	2.175579	5.484841	-2.408222
28	1	0	1.508351	6.311903	0.735912
29	1	0	-1.997801	6.035661	-3.429951
30	1	0	-3.881422	2.343591	0.353211
31	1	0	-5.673283	0.145114	-3.407942
32	1	0	-6.388601	3.4117	-3.479182
33	1	0	-3.958326	2.182554	-5.436406
34	1	0	-2.668562	8.461043	0.081608
35	1	0	-0.966996	4.779355	3.94965
36	1	0	-0.1023	2.667371	6.42285
37	1	0	-2.262836	1.707179	4.077075
38	1	0	4.061621	3.243059	5.297341
39	1	0	-1.520523	-9.656633	-1.568492
40	1	0	-2.117865	-6.819999	-3.241646
41	1	0	-0.750291	-9.267706	2.705804
42	1	0	0.599969	-6.381834	4.120459
43	1	0	2.6857	-9.06808	-2.981045

**7_m**			Standard Orientation (A.U.)		
Center number	Atomic number	Atomic Type	X	Y	Z
0	6	0	0.012381	1.086088	2.900747
1	6	0	2.448947	2.598039	2.503555
2	6	0	3.938327	2.257616	0.013117
3	6	0	2.382089	1.34906	-2.282819
4	6	0	1.02014	-1.302292	-2.119776
5	6	0	1.361234	-2.861805	0.303664
6	6	0	0.233128	-1.801056	2.811538
7	6	0	0.328729	3.212028	-3.202404
8	6	0	-1.588415	1.53445	-4.58662
9	6	0	-1.746424	-0.836066	-2.942799
10	6	0	-3.009625	-3.08597	-4.224638
11	8	0	-4.03818	2.612526	-4.847571
12	6	0	5.282206	4.751338	-0.631063
13	8	0	5.869871	0.411333	0.576319
14	6	0	-2.236899	-3.122451	3.466833
15	6	0	-1.953629	-5.798816	4.433685
16	6	0	-4.53809	-2.084315	3.3496
17	8	0	-0.582096	-5.885531	6.759811
18	1	0	1.878654	-2.46456	-3.612172
19	1	0	3.778819	1.19288	-3.816383
20	1	0	1.554972	-2.357613	4.310401
21	1	0	-1.399697	1.731439	1.539679
22	1	0	-0.743405	1.607416	4.760471
23	1	0	3.792529	2.191249	4.02667
24	1	0	1.951928	4.600997	2.691518
25	1	0	3.383358	-3.142033	0.580244
26	1	0	0.553705	-4.73612	-0.056837
27	1	0	1.095514	4.719149	-4.397542

28	1	0	-0.642325	4.117439	-1.613078
29	1	0	-0.829095	1.014033	-6.460257
30	1	0	-2.892118	-0.318387	-1.301649
31	1	0	-1.922815	-3.707646	-5.879097
32	1	0	-3.170694	-4.69	-2.925636
33	1	0	-4.913662	-2.580619	-4.856424
34	1	0	-3.904133	4.037417	-5.990421
35	1	0	6.490839	4.523575	-2.301552
36	1	0	6.481054	5.321682	0.955419
37	1	0	3.939565	6.27626	-1.020917
38	1	0	6.808252	0.123781	-0.974201
39	1	0	-3.822729	-6.687854	4.63091
40	1	0	-0.843846	-6.956628	3.12604
41	1	0	-6.211679	-3.165374	3.854147
42	1	0	-4.86497	-0.152847	2.742365
43	1	0	-1.474415	-4.792141	7.931411

**7_n**			Standard Orientation (A.U.)		
Center number	Atomic number	Atomic Type	X	Y	Z
0	6	0	2.38974	0.739344	3.62502
1	6	0	2.188702	3.0775	1.917853
2	6	0	3.013157	2.781131	-0.866761
3	6	0	0.932451	1.684311	-2.597192
4	6	0	-0.146956	-0.970397	-1.981098
5	6	0	-1.006404	-1.3987	0.76508
6	6	0	1.180017	-1.749459	2.675397
7	6	0	-1.454088	3.314217	-2.934471
8	6	0	-3.473209	1.546665	-4.100653
9	6	0	-2.393397	-1.182266	-3.852105
10	6	0	-1.591699	-2.266167	-6.415775
11	8	0	-5.911636	1.849634	-2.995586
12	6	0	3.857844	5.351686	-1.890066
13	8	0	5.241293	1.231191	-1.039391
14	6	0	0.31826	-3.466597	4.818059
15	6	0	0.926884	-6.230871	4.401631
16	6	0	-0.976321	-2.699508	6.840691
17	8	0	-0.227568	-7.930119	6.111097
18	1	0	1.259021	-2.439049	-2.410081
19	1	0	1.841289	1.516758	-4.455218
20	1	0	2.647826	-2.813866	1.648011
21	1	0	1.544001	1.254984	5.438832
22	1	0	4.387096	0.370931	4.02414
23	1	0	3.370909	4.543435	2.78268
24	1	0	0.255705	3.819325	1.969189
25	1	0	-2.163922	-3.118407	0.784919
26	1	0	-2.25576	0.121936	1.425332
27	1	0	-1.117552	4.99287	-4.091027
28	1	0	-2.164145	3.993946	-1.109539
29	1	0	-3.779025	2.000617	-6.094346
30	1	0	-3.881798	-2.400608	-3.079464
31	1	0	-0.862456	-4.194272	-6.2169
32	1	0	-3.195795	-2.321392	-7.72408
33	1	0	-0.105424	-1.12074	-7.295078
34	1	0	-5.756873	1.440833	-1.21458
35	1	0	2.394453	6.785273	-1.607462
36	1	0	4.289412	5.231367	-3.909981
37	1	0	5.568188	5.954155	-0.896214
38	1	0	4.754791	-0.490082	-0.649348
39	1	0	0.475744	-6.726257	2.423028
40	1	0	2.970578	-6.521462	4.611911
41	1	0	-1.564036	-4.038749	8.278739
42	1	0	-1.459186	-0.73094	7.157787
43	1	0	-2.039897	-7.695736	5.957575

**7_o**			Standard Orientation (A.U.)		
Center number	Atomic number	Atomic Type	X	Y	Z
0	6	0	3.999454	-1.786776	-0.127874
1	6	0	3.648602	-0.530686	2.463226
2	6	0	2.124995	1.988035	2.551289
3	6	0	0.805216	2.645123	0.013234
4	6	0	-1.134344	0.693732	-1.073557

5	6	0	-0.84778	-2.066648	-0.276004
6	6	0	1.68276	-3.296584	-1.071641
7	6	0	-0.662459	5.194923	0.080646
8	6	0	-3.290935	4.660461	-1.057281
9	6	0	-3.744196	1.847606	-0.514091
10	6	0	-5.956753	0.73931	-1.987727
11	8	0	-5.234638	6.30914	-0.218364
12	6	0	0.36744	1.941104	4.861978
13	8	0	4.028072	3.90095	2.967218
14	6	0	1.676023	-6.062515	-0.284052
15	6	0	0.033267	-7.791107	-1.865853
16	6	0	3.004389	-7.026629	1.636858
17	8	0	0.706329	-7.736204	-4.479881
18	1	0	-0.910406	0.739988	-3.142358
19	1	0	2.363445	2.837394	-1.326015
20	1	0	1.721316	-3.301985	-3.14568
21	1	0	5.630343	-3.058518	-0.042228
22	1	0	4.50064	-0.350962	-1.528872
23	1	0	5.500125	-0.07726	3.26498
24	1	0	2.776306	-1.891155	3.755298
25	1	0	-2.403117	-3.137546	-1.12403
26	1	0	-1.080081	-2.258455	1.772304
27	1	0	0.318432	6.708422	-0.92939
28	1	0	-0.948108	5.872375	2.020285
29	1	0	-3.238424	4.932633	-3.111415
30	1	0	-4.125365	1.661818	1.527477
31	1	0	-6.364569	-1.211757	-1.430245
32	1	0	-7.660814	1.8705	-1.672676
33	1	0	-5.555698	0.748343	-4.022609
34	1	0	-5.393454	6.075647	1.594887
35	1	0	-0.561938	3.762594	5.181576
36	1	0	1.492026	1.519232	6.545968
37	1	0	-1.106219	0.500936	4.68787
38	1	0	3.173595	5.524034	2.994961
39	1	0	0.112074	-9.726093	-1.109608
40	1	0	-1.94724	-7.191634	-1.82156
41	1	0	2.900414	-9.02671	2.097987
42	1	0	4.227913	-5.890575	2.827582
43	1	0	2.475558	-8.210388	-4.577693

**7_p**			Standard Orientation (A.U.)		
Center number	Atomic number	Atomic Type	X	Y	Z
0	6	0	0.207292	0.997055	4.381056
1	6	0	-0.337201	3.269506	2.657743
2	6	0	1.426802	3.661165	0.364037
3	6	0	0.695066	2.071278	-1.975361
4	6	0	0.613029	-0.84716	-1.731023
5	6	0	-0.829047	-1.910417	0.558635
6	6	0	0.604522	-1.593207	3.089736
7	6	0	-1.862935	2.74039	-3.214649
8	6	0	-2.686958	0.388713	-4.765241
9	6	0	-0.630154	-1.644942	-4.258974
10	6	0	1.282115	-1.806864	-6.430035
11	8	0	-5.180503	-0.444526	-4.157239
12	6	0	1.441033	6.464783	-0.352638
13	8	0	4.006792	3.133701	1.049558
14	6	0	0.024158	-3.815981	4.821417
15	6	0	1.939426	-5.9222	4.767378
16	6	0	-2.035968	-3.999596	6.265506
17	8	0	2.38457	-6.635104	2.198679
18	1	0	2.534142	-1.629243	-1.626595
19	1	0	2.184174	2.490468	-3.356346
20	1	0	2.617031	-1.774183	2.609496
21	1	0	-1.370397	0.86616	5.712697
22	1	0	1.881564	1.408216	5.527349
23	1	0	-0.228897	4.962198	3.848584
24	1	0	-2.294239	3.185872	1.985301
25	1	0	-1.114559	-3.929264	0.226491
26	1	0	-2.718783	-1.066301	0.7428
27	1	0	-1.731409	4.440341	-4.383006
28	1	0	-3.328333	3.117385	-1.801259

29	1	0	-2.793433	0.82308	-6.783357
30	1	0	-1.553884	-3.49108	-4.079631
31	1	0	2.225275	0.006937	-6.765316
32	1	0	2.748735	-3.210577	-6.021898
33	1	0	0.344091	-2.35606	-8.192711
34	1	0	-5.155122	-1.014598	-2.415072
35	1	0	2.296609	7.559619	1.179285
36	1	0	-0.480092	7.163266	-0.666848
37	1	0	2.550592	6.776424	-2.071043
38	1	0	4.18035	1.316311	1.18778
39	1	0	3.710415	-5.236979	5.631705
40	1	0	1.271984	-7.535791	5.894152
41	1	0	-2.414949	-5.685292	7.375569
42	1	0	-3.421818	-2.492324	6.407086
43	1	0	3.84897	-7.733438	2.169205

**7_q**			Standard Orientation (A.U.)		
Center number	Atomic number	Atomic Type	X	Y	Z
0	6	0	1.230214	0.408472	3.80283
1	6	0	2.598082	2.113901	1.895084
2	6	0	3.794782	0.792101	-0.417046
3	6	0	1.936949	0.414487	-2.625262
4	6	0	-0.516929	-1.127532	-2.157226
5	6	0	-1.979592	-0.642584	0.312667
6	6	0	-0.591968	-1.59593	2.688079
7	6	0	1.063666	2.890638	-3.930568
8	6	0	-1.660559	2.440326	-4.852467
9	6	0	-2.131675	-0.419245	-4.497448
10	6	0	-1.386241	-1.90954	-6.869251
11	8	0	-3.274264	3.963292	-3.298404
12	6	0	6.082528	2.326962	-1.33612
13	8	0	4.6415	-1.719233	0.204106
14	6	0	-2.363634	-2.536005	4.746773
15	6	0	-1.423538	-4.712924	6.336681
16	6	0	-4.616901	-1.500172	5.234619
17	8	0	0.867192	-4.145048	7.658588
18	1	0	-0.05621	-3.147955	-2.195947
19	1	0	3.025304	-0.672778	-4.013703
20	1	0	0.582852	-3.199371	2.11113
21	1	0	0.154255	1.64858	5.071156
22	1	0	2.602149	-0.582861	4.990958
23	1	0	4.08439	3.134096	2.926109
24	1	0	1.304089	3.577269	1.211452
25	1	0	-3.773817	-1.666723	0.143729
26	1	0	-2.480577	1.358222	0.478282
27	1	0	2.322583	3.397104	-5.489847
28	1	0	1.029156	4.502779	-2.637576
29	1	0	-1.906165	2.996127	-6.841668
30	1	0	-4.147068	-0.764481	-4.129214
31	1	0	-1.633253	-3.942704	-6.569769
32	1	0	-2.54976	-1.355748	-8.4917
33	1	0	0.593106	-1.5922	-7.388497
34	1	0	-5.00532	3.509305	-3.695788
35	1	0	5.57102	4.285893	-1.764917
36	1	0	6.900652	1.468134	-3.031633
37	1	0	7.543515	2.371043	0.134807
38	1	0	5.744	-1.586089	1.663348
39	1	0	-2.908222	-5.329203	7.656753
40	1	0	-0.944275	-6.326435	5.129372
41	1	0	-5.811061	-2.19682	6.755459
42	1	0	-5.36439	0.063861	4.133509
43	1	0	0.524737	-2.658446	8.676409

**7_r**			Standard Orientation (A.U.)		
Center number	Atomic number	Atomic Type	X	Y	Z
0	6	0	3.508137	-0.18559	2.815325
1	6	0	1.245874	1.513295	3.477621
2	6	0	0.674385	3.546001	1.460779
3	6	0	-0.379168	2.537194	-1.058917
4	6	0	0.801637	0.142359	-2.278456
5	6	0	0.697111	-2.235716	-0.598333

6	6	0	2.907701	-2.5889	1.269801
7	6	0	-3.235285	1.972961	-1.149273
8	6	0	-3.644463	0.40548	-3.596323
9	6	0	-0.946146	-0.220775	-4.602177
10	6	0	-0.207568	1.442105	-6.855951
11	8	0	-5.178874	-1.777864	-3.207224
12	6	0	-1.118552	5.535027	2.575721
13	8	0	2.989575	4.79617	0.736814
14	6	0	2.456674	-4.889534	2.935058
15	6	0	0.685067	-4.677169	5.1688
16	6	0	3.585239	-7.099998	2.457285
17	8	0	-1.884246	-4.165227	4.474295
18	1	0	2.759983	0.491682	-2.859938
19	1	0	-0.022163	4.09488	-2.378018
20	1	0	4.589204	-3.011938	0.130484
21	1	0	4.498811	-0.741803	4.548473
22	1	0	4.844806	0.967691	1.740697
23	1	0	1.668585	2.519578	5.246161
24	1	0	-0.462565	0.407338	3.853734
25	1	0	0.665046	-3.916106	-1.813597
26	1	0	-1.068022	-2.265938	0.477008
27	1	0	-4.381893	3.691306	-1.129242
28	1	0	-3.839905	0.845053	0.481075
29	1	0	-4.691177	1.499048	-5.004401
30	1	0	-0.932911	-2.192056	-5.239226
31	1	0	-1.499192	1.129695	-8.444767
32	1	0	-0.271962	3.459051	-6.390562
33	1	0	1.713473	1.010866	-7.498031
34	1	0	-4.321772	-2.820775	-1.967641
35	1	0	-0.218566	6.474113	4.190968
36	1	0	-2.881316	4.686967	3.247991
37	1	0	-1.571434	6.979705	1.166033
38	1	0	3.736812	5.479006	2.267896
39	1	0	0.796697	-6.40753	6.312785
40	1	0	1.219913	-3.094085	6.386396
41	1	0	3.259851	-8.757889	3.628303
42	1	0	4.89094	-7.33106	0.887075
43	1	0	-2.414492	-5.540401	3.382676
